# C-terminal lysine residues localise NLRP10 at lipid droplets and govern NLRP10 oligomer formation

**DOI:** 10.1038/s44319-026-00839-9

**Published:** 2026-06-26

**Authors:** Timo-Daniel Voss, Christoph Winterberg, Adrian Beck, Clarissa Gottschild, Leonie Mueller, Selina M Enayat, Matthias Geyer, Thomas A Kufer

**Affiliations:** 1 Department of Immunology, Institute of Nutritional Medicine, University of Hohenheim, Stuttgart, Germany; 2 Institute of Structural Biology, University of Bonn, Venusberg-Campus 1, Bonn, Germany; 3 Present Address: Institute of Nutritional Medicine, University Hospital Schleswig-Holstein, Campus Lübeck, University of Lübeck, Lübeck, Germany

**Keywords:** Immunology, Signal Transduction, Structural Biology

## Abstract

NLRP10 is an atypical member of the NLR family because it lacks a leucine-rich repeat domain at its C-terminus. Here, we show that in human epithelial cells and keratinocytes NLRP10 oligomerises in response to *m*-3M3FBS and SC-10 treatment. NLRP10 co-localises with ASC upon overexpression, but ASC nucleation and recruitment are different to NLRP3. While neither ATP hydrolysis nor the pyrin domain is required, the C-terminal tail region is both necessary and sufficient for oligomerisation. The generation of chimeric proteins shows that the tail region of human and mouse NLRP10 has a conserved function in oligomerisation but determines different protein stabilities. Changes in the subcellular localisation of NLRP10 and oligomerisation are dependent on the presence of evolutionarily conserved lysine residues in the tail region, which localise the majority of NLRP10 to lipid droplets. Our study identifies the C-terminal basic tail of NLRP10 as a key regulatory element for oligomerisation and localisation at lipid interfaces. These findings underline differences in NLRP10 activation with respect to other inflammasome-forming NLRPs and suggest a role of lipids in NLRP10 activation.

## Synopsis

The C-terminal tail of NLRP10 contains evolutionarily conserved lysine residues that mediate NLRP10 oligomerisation at lipid droplets. This might connect NLRP10 to skin diseases, in which NLRP10 levels, lipids and lipid-modifying enzymes are profoundly altered.

- NLRP10 contains a unique C-terminal region with evolutionarily conserved lysine residues characterising it as basic tail.
- The basic tail is both required and necessary for NLRP10 oligomerisation in mice and men.
- NLRP10 oligomers are localised at lipid droplets, a unique feature among the NLR family.

## Abstract (web summary)

The C-terminal tail of NLRP10 contains evolutionarily conserved lysine residues that mediate NLRP10 oligomerisation at lipid droplets. This might connect NLRP10 to skin diseases, in which NLRP10 levels, lipids and lipid-modifying enzymes are profoundly altered.

## Graphical abstract

**Figure:**
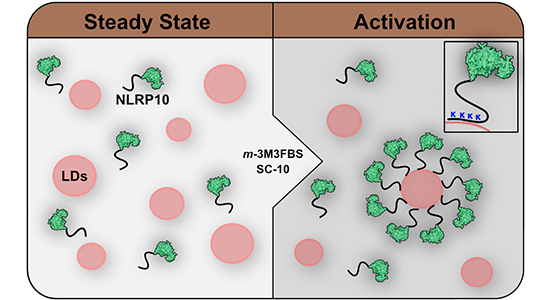

**Figure:**
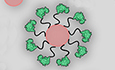

## Introduction

The family of NOD-like receptors containing a pyrin domain (NLRPs) consists of multiple proteins in mammals. NLRPs are characterised by a tripartite domain architecture composed of an N-terminal effector pyrin domain (PYD), a central regulatory NACHT domain (nucleotide-binding and oligomerisation domain present in NAIP, CIITA, HET-E, and TEP1), and usually a C-terminal leucine-rich repeat (LRR) sensory domain. NLRPs of the immune system act as intracellular pattern recognition receptors (PRRs) and detect various damage- and pathogen-associated molecular patterns (DAMPS/PAMPS).

Among the NLRPs, NLRP10 is a unique member as it lacks a LRR domain and shows a non-myeloid expression pattern (Imamura et al, 2010; Wang et al, 2004; Lautz et al, 2012). Together with NLRP1 (Burian et al, 2023), the highest expression of NLRP10 is found in human and mouse skin, especially in keratinocytes (Lautz et al, 2012; Damm et al, 2016). Genome-wide association studies linked NLRP10 to atopic dermatitis (Hirota et al, 2012), and NLRP10 was shown to contribute to contact hypersensitivity (Damm et al, 2016; Miyai et al, 2016). In addition to this, NLRP10 mRNA and protein levels are upregulated in psoriasis (Tervaniemi et al, 2016) and downregulated in atopic dermatitis (Cho et al, 2024). Together, these findings suggest that NLRP10 plays a role in maintaining skin barrier homeostasis. Whether these roles of NLRP10 are mediated by a pattern recognition function is controversially discussed in view of the absent LRR domain, which is usually involved in DAMP/PAMP recognition. First reports suggested a role of NLRP10 as a negative regulator of the NLRP3 inflammasome (Wang et al, 2004; Imamura et al, 2010), where the NACHT domain suppresses ASC activation and subsequent IL-1β release (Imamura et al, 2010). In contrast, it was found that NLRP10 can also enhance NF-κB-mediated inflammatory responses towards the bacterial pathogen *Shigella flexneri* by binding and destabilising the anti-inflammatory regulator Abin-1 (Mirza et al, 2019; Lautz et al, 2012). More recently, it has been shown that both mouse Nlrp10 and human NLRP10 can form inflammasomes and induce IL-1β release in epithelial cells (Zheng et al, 2023; Próchnicki et al, 2023). These two studies showed that NLRP10 is activated by 2,4,6-trimethyl-N-(*m*-3-trifluoromethylphenyl) benzenesulfonamid (*m*-3M3FBS), an inducer of mitochondrial stress. Protein sequence alignment of human and mouse NLRP10 proteins highlights differences in their amino acid composition, with 69% sequence similarity (Zhou, 2025). The structures of the human and mouse NLRP10 PYD domains were determined by NMR spectroscopy, and docking analyses indicated that the two domains may use different surfaces of the PYD to interact with ASC (Su et al, 2013), indicating possible different mechanisms of action as observed, e.g., for NLRP1 (Fink et al, 2008; Moayeri et al, 2012).

In the present study, we characterise the role of both human and mouse NLRP10 in response to *m*-3M3FBS-induced cellular stress. We find that evolutionarily conserved positively charged amino acids in the C-terminal basic tail region direct the association of NLRP10 at lipid droplets and govern the formation of higher-order oligomers. Our study helps to further understand the function of NLRP10 and paves the way for the search for natural activators thereof.

## Results

### Oligomerisation of NLRP10 upon *m*-3M3FBS treatment is ASC- and ATP hydrolysis-independent

**Figure EV1:**
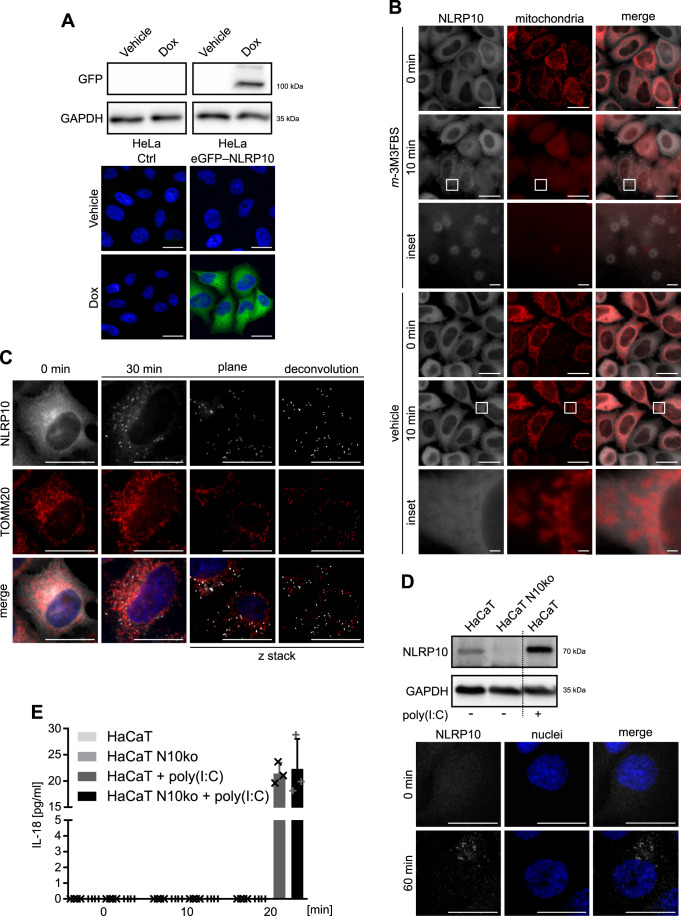
HeLa and HaCaT cells respond with *m*-3M3FBS-dependent NLRP10 oligomerisation without co-localisation with mitochondrial marker TOMM20. (**A**) Stable HeLa cells show expression of eGFP-NLRP10 only in response to Dox. The upper panel shows immunoblot analysis with antibodies directed against GFP and GAPDH as a loading control; the lower panel shows micrographs of cells treated with or without Dox. (**B**) Live-cell imaging micrographs of eGFP-NLRP10 (white) and mts-mCherry (red) expressing HeLa cells treated with 85 µM *m*-3M3FBS or vehicle DMSO. (**C**) Micrographs of eGFP-NLRP10 (green) expressing HeLa cells treated with 85 µM *m*-3M3FBS stained with an antibody directed against TOMM20. Panel 3 shows one plane and panel 4 the deconvoluted z-stack. (**D**) Immunoblot and micrographs of HaCaT and NLRP10-knockout HaCaT (N10ko) cells stained with an antibody directed against NLRP10 and treated with 10 µg/ml poly(I:C) or 85 µM *m*-3M3FBS. (**E**) Quantification of the IL-18 release represented as mean ± SD of HaCaT cells treated with 10 µg/ml poly(I:C) or 85 µM *m*-3M3FBS (0 min, 10 min and 20 min). Nuclei were stained with HOECHST dye (**A**, **C**, **D**). Scale bar: 20 µm or 10 µm (**B**, inset). *N* = 3–4, each measured in duplicates (**E**). Source data are available online for this figure.

**Figure 1:**
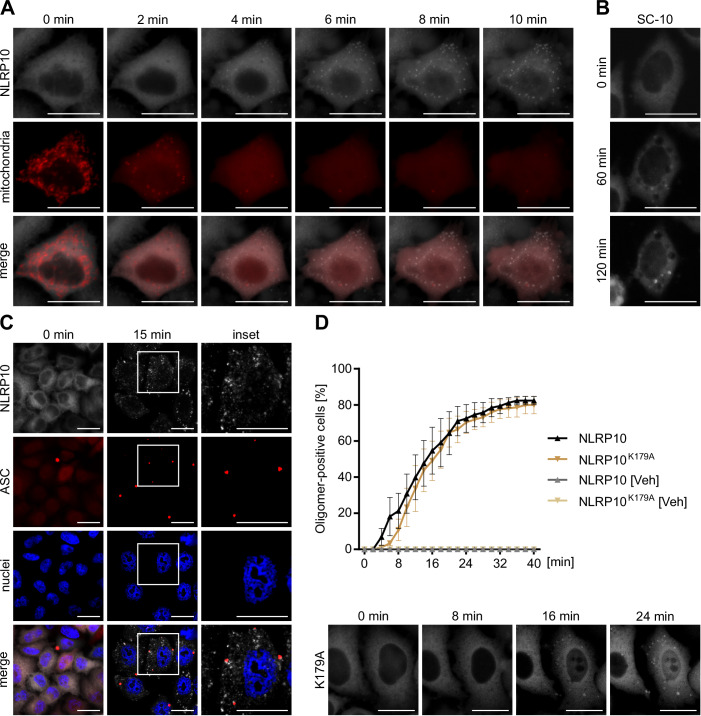
NLRP10 oligomerisation is independent of ASC and ATP-binding. (**A**) Live-cell imaging micrographs of eGFP-NLRP10 (white) and mts-mCherry (red) expressing HeLa cells treated with 85 µM *m*-3M3FBS. (**B**) Live-cell imaging micrographs of eGFP-NLRP10 (white) expressing HeLa cells treated with 100 µM SC-10. (**C**) Representative micrographs of HeLa cells expressing eGFP-NLRP10 (white) and ASC-RFP (red) treated with 85 µM *m*-3M3FBS, nuclei were stained with HOECHST dye. (**D**) Quantification of the oligomerisation kinetics of NLRP10 or NLRP10^K179A^ with representative micrographs below represented as mean ± SD. [Veh] refers to cells treated with *m*-3M3FBS solvent DMSO. Scale bar: 20 µm. *N* = 6 (**D**). Source data are available online for this figure.

**Figure EV2:**
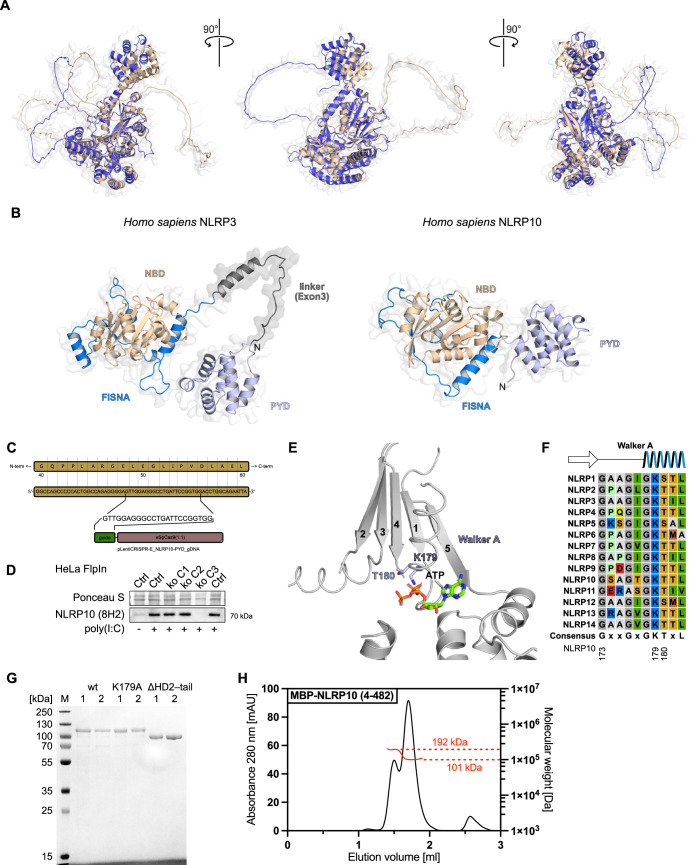
*Homo sapiens* and *Mus musculus* NLRP10 show high similarity with a different PYD-NACHT linker compared to NLRP3. (**A**) Superpositioned AlphaFold models of human (blue) and mouse (beige) NLRP10 at three different angles. (**B**) The linker between the PYD and NACHT is encoded by an additional Exon in NLRP3, which results in a long stretch (grey), which is absent in NLRP10. (**C**) Schematics of the gDNA design to target the DNA anti-sense strand of the NLRP10 PYD. (**D**) Immunoblot analysis of HeLa FlpIn cells transduced with virions produced in Lenti-X 293T cells transfected with the pLentiCRISPR plasmid to target NLRP10 knockout. NLRP10 expression was enhanced using 10 µg/ml poly(I:C). (**E**) Cartoon of the nucleotide-binding site of NLRP10 modelled with AlphaFold3. The location of ATP refers to the model of NLRP3 (PDB: 7PZC). The Walker A motif is indicated with two catalytically active residues. (**F**) Sequence alignment of all NLRP family members with consensus for the Walker A motif. (**G**) SDS-PAGE of the fractions shown in Fig. 2G. (**H**) SEC-MALS measurements of MBP-NLRP10 (4–482) with determined molecular weights for the concentration-dependent monomer-to-dimer transition. Source data are available online for this figure.

In the first report about an NLRP10 inflammasome, the mitochondrial-damaging compound *m*-3M3FBS was used to induce NLRP10 inflammasome formation (Próchnicki et al, 2023). However, the mechanistic details of NLRP10 activation remained elusive. To further explore how NLRP10 is activated and forms inflammasomes, we generated stable HeLa cells that allow expression of N-terminally eGFP-tagged human NLRP10 under the control of a tetracycline-repressor (Fig. EV1A). We used HeLa cells as they are a well-established cell biological model for studying the subcellular localisation of NLR proteins (Kufer et al, 2008; Chen and Chen, 2018). To monitor *m*-3M3FBS-induced mitochondrial damage in live-cell imaging, cells were co-transfected with mScarlet fused to the mitochondrial pre-sequence of human cytochrome c oxidase subunit VIII (Chertkova et al, 2017). We observed that *m*-3M3FBS induced damage of mitochondria within minutes, marked by the release of mScarlet into the cytosol (Fig. 1A). Subsequently, NLRP10 oligomerised in the cytoplasm ~4 min after *m*-3M3FBS was applied. Thereby, NLRP10 formed cytosolically dispersed spherical structures (Fig. EV1B) rather than single specks, typically seen for other inflammasomes such as NLRP3 (Agostini et al, 2004). We did not observe a distinct co-localisation of NLRP10 with mScarlet or the outer mitochondrial membrane marker TOMM20 (Fig. EV1C). To validate that NLRP10 forms spherical structures, we used an alternative activator of NLRP10. In response to SC-10 (Próchnicki, 2021) we observed similar spherical structures within ~60 min (Fig. 1B) showing that these structures are reproducible with alternative activators. To exclude that the absence of NLRP10 specks was related to low expression of ASC in HeLa cells (Masumoto et al, 1999), we generated stable HeLa cells expressing ASC-RFP (Franklin et al, 2014) controlled by a mutated cytomegalovirus promoter (CMVd1), and transiently co-transfected these cells with eGFP-NLRP10. Upon *m*-3M3FBS treatment, the cells showed the formation of both, NLRP10 oligomers and ASC specks, but only some NLRP10 oligomers co-localised with ASC-specks (Fig. 1C). To support our findings, we treated the human keratinocyte cell line HaCaT with *m*-3M3FBS and performed indirect immunofluorescence staining using a NLRP10-specific antibody (Lautz et al, 2012). Consistent with overexpressed NLRP10, endogenous NLRP10 also showed oligomerisation in HaCaT cells (Fig. EV1D), however, no IL-18 release was detected (Fig. EV1E). Next, to analyse whether ATP-binding of NLRP10 is important for the oligomerisation, we used the Walker A mutant K179A, which is conserved in the human NLRPs (Fig. EV2E,F) and abolishes NOD1-mediated functions of NLRP10 (Lautz et al, 2012). Stable cell lines expressing this NLRP10 variant showed no difference in the NLRP10 oligomerisation dynamics upon *m*-3M3FBS treatment compared to wild-type NLRP10 (Fig. 1D). Collectively, our data suggest that NLRP10 oligomerises independently of ATP hydrolysis function and does not form NLRP3-like inflammasomes in our cell models.

### NLRP10 oligomerisation depends on its C-terminal tail region

To further understand the oligomerisation process of NLRP10, we investigated the role of various domains of NLRP10, considering evolutionary conservation. Super-positioning of protein structure predictions of NLRP10 from *Homo sapiens* and *Mus musculus* showed a high degree of structural similarity (Fig. EV2A). In contrast to known inflammasome-forming NLRPs such as NLRP3 or NLRP6, NLRP10 lacks a linker region between the PYD and the NACHT domain, which is encoded by an additional exon in NLRP3 (Tapia-Abellán et al, 2021; Su et al, 2013). The absence of this linker in NLRP10 will most likely result in a conformationally restricted flexibility of the NLRP10 PYD (Fig. EV2B).

**Figure 2:**
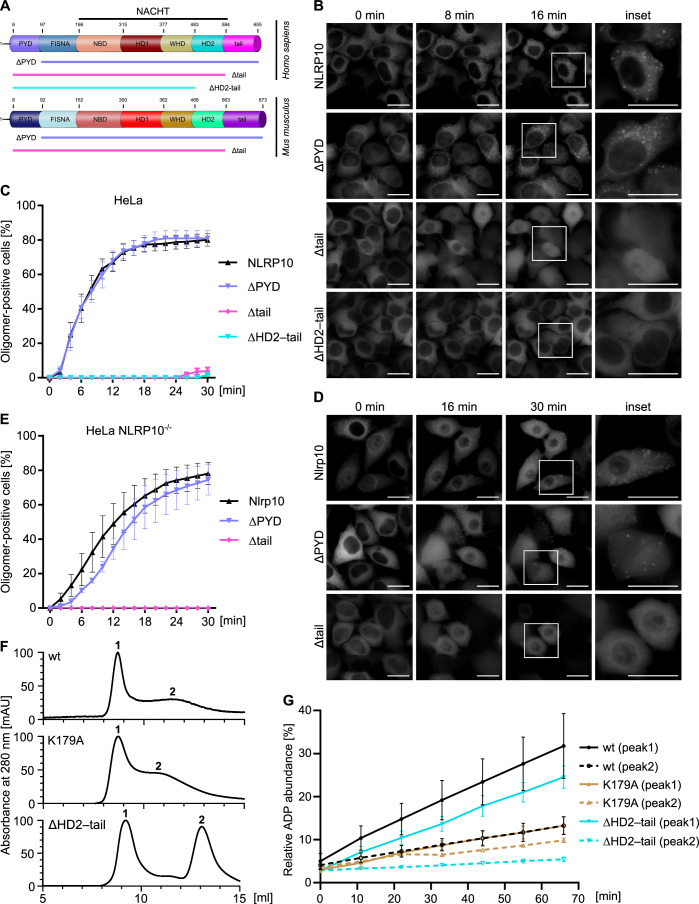
The C-terminal tail is required for oligomerisation of human and mouse NLRP10. (**A**) Schematic representation of *Homo sapiens* and *Mus musculus* NLRP10 and truncation mutants thereof. (**B**) Live-cell imaging micrographs of stable HeLa cells treated with 85 µM *m*-3M3FBS expressing human NLRP10 with quantification (**C**) of the oligomer-positive cells. (**D**) Live-cell imaging micrographs of stable NLRP10-knockout HeLa cells treated with 85 µM *m*-3M3FBS expressing mouse Nlrp10 with quantification (**E**) of the oligomer-positive cells. Quantification (**C**, **E**) is represented as mean ± SEM. (**F**) Size-exclusion chromatography absorbance spectrum of NLRP10 (4–655), K179A mutant or NLRP10ΔHD2–tail (aa 4–482) at 280 nm. (**G**) Relative ADP abundance over time as a measure of the ATP hydrolysis rate of peaks 1 and 2 as mean ± SD measured by ion-paired reverse-phase HPLC from (**F**). Scale bar: 20 µm. *N* = 7–12 (**C**), *N* = 4–8 (**E**), *N *= 2–3 (**G**). Source data are available online for this figure.

NLRP10 lacks the typical LRR at the C-terminus. However, both human and mouse NLRP10 contain a protein region following the conserved helical domain 2 (HD2) of the NACHT (hereafter referred to as “tail”). This tail region is of low sequence complexity and no plausible structure prediction could be obtained for either the human or the mouse NLRP10 sequence (Fig. EV2A), classifying it as an intrinsically disordered region (IDR). To understand how the C-terminal tail and the PYD may influence NLRP10 oligomer formation, we generated deletion constructs of both human and mouse NLRP10 lacking either of these domains (Fig. 2A). Stable HeLa cells expressing either of these NLRP10 truncation proteins, N-terminally fused to eGFP, were analysed for their propensity to form oligomers upon *m*-3M3FBS treatment in live-cell imaging (LCI). Deletion of the PYD did neither change the appearance of oligomers in the cells (Fig. 2B) nor their oligomerisation kinetics (Fig. 2C, black vs. lavender). By contrast, deletion of the tail region (aa 584–655) or of the HD2–tail subdomains (aa 483–655) completely inhibited formation of NLRP10 oligomers (Fig. 2B,C). Next, to analyse the role of the tail region in mouse Nlrp10, we expressed eGFP-tagged mouse Nlrp10 as well as deletion constructs lacking the PYD or the C-terminal tail in HeLa cells in which we knocked out endogenous NLRP10 (Figs. 2D,E and  EV2C,D). Mouse Nlrp10 oligomerised in response to *m*-3M3FBS treatment, with comparable kinetics observed for human NLRP10 (Fig. 2D,E, black). As seen for human NLRP10, deletion of the tail region almost completely inhibited the formation of Nlrp10 oligomers, while deletion of the PYD did not affect oligomerisation (Fig. 2D,E, lavender, magenta).

Seeing that the tail segment was required for oligomerisation, we further explored whether ATP-binding, which is required for an active protein conformation, is also compromised by the deletion of the tail. A construct with a mutated Walker A site (K179A) was used as a control. The corresponding recombinant NLRP10 proteins were expressed in *Sf9* insect cells and purified to homogeneity. Proteins were separated by size-exclusion chromatography, and the two most abundant fractions, peak 1 (void) and peak 2 (NLRP10 oligomers), were collected (Fig. 2F). To exclude contamination, we analysed all proteins via SDS-PAGE and showed that both peaks were purified to homogeneity (Fig. EV2G). Subsequently, ATP hydrolysis was determined by measuring ADP production via ion-paired reverse-phase HPLC analysis. The peak 1 fraction of wild-type NLRP10 showed ATP hydrolysis activity, while the peak 2 fraction showed an about 3-fold reduced hydrolysis rate (Fig. 2G, black lines; *P* < 0.0001), in line with the assumption of an autoinhibited conformation as similarly observed for NLRP3 (Brinkschulte et al, 2022). As expected, the NLRP10 K179A mutant exhibited a reduced ATP turnover rate, both in the peak 1 (wt vs. K179A: *P* < 0.0001) and peak 2 (wt vs. K179A: *P* = 0.2071) fractions (Fig. 2G, ochre lines). The ΔHD2–tail variant again showed ATP hydrolysis activity in peak 1 almost to the same extent as the full-length NLRP10 protein (Fig. 2G, wt (black) vs. HD2–tail (cyan): *P* = 0.9994). Unexpectedly, deletion of the HD2–tail region resulted in NLRP10 monomer formation shown by SEC-MALS measurements (Fig. EV2H) and almost completely blocked ATP hydrolysis (Fig. 2F,G, cyan dashed line, 0 min vs. 66 min *P* = 0.9968), a feature generally described for AAA+ ATPases (Wendler et al, 2012). Collectively, these data show that both human and mouse NLRP10 can form dispersed cytosolic oligomers, which depend on the C-terminal tail region, while the PYD and ATPase functionality are not required for this feature. This assigns a novel regulatory function to the tail region of NLRP10 that is conserved between mice and men.

### Evolutionarily conserved basic residues in the C-terminus of NLRP10 mediate oligomerisation

**Figure EV3:**
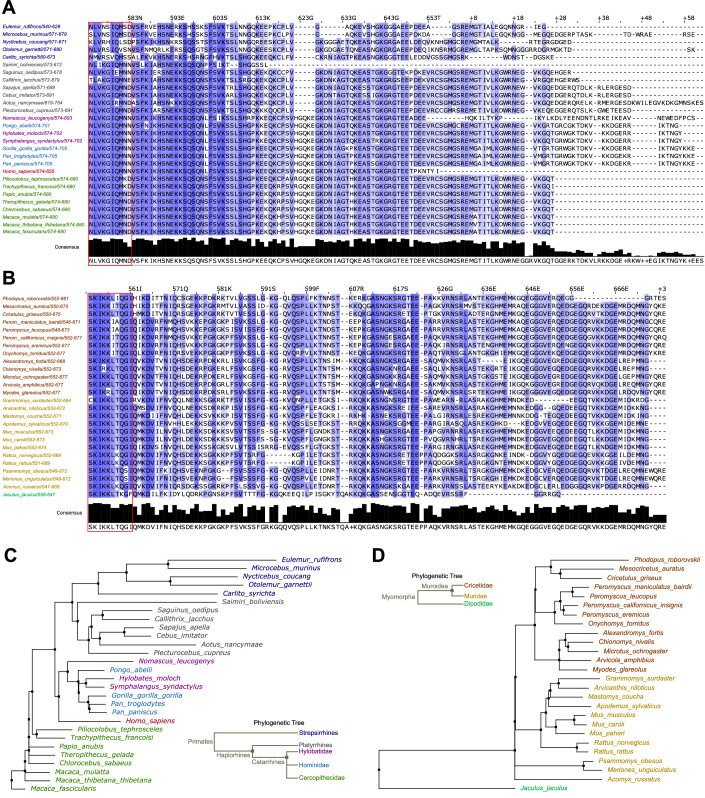
Evolution of the NLRP10 basic tail: *Homo sapiens* NLRP10 shows divergence among Haplorhines. (**A**, **B**) Multi-sequence alignment of primate NLRP10 tails and Myomorpha Nlrp10 tails. The last ten amino acids of the HD2 are shown in a red box. (**C**, **D**) Dendrogram according to NLRP10 sequences of primates or Nlrp10 sequences of Myomorpha. The dendrogram largely matches the general evolutionary distance of the species. The human NLRP10 (red) separates from all other Catarrhines (**C**). Among Myomorpha, only *Jaculus jaculus* separates, currently the only high-quality predicted sequence for Dipodidae (**D**). The colours of (**A**, **B**) match those of (**C**, **D**) and Appendix Tables  Source data are available online for this figure.

The observation that the tail regions of both human and mouse NLRP10 are necessary for oligomerisation suggests an evolutionarily conserved function of this segment. However, the tail sequence in mouse Nlrp10 is longer compared to its human counterpart (112 aa vs. 72 aa). Overall, NLRP10 seems to have undergone striking changes in primates (Fig. EV3A,C), while Myomorpha Nlrp10 evolution is largely represented by the phylogenetic variance (Fig. EV3B,D). Among primates, the human NLRP10 homologue appears to have one of the smallest tail regions; only the Squirrel monkey (*Saimiri boliviensis*) NLRP10 has a shorter tail (Fig. EV3A; Appendix Table S1). Within the Cattharines, the NLRP10 sequence of Cercopithecidae is separate from a mixed cluster of NLRP10 sequence homologues from Hylobatidae and Hominidae (Fig. EV3C). The human NLRP10 very recently diverged from this cluster, specifically a 2.7-kb deletion in the *NLRP10* exon 2 occurred after the human-chimpanzee divergence (Ha et al, 2009), resulting in a shorter tail compared to the other Hominidae.

**Figure 3:**
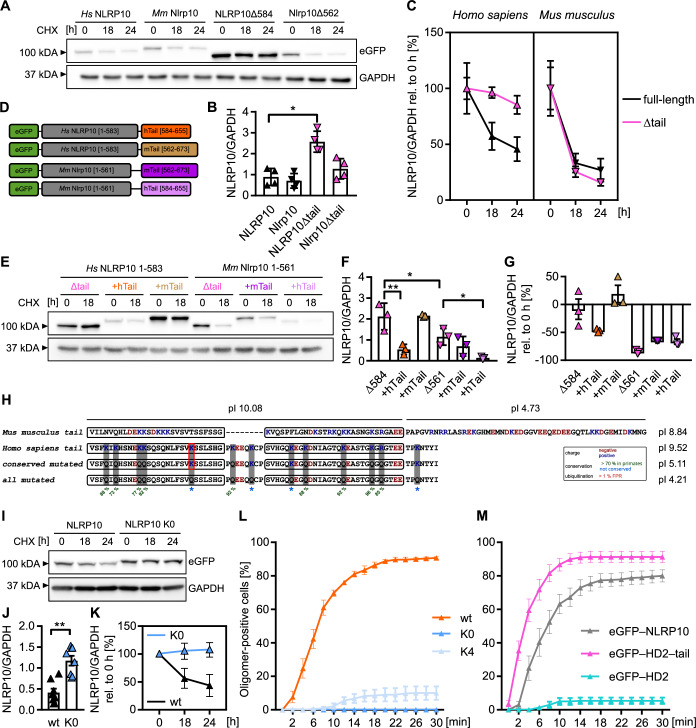
Lysine residues in the basic tail of human NLRP10 govern protein stability and oligomerisation. (**A**) NLRP10 immunoblot image of transiently transfected HeLa cells to analyse the stability of human and mouse NLRP10 and tail truncation mutants. Cells were treated with 20 µg/ml cycloheximide (CHX) 48 h post-transfection (hpt) and lysed after 18 h or 24 h of CHX treatment. (**B**) NLRP10 levels at baseline (48 hpt) normalised to GAPDH as reference. (**C**) Relative change of the protein levels in response to 18 h and 24 h CHX treatment. (**D**) Schematic representation of the chimeric NLRP10 variants used in (**E**–**G**). (**E**) NLRP10 immunoblot image of transiently transfected HeLa cells to analyse the stability of chimeric NLRP10 proteins. CHX treatment was started 48 hpt and lysed after 18 h. (**F**) NLRP10 levels at baseline (48 hpt) normalised to GAPDH as reference. (**G**) Relative change of the protein levels in response to 18 h CHX treatment. (**H**) Alignment of the *Mus musculus* and *Homo sapiens* NLRP10 C-terminus and the mutated C-terminus of the latter. Positively (blue) and negatively (red) charged amino acids are marked. The putative ubiquitination sites are represented by a red box. Conservation was determined with Jalview using all primate NLRP10 sequences. (**I**) NLRP10 immunoblot image of stable HeLa cells to analyse the stability of NLRP10 wt and K0. Cells were treated with CHX 20 h after seeding with 1 µg/ml Dox and lysed after 18 h and 24 h of CHX treatment. (**J**) NLRP10 levels at baseline (20 h Dox) normalised to GAPDH as reference are shown. (**K**) Relative change of the protein levels in response to 18 h and 24 h CHX treatment. (**L**, **M**) Quantification of the oligomer-positive cells in live-cell imaging micrographs of HeLa cells treated with 85 µM *m*-3M3FBS transiently expressing NLRP10 lysine substituted variants (**L**) or NLRP10 HD2 or HD2–tail fused to eGFP (**M**) represented as mean ± SEM. *N* = 4 (**A**–**C**), *N* = 3 (**E**–**G**), *N* = 8 (**J**), *N* = 6 (**K**), *N* = 4 (**L**), *N* = 5–8 (**M**). Statistics: (**B**) Dunn’s (*P = 0.0428) or (**F**) Holm–Sidak’s (Δ584 vs. Δ561 *P = 0.0258, Δ584 vs. +hTail *P = 0.0013, Δ561 vs. +hTail **P = 0.0258) for multi comparison or (**J**) Mann–Whitney *U* for two groups (**P < 0.0011). Source data are available online for this figure.

Sequence analysis showed that the human tail has a higher isoelectric point (pI ~9.5) compared to the mouse (pI ~8.8) (Appendix Tables S1 and2). However, the part of the mouse tail that aligns with the human sequence (aa 566–622) has a very high pI of ~10.1, whereas the remainder of the C-terminal sequence has a pI of ~4.7 (Fig. 3H). The basic character of the tail sequence in human NLRP10 results from twelve lysine residues, the amino acid with the highest abundance in the tail, and one arginine, loosely distributed over the entire length of the sequence, which are opposed by only seven glutamate and one aspartate residues. This positive net value establishes the intrinsically disordered C-terminal region of NLRP10 as ‘basic tail’, which is unique within the family of NLRPs.

N- and C-terminal regions are often involved in the regulation of protein stability (Sharma and Schiller, 2019; Sriram et al, 2011). One mechanism to regulate stability is ubiquitination. Rapid UBIquitination predicted one putative high-confidence ubiquitination site (K605) in the human, but no high-confidence sites in the mouse tail. We expected that the proteins lacking the C-terminus should be more stable than the full-length proteins. Indeed, both human and mouse NLRP10 lacking the basic tail region (NLRP10Δ584 and Nlrp10Δ562) showed higher protein levels in HeLa cells after transfection with equal amounts of expression plasmid. Deletion of the human tail led to a 2.9 ± 0.6-fold increase (*P* = 0.0428) in protein stability, while deletion of the mouse tail did not significantly (1.8 ± 0.7%; *P* = 0.4748) affect protein stability (Fig. 3A,B). When blocking protein de novo synthesis by cycloheximide (CHX) upon 18 h (Fig. 3C), NLRP10 was degraded to 57.1 ± 12.2% (*P* = 0.0054), while deletion of the tail led to a reduction of only ~4% (*P* = 0.9496). Mouse Nlrp10 also showed a high turnover, and protein levels decreased to 33.2 ± 8.5% (*P* < 0.0001) within 18 h (Fig. 3C). However, the deletion of the tail in mouse Nlrp10 did not affect its degradation (25.4 ± 4.7%; *P* < 0.0001) (Fig. 3C).

Next, to corroborate the role of the basic tail from mouse and human NLRP10 in protein stability, we generated chimeric constructs consisting of the human and mouse core (PYD and NACHT) and the mouse or human tail regions, respectively (Fig. 3D). When using the human core fused to the mouse tail, we observed increased protein stability nearly to levels obtained for the tail deletion mutant. By contrast, with its intrinsic human tail, the protein was less stable (Fig. 3E–G). Vice versa, when using the mouse core, fusion to the human tail drastically destabilised the protein (~ 20% compared to the mouse tail). This strongly suggests that the human tail region contributes to protein turnover (Fig. 3E–G).

**Figure EV4:**
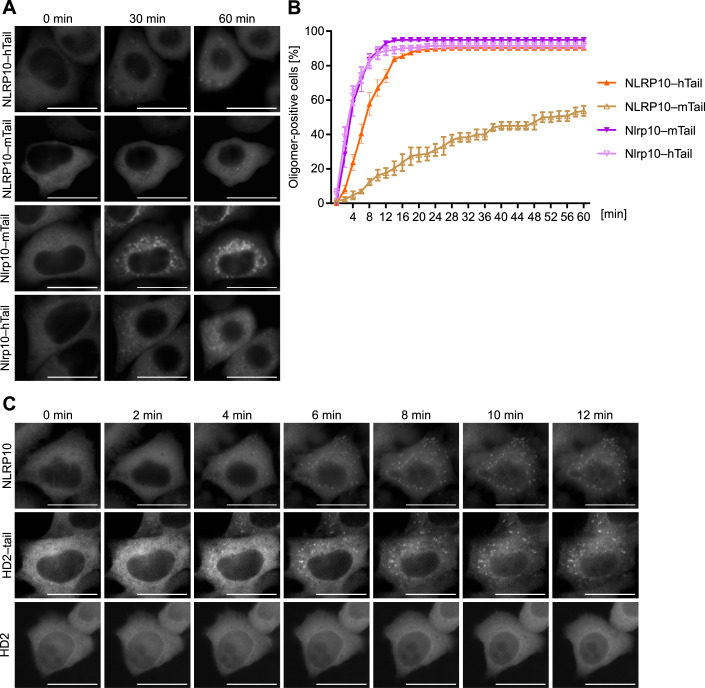
Human and mouse chimeric NLRP10 can oligomerise, and the tail is sufficient for oligomerisation. (**A**) Live-cell imaging micrographs of HeLa cells transiently transfected with chimeric NLRP10 and treated with 85 µM *m*-3M3FBS. (**B**) Quantification of the oligomer-positive cells from (**A**) represented as mean ± SEM. (**C**) Live-cell imaging micrographs of HeLa cells treated with 85 µM *m*-3M3FBS, which were transiently transfected with eGFP fused to the HD2 and HD2–tail of NLRP10 compared to NLRP10 (reused from Fig. 1A). Scale bar 20 µm. *N* = 3–4 (**B**). Source data are available online for this figure.

To understand if the human and mouse NLRP10 basic tail regions are compatible with the core protein for oligomer formation, we transiently transfected HeLa cells with the four chimeric NLRP10 variants and measured NLRP10 oligomer-positive cells upon *m*-3M3FBS exposure as described above. The constructs containing the mouse PYD–NACHT domains showed oligomerisation at a comparable kinetics, regardless of the origin of the tail region (Fig. EV4A). The human NLRP10 core with the human tail oligomerised with kinetics as expected from our results using stable human and mouse NLRP10 expression (Fig. 2C,E). Unexpectedly, fusion of the mouse tail to the human core protein drastically reduced oligomerisation, only leading to 38.3 ± 4.5% at 30 min, within 60 min only half of the cells (53.7 ± 5.0%) showed oligomers (Fig. EV4B).

The unusually high pI of the tail allowed us to hypothesise that the presence of conserved positively charged amino acids facilitates oligomer formation. To test this, we used human NLRP10 and mutated the arginine residue to glutamine and either all twelve, termed NLRP10 K0, or only the nine highly conserved lysine residues to glutamine, termed NLRP10 K4 (Fig. 3H), and evaluated the protein stability and the propensity of these proteins to oligomerise upon *m*-3M3FBS treatment. As observed for NLRP10Δ584, we found higher protein levels (0.41 ± 0.26 vs. 1.18 ± 0.33; *P* = 0.0011) in cells expressing NLRP10 K0 compared to those expressing NLRP10 wt (Fig. 3I,J). Blocking protein de novo synthesis by CHX led to significant NLRP10 degradation to 56.4 ± 18.0% (*P* < 0.0001) and 43.6 ± 19.8% (*P* < 0.0001) within 18 h or 24 h, respectively, while mutation of the positive amino acids stabilised NLRP10 and led to protein levels of 105.0 ± 14.4% and 107.4 ± 13.0% at the same time-points (Fig. 3K).

Next, we analysed whether mutating the lysine and arginine residues to glutamine changes the oligomerisation behaviour of NLRP10. Mutation of all residues (K0) completely inhibited the oligomerisation of NLRP10 in HeLa cells. Deletion of the highly conserved lysine residues only (K4) was sufficient to vastly inhibit oligomerisation to 10.0 ± 8.0% of the cells compared to ~80% observed for NLRP10 (Fig. 3L), suggesting that these amino acids control oligomerisation. Collectively, the highly conserved C-terminal tail of NLRP10 is necessary for protein oligomerisation and stability. To test if the basic tail region is sufficient for oligomerisation, we generated eGFP proteins N-terminally fused to the HD2 domain of human NLRP10 with and without the tail region and transiently transfected HeLa cells with these constructs. Cells were subsequently treated with *m*-3M3FBS, and the oligomerisation behaviour was monitored. Cells expressing the eGFP-HD2–tail construct immediately responded with oligomer formation (Fig. 3M), which resembled the oligomers of NLRP10 (Fig. EV4C). Cells that only expressed the HD2 domain did not respond with oligomer formation, confirming that the tail is necessary and the HD2–tail sufficient for *m*-3M3FBS-induced oligomerisation.

### NLRP10 oligomers are located in lipid compartments

**Figure 4:**
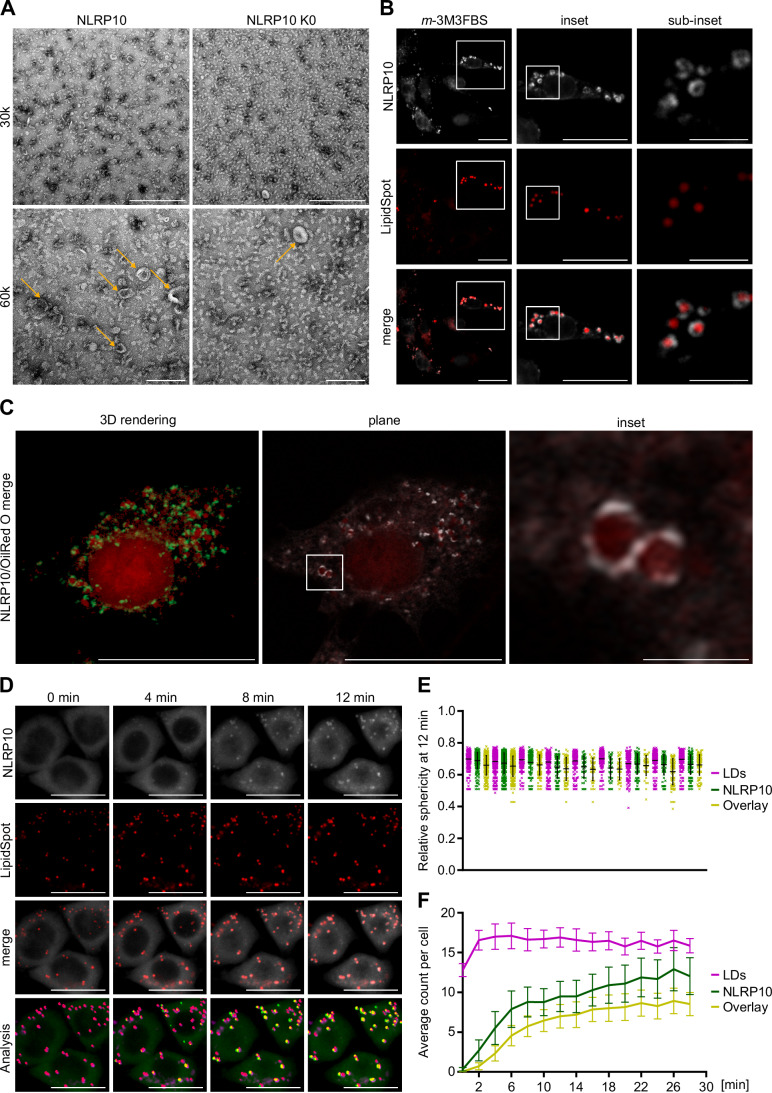
NLRP10 oligomers associate with membranes and lipid droplets. (**A**) Negative stain electron microscopy images of wild-type NLRP10 or the NLRP10 K0 mutant (peak 1) with magnifications of 30k and 60k. Arrows indicate membranes at 60k. (**B**) Micrographs of HeLa cells expressing eGFP-NLRP10 (white), treated with 85 µM *m*-3M3FBS. Lipid droplets were stained with LipidSpot™ 610. (**C**) 3D rendering image of eGFP-NLRP10 (green/white) expressing HeLa cells stained with OilRed O (red) upon treatment with *m*-3M3FBS. (**D**) Representative live-cell imaging micrographs of eGFP-NLRP10 (white) expressing HeLa cells stained with LipidSpot™ 610 (red) upon treatment with *m*-3M3FBS. (**E**) Relative sphericity and (**F**) average count per cell of lipid droplets (LDs), NLRP10 oligomers and the overlay from (**D**) represented as mean ± SD. Scale bar: 500 nm (**A**, upper panel) and 288 nm (lower panel), 20 µm (**B**, left and middle panel, **C**, **D**) or 5 µm (**B**, right panel). *N* = 3 each monitored and analysed in three different frames. Source data are available online for this figure.

**Figure EV5:**
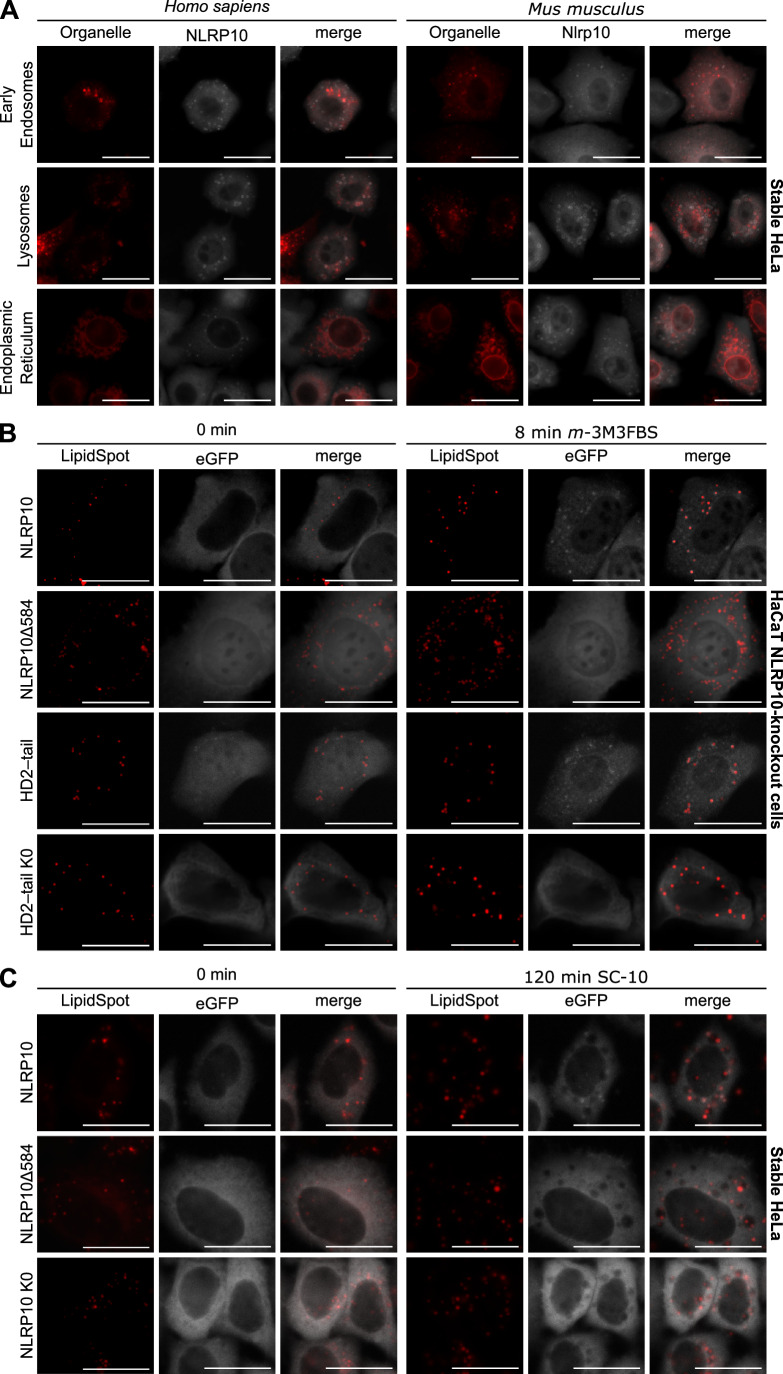
Human and mouse NLRP10 partially co-localised with early endosomes and lysosomes but not with the endoplasmic reticulum. (**A**) Live-cell imaging micrographs (30 min time-point) of HeLa cells expressing human NLRP10 (left) or mouse Nlrp10 (right) treated with 85 µM *m*-3M3FBS. (**B**) Micrographs of NLRP10ko HaCaT cells transiently transfected with eGFP-NLRP10, eGFP-NLRP10Δ584, eGFP-HD2–tail and eGFP-HD2–tail K0 and subsequently treated with 85 µM *m*-3M3FBS. (**C**) Live-cell imaging micrographs of HeLa cells expressing NLRP10 (reused from Fig. 1B), NLRP10Δ584, or the K0 mutant (white) treated with 100 µM SC-10 and stained with LipidSpot™ 610 (red). Scale bar 20 µm.Source data are available online for this figure.

We found that upon *m*-3M3FBS treatment, NLRP10 forms spherical structures (Fig. EV1B) and that basic residues in the tail region are required for oligomerisation (Fig. 3L). This led us to hypothesise that NLRP10 might be recruited by or to lipid compartments to form oligomers at the membrane bilayer. Consistent with this, we found lipid membranes co-purifying with NLRP10 protein expressed in *Sf9* cells, as shown by electron microscopy (Fig. 4A). In contrast, when purifying the NLRP10 K0 protein, lipid membranes were only found to a very low extent (Fig. 4A). To analyse co-localisation of NLRP10 with membranous cellular compartments, we used live-cell imaging with mCherry expression constructs labelling cellular compartments including early endosomes, lysosomes or the endoplasmic reticulum (Fig. EV5A). We observed that NLRP10 only partially co-localised with endosomal and lysosomal markers (both below 50% co-localisation), consistent with the lack of a clear co-localisation of NLRP10 with the ER, endosomal, lysosomal or Golgi compartments in HEK293 cells (Próchnicki et al, 2023).

Inspired by the shape of the NLRP10 structure, we next tested if NLRP10 might be recruited to lipid droplets, cytosolic lipid storage compartments and immune hubs important for integrating energy metabolism and host defence (Farese and Walther, 2025; Bosch et al, 2020). To this end, we treated our stable eGFP-NLRP10 expressing cells with *m*-3M3FBS and stained fixed cells with OilRed O or LipidSpot™ to stain lipid droplets. Here, NLRP10 formed oligomers, which localised at lipid droplets (Fig. 4B). Confocal imaging and 3D rendering showed that NLRP10 clearly localised around lipid droplets (Fig. 4C). We used live-cell imaging (Fig. 4D) and subsequent automated quantification to analyse the kinetics (Fig. 4E,F). We could track both lipid droplets and NLRP10 oligomers over time and found that the sphericity of NLRP10 oligomers was comparable to the sphericity of lipid droplets (Fig. 4E). Lipid droplets were frequent in the cells, and their number did not change upon *m*-3M3FBS treatment (average: 16.23 ± 1.04 lipid droplets per cell; *P *> 0.05; Fig. 4D,F). NLRP10 oligomer formation started at these structures (Fig. 4D), and quantification showed that NLRP10 oligomers (6 min: 7.9 ± 6.5 per cell; *P* = 0.002) and NLRP10/lipid droplet overlay (6 min: 4.6 ± 6.5 per cell; *P* = 0.0404) significantly increased over time. The majority (75.5 ± 2.9%) of NLRP10 oligomers were localised at lipid droplets. Next, we used NLRP10-knockout HaCaT cells and transfected them with eGFP-NLRP10 or the eGFP-HD2–tail construct to analyse if the co-localisation with lipid droplets is conserved in other cells. In comparison to eGFP-NLRP10Δ584 or the HD2–tail K0, we found that NLRP10 or the HD2–tail co-localised with lipid droplets in response to *m-*3M3FBS (Fig. EV5B). In line with this, also SC-10 induced NLRP10 oligomerisation at lipid droplets, but not of NLRP10Δ584 or NLRP10 K0 (Fig. EV5C). Together, our data showed that NLRP10 oligomerised at lipid droplets, identifying a novel role of lipid droplets in the activation process of NLRP10.

## Discussion

**Figure EV6:**
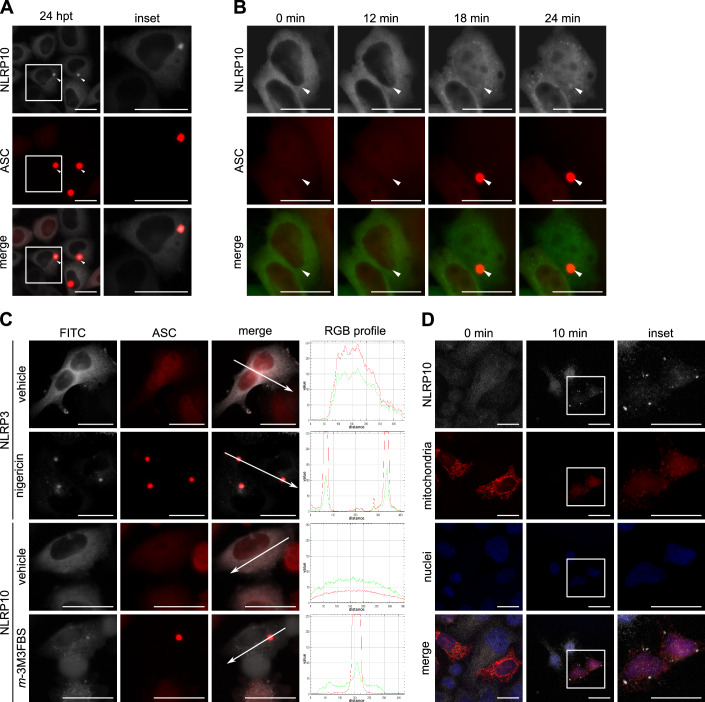
NLRP10/ASC specks are different to NLRP3/ASC specks. (**A**) Live-cell imaging micrographs of stable HeLa cells expressing eGFP-NLRP10 transiently transfected with ASC-RFP (24 h post-transfection). (**B**) Live-cell imaging micrographs of stable HeLa cells expressing ASC-RFP under an CMVd1 promoter transiently transfected with eGFP-NLRP10 (24 hpt) and treated with 85 µM *m*-3M3FBS. White arrows indicate specks. (**C**) Micrographs of stable HeLa cells expressing ASC-RFP under an CMVd1 promoter transiently transfected with myc-NLRP3 or eGFP-NLRP10 (24 hpt) and treated with 10 µM nigericin (120 min) or 85 µM *m*-3M3FBS (12 min), respectively. RGB profile of white lines from the third panel showing ASC signal (red) and NLRP3 or NLRP10 signal (green). (**D**) Micrographs of HeLa cells transiently transfected with mts-mCherry (red) and stained with an antibody (clone 8H2) directed against NLRP10 (white) treated with vehicle or 85 µM *m*-3M3FBS, nuclei were stained with HOECHST dye. Scale bar 20 µm.Source data are available online for this figure.

Human NLRP10 is implicated in inflammatory modulation; however, in contrast to other inflammasome-forming NLRPs such as NLRP3, it is virtually absent in myeloid cells (Lautz et al, 2012). At physiologically relevant levels, it is expressed in keratinocytes (Lautz et al, 2012). This makes keratinocytes and epithelial cells ideal models for studying the function of NLRP10. It is likely that NLRP10 contributes to skin homeostasis. This is supported by a study showing that knockout of NLRP10 significantly reduces epidermal thickness in human skin equivalents (Cho et al, 2024) as well as by our previous finding that NLRP10 expression in keratinocytes is associated with inflammation in type IV hypersensitivity in the skin (Damm et al, 2013). Even though NLRP10 does not contain an LRR domain, its C-terminus consists of 72 amino acids distal to the NACHT domain. This segment, predicted to be intrinsically disordered, is a variation of the transition LRR (trLRR) domain in NLRs which encompasses for example in NLRP3 a 42 residue long acidic loop (Hochheiser et al, 2022). Inflammasome formation is not dependent on the LRRs as shown for NLRP3, which can still be activated via nigericin to form the canonical inflammasome when the LRR is depleted (Hafner-Bratkovič et al, 2018). It therefore came as no surprise that two recent reports suggested that NLRP10 can form an inflammasome (Próchnicki et al, 2023; Zheng et al, 2023). While the first report was mostly focusing on expression in HEK293 and N/TERT cells, the second analysed the physiological role of Nlrp10 in DSS-induced colitis in mice and found a protective role of Nlrp10 expression (Próchnicki et al, 2023; Zheng et al, 2023). If all these functions are associated with inflammasome formation of NLRP10 or might be mediated by inflammasome-independent functions of NLRP10, as we have shown in the context of bacterial infection (Lautz et al, 2012), awaits clarification. In our present study, we focussed to work out NLRP10 intrinsic regulatory mechanisms for oligomer formation. In human HeLa cells and HaCaT keratinocytes, we showed that NLRP10 forms oligomers upon activation and observed a cytosolic dispersed pattern of oligomerised NLRP10, which is reminiscent of a dotted dispersed oligomerisation of NLRP10 seen in primary human keratinocytes (Próchnicki et al, 2023). However, we did not detect downstream IL-18 release. The function of the NLRP10 oligomers needs further clarification, and these could be pre-forms of inflammasomes as observed for NLRP3 (Chen and Chen, 2018). Upon overexpression of ASC in our stable NLRP10 HeLa cells, we confirmed that NLRP10 can stimulus-independently co-localise with ASC specks (Próchnicki et al, 2023) (Fig. EV6A). When we used stable HeLa cells expressing ASC-RFP controlled by a CMVd1 promoter, which has reduced expression levels compared to CMV (Chen et al, 2011), ASC aggregated prior to visible NLRP10 recruitment, suggesting that NLRP10 is not the seed for ASC filament formation in this cell system (Fig. EV6B). In the same cell model, we also overexpressed NLRP3 and induced inflammasome formation by nigericin. We observed a clear difference between NLRP3 and NLRP10 inflammasome formation (Fig. EV6C). This, together with the lack of a flexible linker between the PYD and NACHT domain in NLRP10, strongly suggests that NLRP10 might not form a NLRP3-like inflammasome.

Our results demonstrate that the C-terminal tail is both necessary and sufficient for oligomerisation of NLRP10 (Figs. 2 and 3). This process is linked to changes in the subcellular localisation of NLRP10 oligomers within the cells. It was suggested that NLRP10 oligomers co-localise with mitochondria (Próchnicki et al, 2023). Using our eGFP-NLRP10 construct or immunostaining of endogenous NLRP10 (Fig. EV6D), we could not detect a clear mitochondrial co-localisation in our cells. By contrast, we observed localisation of NLRP10 oligomers at lipid droplets (OilRed O or LipidSpot-positive structures) and partial co-localisation with early endosomes and lysosomes. Furthermore, we observed an enrichment of lipid membranes in fractions of purified NLRP10 protein expressed in *Sf9* cells, suggesting that NLRP10 can bind to membranes (Fig. 4). Based on our observation that lysine residues in the tail of NLRP10 are needed for oligomer formation and subcellular reorganisation, we hypothesise that the tail mediates localisation at lipids. Indeed, we could show that a construct containing the HD2 and tail domains was sufficient to localise at membranes. Notably, a prerequisite for this was the treatment of the cells with *m*-3M3FBS. We assume it is unlikely that the HD2–tail has the propensity to detect any triggers and thus propose that changes in the composition of membrane lipids are the actual signal for NLRP10 oligomerisation. This is supported by a study on the role of lysine clusters in peptides that bind to the membranes with a sigmoidal dependence (Mosior and McLaughlin, 1992), which matches our oligomerisation kinetics. Furthermore, NLRP3 harbours a lysine stretch, which is important for its trans-Golgi localisation (Chen and Chen, 2018). In addition, we showed before that NLRP10 can localise to the plasma membrane of HeLa cells upon bacterial infection and is found in flotillin-2 positive membrane fractions when co-expressed with NOD1 (Lautz et al, 2012). Taken together, we provide evidence that the basic tail region of NLRP10 anchors the protein at membranes.

Asking if this mechanistic feature of NLRP10 is evolutionarily conserved, we showed that the mouse Nlrp10 also forms oligomers upon *m*-3M3FBS. Even though the tail region of mouse Nlrp10 is substantially longer, the oligomerisation was engaged in a similar manner. The functions of the tail regions were confirmed by chimeric proteins consisting of mouse tail and human core NLRP10 fusions, and vice versa. Unexpectedly, the mouse tail was not fully compatible with the human core, as this chimeric construct showed reduced oligomerisation kinetics. This observation might be explained by core-tail interactions. Interestingly, the human NACHT domain contains a sequence (FEEKLKKRGL, aa 258-267) just following the Walker B motif, which is absent in mouse Nlrp10. This polar region is supposed to form an exposed loop, whose overall basic charge could possibly attract the extended acidic tail region of mouse Nlrp10 (Fig. 3H). On the other hand, this might be a species-specific effect and further studies will show if different stimuli induce NLRP10 oligomerisation in mice and men. Previously, differences in the PYD between mouse and human NLRP10 were reported (Su et al, 2013) and one could speculate these might be one driver for the fact that the mouse core protein is less stable than the human core. In Muroidea, the evolution of NLRP10 has not been as diverse as in primates. Both the sequence conservation as well as the length and pI of the basic tails, largely resemble the evolutionary conservation of the subfamilies, where all Creticidae and Muridae form a distinct cluster and both separate from *Jaculus jaculus* (Fig. EV3B,D). We cannot explain how the presumed divergence that changes the picture in primates has happened and if this has an influence on the function. However, the high conservation of the lysine residues suggests that the localisation of NLRP10 at lipid compartments is conserved. The interesting finding that the mouse tail fused to the human core leads to slowed oligomerisation allows us to speculate that the prolonged tail might stabilise the autoinhibited state of the protein and it will be of interest to analyse other primate NLRP10 candidates in their oligomerisation behaviour including *Saimiri boliviensis* (shortest protein and tail), *Aotus nancymaae* (longest protein) and, e.g., *Pan paniscus* (longest tail). This would also help to understand the physiological role of NLRP10, its oligomerisation and localisation.

Taken together, our work identifies the C-terminal basic tail of NLRP10 as an important regulatory element for NLRP10 activation. Furthermore, we provide evidence that this domain facilitates NLRP10 oligomerisation at lipid-rich compartments in the cells. Linking NLRP10 to a ‘sensing’-function of lipids will help identify the activation cues which likely converge at affecting lipid composition of cellular membranes. Moreover, our data emphasises the significance of lipids in the activation processes of NLRs, as demonstrated by the interaction of NLRP3 with PI(4)P (Zhang et al, 2023) and by the NOD1/2 interaction with sphingosine-1 phosphate (S1P) (Pei et al, 2021). This will also provide novel insights into the role of NLRP10 in skin disease, such as atopic dermatitis, which is linked to profound alterations in cellular lipids and lipid-modifying enzymes (Danso et al, 2017).

## Methods

**Table:** Reagents and Tools Table

Reagent/Resource	Reference or Source	Identifier or Catalog Number
Experimental Models		
HeLa Flp-In T-REx	Hentze Lab (EMBL Heidelberg, GER)	
HaCaT	Lab stock	
HaCaT NLRP10 knockout	This study	
Lenti-X 293T	Thermo Fisher Scientific	#NC9834960
Sf9	Thermo Fisher Scientific	#12659017
*E. coli* DH5α	Lab stock	[F- Φ80lacZΔM15 Δ(lacZYAargF) U169 deoR recA1 endA1 bsdR17 (rk+, mk+) phoA supE44 thi-1 gyrA96 relA1 λ-]
HeLa eGFP-NLRP10	This study	
HeLa eGFP	Lab stock	
HeLa eGFP-NLRP10Δ584	This study	
HeLa eGFP-NLRP10Δ483	This study	
HeLa eGFP-NLRP10 K179A	This study	
HeLa eGFP-NLRP10 K0	This study	
HeLa eGFP-NLRP10ΔPYD	This study	
HeLa ASC-RFP CMVd1	This study	
HeLa Flp-In NLRP10 knockout (HeLa^ko^)	This study	
HeLa^ko^ eGFP-Nlrp10	This study	
HeLa^ko^ eGFP-Nlrp10Δ561	This study	
HeLa^ko^ eGFP-Nlrp10ΔPYD	This study	
**Recombinant DNA**		
pcDNA5/FRT/TO (pcDNA5)	Thermo Fisher Scientific	#V652020
pOG44	Thermo Fisher Scientific	#V600520
mCherry-Lysosomes-20	Michael Davidson	RRID:Addgene_55073
mCherry-Sec61β	Stephen Royle	RRID:Addgene_172445
pFX-mCherry-EEA1	Yusuke Ohba	RRID:Addgene_174452
4xmts-mScarlet-I	Dorus Gadella	RRID:Addgene_98818
pLentiCRISPR-E	Phillip Abbosh	RRID:Addgene_78852
psPAX2	Didier Trono	RRID:Addgene_12260
pMD2.G	Didier Trono	RRID:Addgene_12259
pLentiCRISPR-E_gDNA-NLRP10	This study	
pcDNA5-eGFP	Lab stock	
pcDNA5-eGFP-NLRP10	This study	
pcDNA5-eGFP-NLRP10 K197A	This study	
pcDNA5-ASC-RFP CMVd1	This study	
pcDNA5-eGFP-NLRP10ΔPYD	This study	
pcDNA5-eGFP-NLRP10Δ584	This study	
pcDNA5-eGFP-NLRP10Δ483	This study	
pcDNA5-eGFP-Nlrp10	This study	
pcDNA5-eGFP-Nlrp10Δ561	This study	
pcDNA5-eGFP-Nlrp10ΔPYD	This study	
pcDNA5-eGFP-HsNLRP10-hTail	This study	
pcDNA5-eGFP-HsNLRP10-mTail	This study	
pcDNA5-eGFP-MmNlrp10-hTail	This study	
pcDNA5-eGFP-MmNlrp10-mTail	This study	
pcDNA5-eGFP-NLRP10 K0	This study	
pcDNA5-eGFP-NLRP10 K4	This study	
pcDNA5-eGFP-HD2--tail	This study	
pcDNA5-eGFP-HD2—tail K0	This study	
pcDNA5-eGFP-HD2	This study	
pcDNA3.1-myc-NLRP3	Roland Wagner	
pACE-Bac1-MBP-tev-NLRP10 (FL,4-655)	This study	
pACE-Bac1-MBP-tev-NLRP10 (FL,4-655) K179A mutant	This study	
pACE-Bac1-MBP-tev-NLRP10 (FL,4-655) K0 mutant	This study	
pACE-Bac1-MBP-tev-NLRP10 (C-tail mutant,4-482)	This study	
**Antibodies**		
rat IgG2aκ anti-NLRP10 8H2	Lautz et al, 2012	RRID:AB_3713361
mouse IgG1κ anti GFP antibody	Merck-Roche	#11814460001; RRID:AB_390913
rabbit IgG anti-TOMM20 (D8T4N)	Cell Signaling Technologies	#42406; RRID:AB_2687663
mouse anti GAPDH	Santa Cruz Biotechnology	#sc-47724; RRID:AB_627678
Goat anti-Rat IgG (H+L) Highly Cross-Adsorbed Secondary Antibody, Alexa Fluor™ Plus 488	Thermo Fisher Scientific	#A48262TR; RRID:AB_2896331
Goat anti-Rabbit IgG (H+L) Highly Cross-Adsorbed Secondary Antibody, Alexa Fluor™ 546	Thermo Fisher Scientific	#A-11035; RRID:AB_143051
HRP-conjugated goat anti mouse IgG antibodies	Bio-Rad Laboratories	#170-6516; RRID:AB_11125547
HRP-conjugated goat anti rabbit IgG antibodies	Bio-Rad Laboratories	#170-6515; RRID:AB_11125142
**Oligonucleotides and other sequence-based reagents**		
CAAGGGATCCATGGCCATGGCC	Eurofins Genomics	NLRP10_F
TAGACTCGAGTTATATGTAAGTATTTTTTGGTG	Eurofins Genomics	NLRP10_R
GTGGTCGACGATATCTTAATTGTTCATCTGAATACC	Eurofins Genomics	NLRP10_1-583_R
GTGGTCGACGATATCTTACTCTTTCACCAGGTAAGACATGG	Eurofins Genomics	NLRP10_1-482_R
GCGCGGATCCTACAGAGAAGTATACCGAGAGCATGTG	Eurofins Genomics	NLRP10_ΔPYD_F
GAAGATCTATGGCCTTGGCACGGGCCAA	Eurofins Genomics	Nlrp10_BglII_F
GATCCTCGAGCTACCCATTCATC	Eurofins Genomics	Nlrp10_XhoI_R
GAAGATCTATGGAGCTTGTAGACTACCTCA	Eurofins Genomics	Nlrp10dPYD_BglII_F
GATCCTCGAGTTAGATACCTTGTGTGAGCTTCT	Eurofins Genomics	Nlrp10Δtail_XhoI_R
TAGACTCGAGATTGTTCATCTGAATACC	Eurofins Genomics	NLRP10_nsc_XhoI
GATCCTCGAGGATACCTTGTGTGAGCTTCT	Eurofins Genomics	Nlrp10Δtail_nsc_XhoI_R
GATCCTCGAGGTATCATTCAAGATAAAACATTC	Eurofins Genomics	XhoI_hTail_F
GTGGGCCCTTATATGTAAGTATTTTTTGGTG	Eurofins Genomics	hTail_ApaI_R
GATCCTCGAGCAGATGAAAGATGTCATTCTC	Eurofins Genomics	XhoI_mTail_R
CTGGGCCCCTACCCATTCATCTTATCTATCATC	Eurofins Genomics	mTail_ApaI_R
GTTGGAGGGCCTGATTCCGGTGG	Eurofins Genomics	gDNA NLRP10
**Chemicals, Enzymes and other reagents**		
*m*-3M3FBS	Tocris	#1941/10
*m*-3M3FBS	MedChem Express	#HY-19619
SC-10	MedChem Express	#HY-100931
LipidSpot™ 610	Biotium	#70065-T
bis-Benzimid H 33258	Merck	#Hoechst 33258
**Software**		
Fiji		RRID:SCR_002285
Jalview		RRID:SCR_006459
ChimeraX	UCSF	RRID:SCR_015872
Inkscape (v1.4; 86a8ad7, 2024-10-11		RRID:SCR_014479
Prism 7 software	GraphPad	RRID:SCR_002798
**Other**		
Phusion High-Fidelity PCR master mix	Thermo Fisher Scientific	#F531
PCR/Gel purification kit	Macherey-Nagel	#740609
ROTI®GelStain red	Carl Roth	#0984
T4 DNA ligase	Thermo Fisher Scientific	#EL0011
NucleoBond Xtra Midi Kit	Macherey-Nagel	#740410
NucleoSpin Plasmid Mini Kit	Macherey-Nagel	#740588
Opti-MEM™ I	Thermo Fisher Scientific	#31985070
FluoroBrite	Thermo Fisher Scientific	#A1896701
Lipofectamine™ 2000	Thermo Fisher Scientific	#11668027
Clarity Western ECL Substrate	Bio-Rad Laboratories	#1705061
human total IL-18 DuoSet ELISA	Biotechne	#DY318
MBPtrap column	GE Healthcare	#28918780
Superose 6 Increase 10/300 GL column	GE Healthcare	#29-0915-96
Superdex 200 Increase 10/300 GL column	GE Healthcare	#28-9909-44

### Molecular cloning

#### PCR

In total, 50 ng template DNA were mixed with primers (Appendix Table S3) and Phusion High-Fidelity PCR master mix (#F531, Thermo Fisher Scientific (TFS); Waltham, MA, USA) according to the manufacturer’s protocol. PCR products were analysed by gel electrophoresis. PCR products were purified using the PCR/Gel purification kit (#740609, Macherey-Nagel; Dueren, GER) according to the manufacturer’s protocol, and DNA was eluted in nuclease-free water.

#### Agarose gel electrophoresis

To separate DNA fragments, 0.5% agarose (#3810.2, Carl Roth; Karlsruhe, GER) in TAE buffer [40 mM Tris (pH 7.6), 20 mM acetic acid, 1 mM EDTA] gels were used. ROTI®GelStain red (#0984, Carl Roth) was added directly in the agarose gel and separation was performed at 10 V/cm for 25–60 min. DNA was visualised with UV light (365 nm) using the Fusion FX Camera System (Vilber Lourmat). The size of DNA fragments was estimated by Gene Ruler 100 bp plus and GeneRuler 1 kb DNA ladders (#SM0321 and #SM0311, TFS) as standards.

#### Restriction digest

PCR products and vectors were cloned using FastDigest restriction enzymes with 10x FastDigest (FD) green buffer (both TFS) in a total volume of 20–30 μl at recommended conditions.

#### DNA ligation

T4 DNA ligase (#EL0011, TFS) was used as recommended in a total volume of 20 μl for 10 min at room temperature, followed by 5 min heat-inactivation at 70 °C. The digested and purified insert was applied in a 1:3 mass/size ratio to the digested and purified expression vector.

#### Mutagenesis

The K179A mutation of NLRP10 has been published previously (Lautz et al, 2012). Plasmids carrying the NLRP10 tails with deleted lysine and arginine residues were generated at Eurofins Genomics and then sub-cloned into the NLRP10Δ584 plasmid in a pcDNA5/FRT/TO backbone with a deleted stop codon.

#### Transformation and DNA isolation

For amplification of mammalian expression plasmids, 10 μl of the ligation mix was combined with 50 μl chemically competent *E. coli* DH5α [F- Φ80lacZΔM15 Δ(lacZYAargF) U169 deoR recA1 endA1 bsdR17 (rk + , mk + ) phoA supE44 thi-1 gyrA96 relA1 λ-]. The solution was gently mixed and incubated on ice for 30 min. Bacteria were heat-shocked for 30 sec at 42 °C and immediately placed on ice for 5 min before 1 ml pre-warmed lysogeny broth (LB) was added to the bacteria. After incubation for 1 h at 37 °C and 400 rpm bacteria were pelleted at 3000 × *g* for 5 min and 800 μl of the medium was removed. The bacteria were resuspended in the remaining 250 μl LB and spread on a LB agar plate containing 50 μg/ml kanamycin or 100 µg/ml ampicillin and incubated overnight at 37 °C. Single colonies were expanded in 7 ml LB containing antibiotics, and DNA was extracted with the NucleoBond Xtra Midi Kit (#740410, Macherey-Nagel) or the NucleoSpin Plasmid Mini Kit (#740588, Macherey-Nagel), according to the manufacturer’s protocol. Plasmid DNA was reconstituted or eluted with endotoxin-free water. Plasmids were digested to test for successful integration of inserts and sequenced (Eurofins Genomics; Ebersberg, GER). Plasmids for organelle tracking: mCherry-Lysosomes-20 (kindly provided by Michael Davidson (RRID:Addgene_55073), mCherry-Sec61β kindly provided by Stephen Royle (RRID:Addgene_172445) (Ferrandiz et al, 2022), pFX-mCherry-EEA1 (kindly provided by Yusuke Ohba, (RRID:Addgene_174452) (Kashiwagi et al, 2019), 4xmts-mScarlet-I (kindly provided by Dorus Gadella RRID:Addgene_98818) (Chertkova et al, 2017). The myc-NLRP3 plasmid was kindly provided by Roland Wagner.

### Cell culture

All cells were routinely passaged twice or thrice a week and kept at 37 °C, >95% rH and 5% CO_2_ in Dulbecco’s Modified Eagle Medium (DMEM) supplemented with 10% foetal bovine serum (FBS). Cell mycoplasma contamination is regularly checked by PCR, and cell line authentication was performed in February 2023 by Multiplexion GmbH.

### Generation of stable HeLa cell lines

The maternal HeLa Flp-In T-REx cells were kindly provided by the Hentze Lab (EMBL Heidelberg, GER) and allow doxycycline-inducible expression of various NLRP10 proteins sub-cloned into the pcDNA5/FRT/TO (#V652020, TFS) expression vector. To generate stable cells, 7.5 × 10^5^ maternal HeLa cells were seeded on a six-well plate in 3 ml growth medium. The next day, the medium was changed to 2.5 ml fresh growth medium and cells were co-transfected using Lipofectamine 2000 according to the manufacturer’s protocol. Briefly, 400 ng of the pcDNA5/FRT/TO with the respective insert were mixed with 3.6 µg pOG44 (#V600520, TFS) plasmid in 250 µl Opti-MEM™ I (#31985070, TFS). 10 µl Lipofectamine 2000 (#11668019, TFS) in 250 µl Opti-MEM were added to plasmid DNA and incubated for 20 min, mix was added dropwise to the cells. The following day, cells were detached and spread onto three 10 cm dishes in a total of 10 ml growth medium each. 72 h after spreading, selection antibiotics 500 µg/ml hygromycin and 10 µg/ml blasticidin were added, and the medium was changed every 2–3 days. Within ~14 days, single-cell colonies appear, and they were transferred into 100 µl trypsin-EDTA in 96-well plates to allow cells to separate from each other. Cells were subsequently expanded in a 96-well, then 24-well and 6-well. Cells were then analysed for successful integration at the locus by fluorescence microscopy, immunoblot analyses and β-galactosidase assay. Single clone cultures were used for experiments.

### Generation of NLRP10 knockout cell lines

NLRP10-specific gDNA was ligated into pLentiCRISPR-E (Addgene plasmid #78852; RRID:Addgene_78852). For lentiviral production, Lenti-X 293T (#NC9834960, TFS) cells were transiently transfected with 10 µg plentiCRISPR-E_gDNA-NLRP10, 7.5 µg psPAX2 (Addgene plasmid #12260; RRID:Addgene_12260) and 5 µg pMD2.G (Addgene plasmid #12259; RRID:Addgene_12259) using Lipofectamine 2000. Virus-containing supernatants were collected 48 h and 72 h after transfection, pooled and filtered. 400 µl were used to transduce HaCaT or HeLa Flp-In cells. Cells were selected for 5 days using 500 ng/ml puromycin. Subsequently, for HeLa cells, single-cell clones were picked and characterised. HaCaT cells were used as a knockout pool.

### Live-cell imaging (LCI) and microscopy

HeLa Flp-In eGFP-NLRP10 cells were seeded on a glass-bottom culture dish with four compartments (Greiner) at a density of 10^5^ cells per subdivision in 500 μl growth medium supplemented with 1 µg/ml Dox. Prior to *m*-3M3FBS (#1941/10, Tocris Bioscience, Bristol, UK; # HY-19619, MedChemExpress; Monmouth Junction, NJ, USA) or SC-10 (#HY-19619, MedChemExpress) treatment and LCI, cells were washed with 500 μl FluoroBrite (#A1896701, Gibco, TFS) and then incubated in 400 μl FluoroBrite supplemented with 2 mM L-glutamine for 30 min at 37 °C, >95% rH and 5% CO_2_. To stain lipid droplets, 0.5 µl LipidSpot™ 610 (#70065-T, Biotium; Fremont, CA, USA) were added upon medium change. Images were taken every 2 min over a time course of 30–60 min. LCI was performed at 37 °C using a Leica DMi8 microscope equipped with a climate chamber and an HC PL APO x63/1.40 oil objective. The Leica LAS X software was used to process the acquired images. Cells were regarded as oligomer positive at the time-point where multiple (> 5) oligomers were visible. Cells were counted manually by four different experimenters with at least 2 frames (30–60 cells per frame) per condition. The same microscope was used to image fixed cells. For confocal imaging, we used the Zeiss confocal laser scanning microscope LSM 900 with Airyscan 2 with Axio Observer 7. For 3D rendering, the deconvolution principles of LSM Plus were used. The Zeiss microscope is a service by the Imaging Unit of the Core Facility Hohenheim (CFH-IMG) of the University of Hohenheim, Stuttgart.

To analyse the co-localisation of NLRP10 and lipid droplets, we established a pipeline using the arivis Pro (Carl Zeiss AG, Oberkochen, GER) in cooperation with Dr. Marc Welzer at the core facilities at the University of Hohenheim (CFH-IMG). Briefly, cells were separated by “Cellpose-based Segmenter” and lipid droplets and NLRP10 oligomers were detected using the “Blob Finder 2”. Co-localisation was quantified by overlapping both signals. Sphericity was calculated using the mesh-based surface area and volume of the segmented 3D object.

### Indirect immunofluorescence

For indirect immunofluorescence staining, cells were seeded onto glass cover-slips. After respective treatment, cells were washed with PBS and subsequently fixed with 4% paraformaldehyde (#0335.1, Carl Roth) in PBS for 10 min at room temperature. After washing the cells thrice, they were permeabilised using ice-cold 0.1% Triton X-100 (#3051.2, Carl Roth) in PBS for 15 min at room temperature, followed by blocking unspecific protein binding by 3% FCS in PBS for 60 min at room temperature. Antibodies [rat IgG2aκ anti-NLRP10 8H2 (available at Merck-Sigma-Aldrich, #MABC293; RRID:AB_3713361), rabbit IgG anti-TOMM20 (D8T4N, Cell Signaling Technologies, #42406; RRID:AB_2687663), Goat anti-Rat IgG (H + L) Highly Cross-Adsorbed Secondary Antibody, Alexa Fluor™ Plus 488 (#A48262TR, TFS; RRID:AB_2896331), Goat anti-Rabbit IgG (H + L) Highly Cross-Adsorbed Secondary Antibody, Alexa Fluor™ 546 (#A-11035, TFS; RRID:AB_143051)] were applied for 2 h at room temperature and in a humidity chamber. Cells were embedded in polyvinyl alcohol [~3 mM Mowiol® 4–88 (Carl Roth), glycerol (20%), 0.1 M Tris-HCl (pH 8.5)] containing 10 µg/ml bis-Benzimid H 33258 (Hoechst 33258, Merck). To stain lipid droplets, cells were incubated with 1:1000 LipidSpot™ 610 for 10 min and mounted in Fluoromount-G™ (#00-4958-02, TFS).

### Protein expression and purification

The coding sequence of human NLRP10 (aa 4–655), Walker A mutant human NLRP10 (aa 4–655 K179A) or human NLRP10ΔHD2–tail (aa 4–482) was PCR-amplified and inserted into the pACEBac1 acceptor vector, carrying an N-terminal maltose binding protein (MBP)-tev affinity purification tag. All constructs were expressed in *Sf9* insect cells using the baculovirus expression system. Cells were lysed in pre-chilled lysis buffer (wt aa 4–655) and mutant: 25 mM Tris pH 7.5, 300 mM NaCl, 5 mM ß-ME, 5% glycerol; 4–482: 20 mM Tris pH 7.8, 150 mM NaCl, 5 mM ß-ME, 0.5 mM ADP, 10 mM MgCl_2_ supplemented with DNase (30 µM) and PMSF (1 mM) and subsequently sonicated. The cleared lysate was subjected to MBP affinity chromatography using a MBPtrap column (GE Healthcare, Munich, GER), equilibrated with lysis buffer. The protein was eluted in SEC buffer supplemented with 10 mM maltose. The protein was subsequently subjected to gel filtration chromatography using a Superose 6 Increase 10/300 GL or Superdex 200 Increase 10/300 GL column (GE Healthcare) in SEC buffer; wt (aa 4–655) and mutant: 25 mM Tris pH 7.5, 300 mM NaCl, 5 mM ß-ME, 5% glycerol; 4–482: 20 mM HEPES pH 7.8, 150 mM NaCl, 1 mM TCEP, 0.5 mM ADP, 10 mM MgCl2, 150 mM l-arginine. Protein quality was analysed by SDS-PAGE, and target-containing fractions were pooled, concentrated and snap-frozen in liquid nitrogen for further analysis.

### Negative stain EM

Negative stain electron microscopy was employed to evaluate protein sample quality, focusing on aggregation, heterogeneity and overall integrity. Carbon-coated copper grids were glow-discharged and then incubated for 1 min with 5 µl of the target protein sample. Excess sample was removed with blotting paper. The grid was washed by sequentially immersing it in three individual 20 µl drops of the corresponding purification buffer. Each time, residual liquid was removed. The grid was stained with 2% uranyl acetate for 30 sec, after which the stain was carefully blotted off, and the grid was air-dried. Imaging was carried out on a JEOL JEM-2200FS TEM operating at 200 kV and equipped with a CMOS camera (TemCam-F416). Micrographs were collected at 30k and 60k magnification.

### Ion-paired reverse-phase HPLC

Ion-paired reverse-phase HPLC was used to analyse the amount of nucleotide released upon ATP hydrolysis reactions. Nucleotides were separated using a Chromolith Performance RP-18 endcapped 100–4.6 HPLC column and the corresponding guard cartridge Chromolith RP-18 (Merck, Darmstadt, GER). The measurement was performed using an Agilent 1260 HPLC System (Agilent Technologies, Inc., Santa Clara, CA, USA). The mobile phase was composed of: 10 mM tetrabutylammonium bromide, 30 mM K_2_HPO_4_, 70 mM KH_2_PO_4_, 0.2 mM sodium azide, 4% acetonitrile, pH 7.5. The analysed protein samples were diluted to a final concentration of 3 μM in SEC buffer and were incubated with 100 μM ATP (Jena Bioscience, Jena, GER) at 25 °C. The samples were incubated in glass vials (Waters Corporation, Milford, MA, USA). In total, 10 μl aliquots were taken every 10 min with 1 min delay time. The HPLC measurement was performed at 25 °C at a flow rate of 1 ml/min, and the absorbance was continuously detected at 280 and 254 nm, respectively. In addition, a standard measurement for single nucleotides was performed. Peak integrals were determined with the appropriate analysis software and normalised to the standard measurement. The shown hydrolysis data refers to *N* = 3 or *N* = 2.

### Immunoblot analysis

Proteins were separated by SDS-PAGE and transferred to nitrocellulose membranes (Amersham™ Protran®). Proteins were detected by using mouse IgG1κ anti-GFP antibody (Merck-Roche, #11814460001; RRID:AB_390913), rat IgG2aκ anti-NLRP10 8H2 (available at Merck-Sigma-Aldrich, #MABC293; RRID:AB_3713361), mouse anti-GAPDH (#sc-47724, Santa Cruz Biotechnology; Dallas, TX, USA; RRID:AB_627678), HRP-conjugated goat anti-mouse and rabbit IgG antibodies (#170-6516; RRID:AB_11125547 and #170-6515; RRID:AB_11125142) and using the Clarity Western ECL Substrate (Bio-Rad Laboratories; Hercules, CA, USA, #1705061). GAPDH served as a loading control and was always detected on the same membrane as the main signal. Signals were recorded on an electronic camera system (Vilbert Fusion FX).

### Measurement of cytokines

IL-18 was measured in the supernatant of cells using the human total IL-18 DuoSet ELISA (#DY318, Biotechne; Minneapolis, MN, USA).

### Densitometric analysis

To analyse the densitometry of signals, we used the open-source software Fiji (RRID:SCR_002285) (Schindelin et al, 2012) and normalised to GAPDH signal as loading control and corrected by the mean intensity of each membrane.

### Protein sequence analysis

To predict protein parameters including pI, amino acid composition and charge, we used the Expasy ProtParam online tool operated by the SIB Swiss Institute of Bioinformatics (Wilkins et al, 1999). Conservation level was determined using standard alignment parameters of the Jalview software (RRID:SCR_006459) developed in Geoff Barton’s Group, Division of Computational Biology, School of Live Sciences at the University of Dundee, Scotland, UK (Waterhouse et al, 2009). For lysine ubiquitination prediction, we used the Rapid UBIquitination detection (RUBI) version 1 tool using 1% and 5% false positive rate by the Department of Biomedical Sciences, University of Padova (Walsh et al, 2014).

### Protein structure prediction

AlphaFold models AF-Q86W26-F1-model_v6 and AF-Q8CCN1-F1-model_v6 were used, and models were visualised using the UCSF ChimeraX software (RRID:SCR_015872) version 1.9 (2024-12-11) (Meng et al, 2023; Pettersen et al, 2021; Goddard et al, 2018).

### Data visualisation and analysis

Experimental data were visualised using GraphPad Prism 7 software (RRID:SCR_002798) and represent mean ± SEM or mean ± SD, “*N*” always refers to biological replicates. Statistics were performed using the integrated statistic tools. Multiple groups were compared using one-way ANOVA or Kruskal–Wallis test with indicated post-hoc, two groups were compared using Mann–Whitney *U* test. Vector graphics were generated with Inkscape (v1.4; 86a8ad7, 2024-10-11; RRID:SCR_014479).

## Supplementary information

Appendix

Peer Review File

Source data Fig. 1

Source data Fig. 2

Source data Fig. 3

Source data Fig. 4

Figure EV1 Source Data

Figure EV2 Source Data

Figure EV3 Source Data

Figure EV4 Source Data

Figure EV5 Source Data

Figure EV6 Source Data

Expanded View Figures

## Data Availability

This study includes no primary datasets deposited in external repositories. The source data of this paper are collected in the following database record: biostudies:S-SCDT-10_1038-S44319-026-00839-9.
