# Supplementary material for: C-terminal lysine residues localise NLRP10 at lipid droplets and govern NLRP10 oligomer formation
Source: EMBO Rep. 2026 Jun 26;27(15):4332–56. doi: 10.1038/s44319-026-00839-9 (PMC13458767; doi:10.1038/s44319-026-00839-9)
Supplement: Supplementary file 1 [file 44319_2026_839_MOESM13_ESM.pdf]

Expanded View Figures

Figure EV1. HeLa and HaCaT cells respond with *m*-3M3FBS-dependent NLRP10 oligomerisation without co-localisation with mitochondrial marker TOMM20.

(**A**) Stable HeLa cells show expression of eGFP-NLRP10 only in response to Dox. The upper panel shows immunoblot analysis with antibodies directed against GFP and GAPDH as a loading control; the lower panel shows micrographs of cells treated with or without Dox. (**B**) Live-cell imaging micrographs of eGFP-NLRP10 (white) and mts-mCherry (red) expressing HeLa cells treated with 85 µM *m*-3M3FBS or vehicle DMSO. (**C**) Micrographs of eGFP-NLRP10 (green) expressing HeLa cells treated with 85 µM *m*-3M3FBS stained with an antibody directed against TOMM20. Panel 3 shows one plane and panel 4 the deconvoluted z-stack. (**D**) Immunoblot and micrographs of HaCaT and NLRP10-knockout HaCaT (N10ko) cells stained with an antibody directed against NLRP10 and treated with 10 µg/ml poly(I:C) or 85 µM *m*-3M3FBS. (**E**) Quantification of the IL-18 release represented as mean ± SD of HaCaT cells treated with 10 µg/ml poly(I:C) or 85 µM *m*-3M3FBS (0 min, 10 min and 20 min). Nuclei were stained with HOECHST dye (**A**, **C**, **D**). Scale bar: 20 µm or 10 µm (**B**, inset). $N = 3$–4, each measured in duplicates (**E**). Source data are available online for this figure.

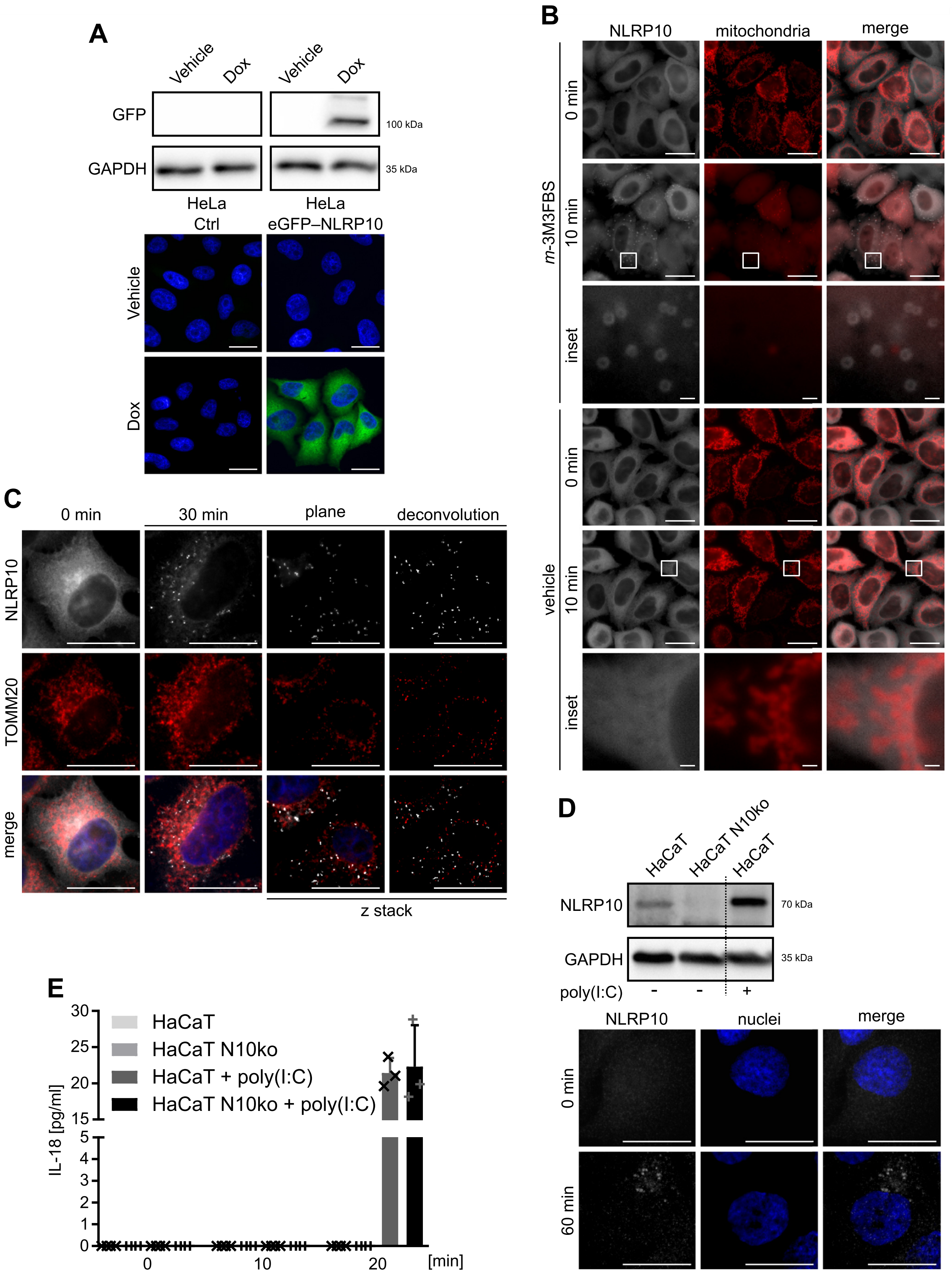
A
Vehicle
Dox
Vehicle
Dox
GFP
100 kDa
GAPDH
35 kDa
HeLa Ctrl
HeLa eGFP–NLRP10
Vehicle
Dox
B
NLRP10
mitochondria
merge
m-3M3FBS
0 min
10 min
inset
vehicle
0 min
10 min
inset
C
0 min
30 min
plane
deconvolution
NLRP10
TOMM20
merge
z stack
D
HaCaT
HaCaT N10ko
HaCaT
NLRP10
70 kDa
GAPDH
35 kDa
poly(I:C)
-
-
+
NLRP10
nuclei
merge
0 min
60 min
E
HaCaT
HaCaT N10ko
HaCaT + poly(I:C)
HaCaT N10ko + poly(I:C)
IL-18 [pg/ml]
30
25
20
15
5
4
3
2
1
0
0
10
20
[min]

 © The Author(s)

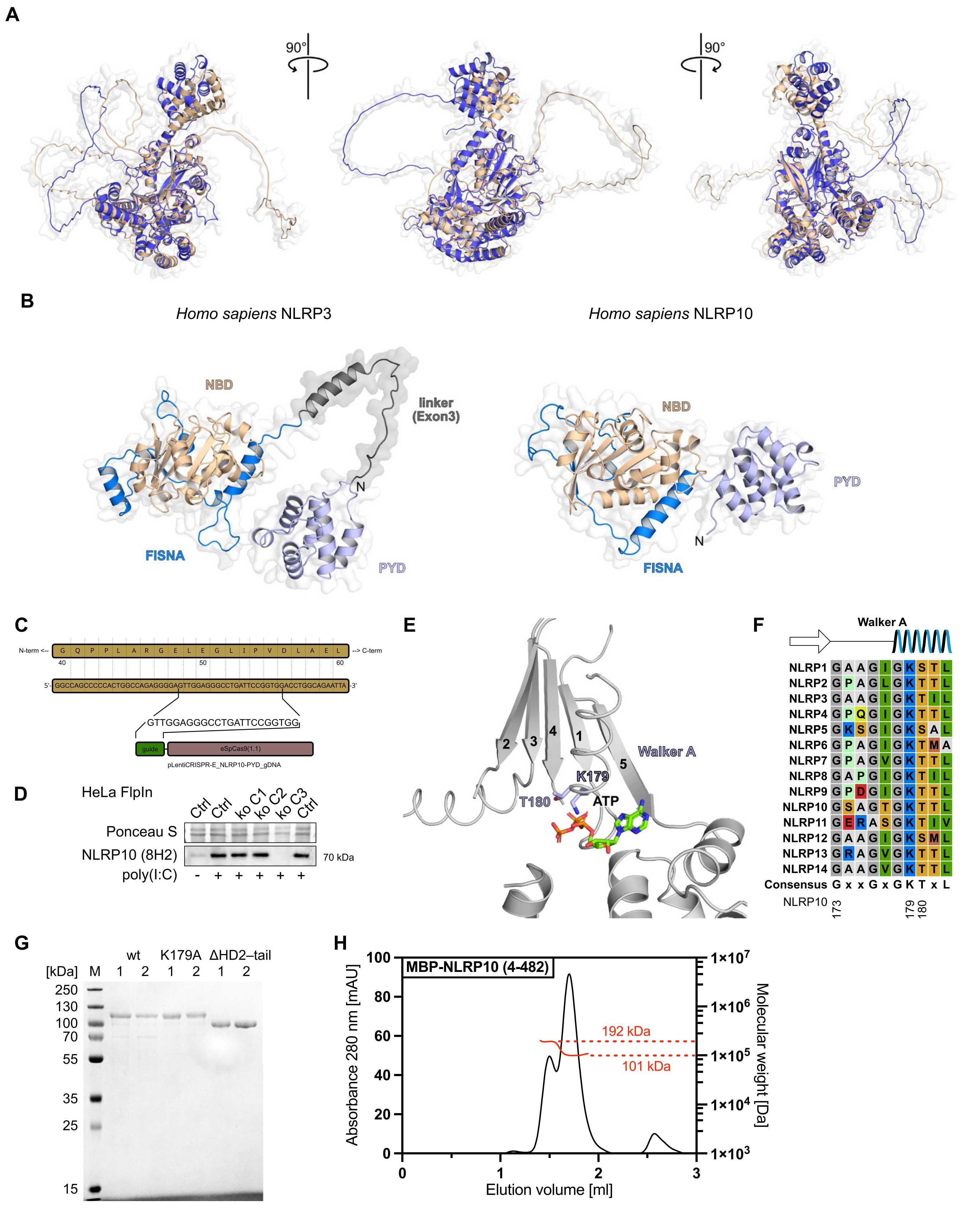
A
90°
90°
B
Homo sapiens NLRP3
Homo sapiens NLRP10
NBD
linker
(Exon3)
N
FISNA
PYD
NBD
PYD
N
FISNA
C
N-term <-- G Q P P L A R G E L E G L I P V D L A E L --> C-term
40
50
60
5'- GGCCAGCCCCCACTGGCCAGAGGGGAGTTGGAGGGCCTGATTCCGGTGGACCTGGCAGAATTA -3'
GTTGGAGGGCCTGATTCCGGTGG
guide
eSpCas9(1.1)
pLentiCRISPR-E_NLRP10-PYD_gDNA
D
HeLa FlpIn
Ctrl
Ctrl
ko C1
ko C2
ko C3
Ctrl
Ponceau S
NLRP10 (8H2)
70 kDa
poly(I:C)
- + + + + +
E
2
3
4
1
5
Walker A
K179
T180
ATP
F
Walker A
NLRP1 G A A G I G K S T L
NLRP2 G P A G L G K T T L
NLRP3 G A A G I G K T I L
NLRP4 G P Q G I G K T T L
NLRP5 G K S G I G K S A L
NLRP6 G P A G I G K T M A
NLRP7 G P A G V G K T T L
NLRP8 G A P G I G K T I L
NLRP9 G P D G I G K T T L
NLRP10 G S A G T G K T T L
NLRP11 G E R A S G K T I V
NLRP12 G A A G I G K S M L
NLRP13 G R A G V G K T T L
NLRP14 G A A G V G K T T L
Consensus G x x G x G K T x L
NLRP10
173
179
180
G
wt
K179A
ΔHD2–tail
[kDa]
M
1
2
1
2
1
2
250
130
100
70
55
35
25
15
H
MBP-NLRP10 (4-482)
192 kDa
101 kDa
Absorbance 280 nm [mAU]
100
80
60
40
20
0
Molecular weight [Da]
1×10⁷
1×10⁶
1×10⁵
1×10⁴
1×10³
0
1
2
3
Elution volume [ml]

Figure EV2. *Homo sapiens* and *Mus musculus* NLRP10 show high similarity with a different PYD-NACHT linker compared to NLRP3.

(**A**) Superpositioned AlphaFold models of human (blue) and mouse (beige) NLRP10 at three different angles. (**B**) The linker between the PYD and NACHT is encoded by an additional Exon in NLRP3, which results in a long stretch (grey), which is absent in NLRP10. (**C**) Schematics of the gDNA design to target the DNA anti-sense strand of the NLRP10 PYD. (**D**) Immunoblot analysis of HeLa FlpIn cells transduced with virions produced in Lenti-X 293T cells transfected with the pLentiCRISPR plasmid to target NLRP10 knockout. NLRP10 expression was enhanced using 10 µg/ml poly(I:C). (**E**) Cartoon of the nucleotide-binding site of NLRP10 modelled with AlphaFold3. The location of ATP refers to the model of NLRP3 (PDB: 7PZC). The Walker A motif is indicated with two catalytically active residues. (**F**) Sequence alignment of all NLRP family members with consensus for the Walker A motif. (**G**) SDS-PAGE of the fractions shown in Fig. 2G. (**H**) SEC-MALS measurements of MBP-NLRP10 (4–482) with determined molecular weights for the concentration-dependent monomer-to-dimer transition. Source data are available online for this figure.

 © The Author(s)

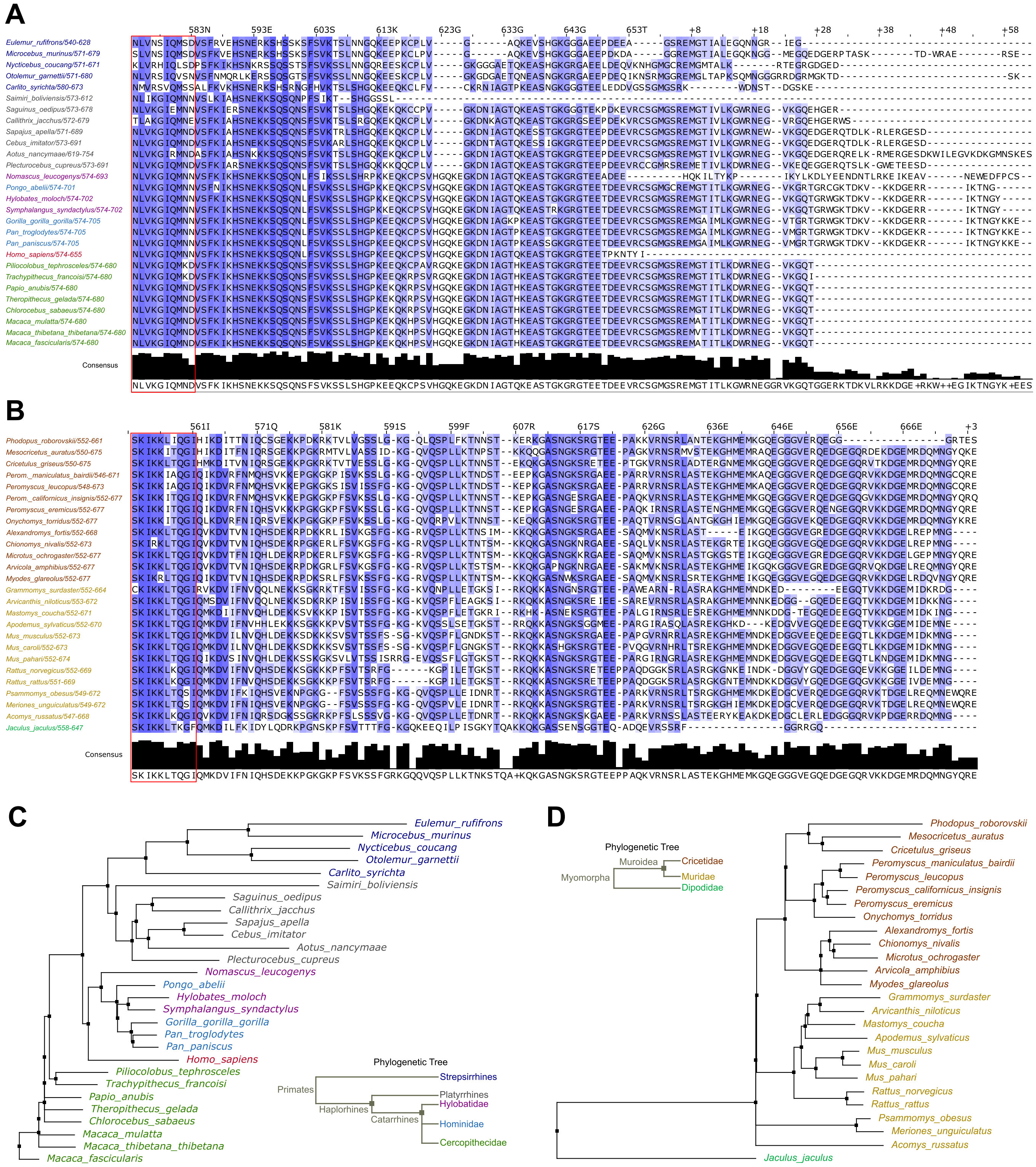

Figure EV3. Evolution of the NLRP10 basic tail: *Homo sapiens* NLRP10 shows divergence among Haplorhines.

(**A**, **B**) Multi-sequence alignment of primate NLRP10 tails and Myomorpha Nlrp10 tails. The last ten amino acids of the HD2 are shown in a red box. (**C**, **D**) Dendrogram according to NLRP10 sequences of primates or Nlrp10 sequences of Myomorpha. The dendrogram largely matches the general evolutionary distance of the species. The human NLRP10 (red) separates from all other Catarrhines (**C**). Among Myomorpha, only *Jaculus jaculus* separates, currently the only high-quality predicted sequence for Dipodidae (**D**). The colours of (**A**, **B**) match those of (**C**, **D**) and Appendix Tables Source data are available online for this figure.

© The Author(s)

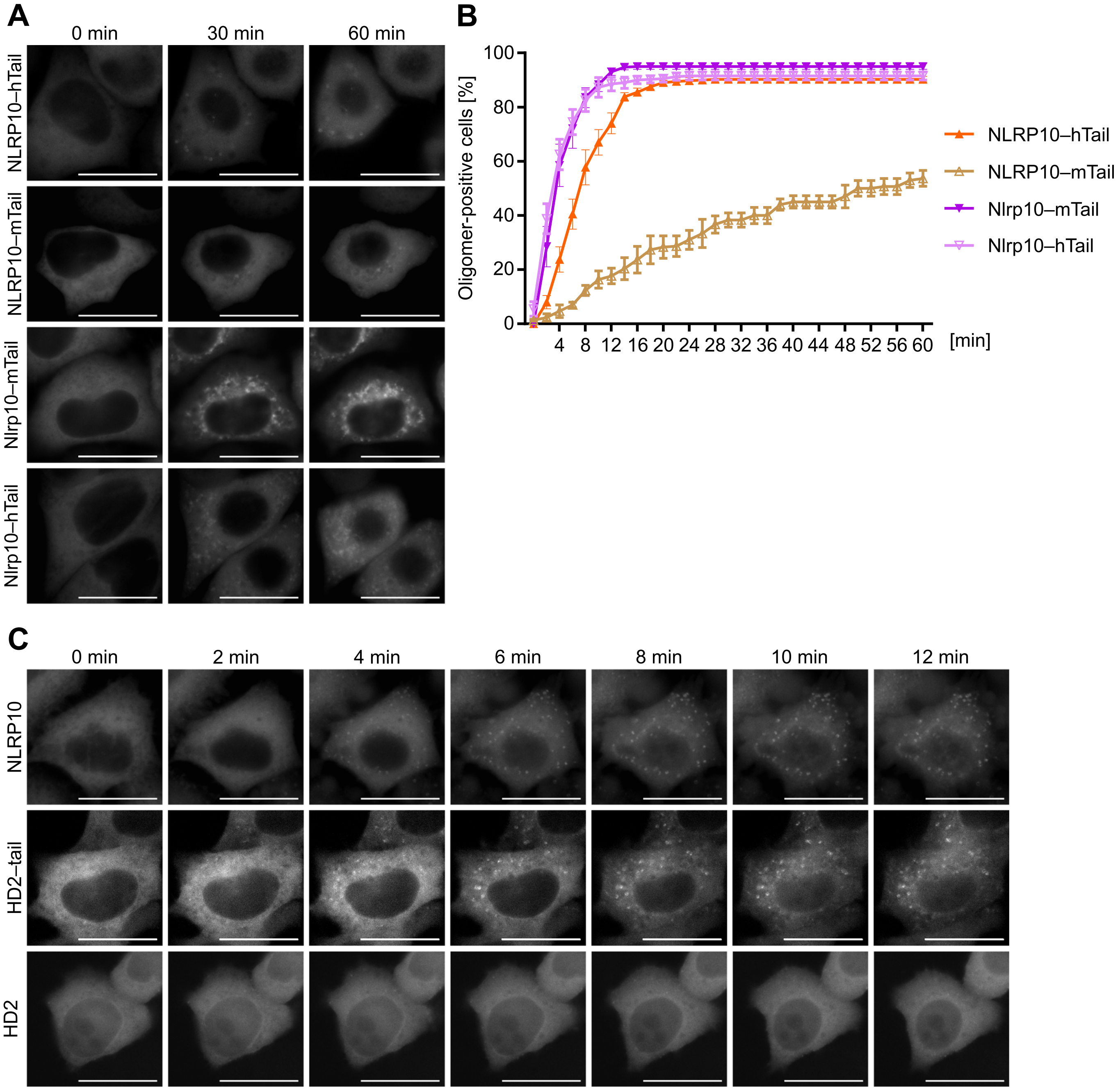

Figure EV4. Human and mouse chimeric NLRP10 can oligomerise, and the tail is sufficient for oligomerisation.

(**A**) Live-cell imaging micrographs of HeLa cells transiently transfected with chimeric NLRP10 and treated with 85 µM *m*-3M3FBS. (**B**) Quantification of the oligomer-positive cells from (**A**) represented as mean ± SEM. (**C**) Live-cell imaging micrographs of HeLa cells treated with 85 µM *m*-3M3FBS, which were transiently transfected with eGFP fused to the HD2 and HD2-tail of NLRP10 compared to NLRP10 (reused from Fig. 1A). Scale bar 20 µm. $N = 3$–4 (**B**). Source data are available online for this figure.

 © The Author(s)

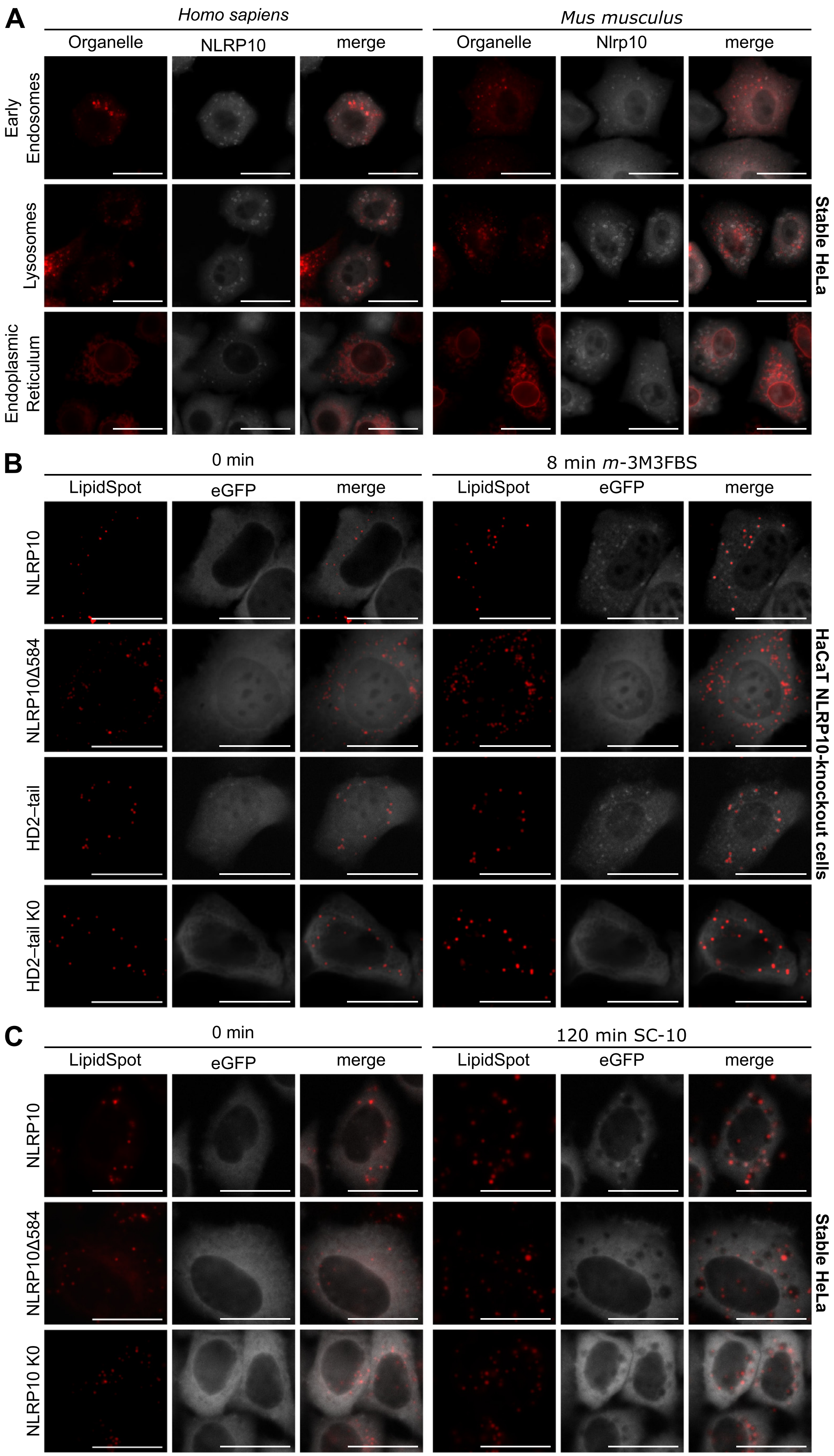
A
Homo sapiens
Mus musculus
Organelle
NLRP10
merge
Organelle
Nlrp10
merge
Early Endosomes
Lysosomes
Endoplasmic Reticulum
Stable HeLa
B
0 min
8 min m-3M3FBS
LipidSpot
eGFP
merge
LipidSpot
eGFP
merge
NLRP10
NLRP10Δ584
HD2-tail
HD2-tail KO
HaCaT NLRP10-knockout cells
C
0 min
120 min SC-10
LipidSpot
eGFP
merge
LipidSpot
eGFP
merge
NLRP10
NLRP10Δ584
NLRP10 KO
Stable HeLa

Figure EV5. Human and mouse NLRP10 partially co-localised with early endosomes and lysosomes but not with the endoplasmic reticulum.

(**A**) Live-cell imaging micrographs (30 min time-point) of HeLa cells expressing human NLRP10 (left) or mouse Nlrp10 (right) treated with 85 µM *m*-3M3FBS. (**B**) Micrographs of NLRP10ko HaCaT cells transiently transfected with eGFP-NLRP10, eGFP-NLRP10Δ584, eGFP-HD2–tail and eGFP-HD2–tail KO and subsequently treated with 85 µM *m*-3M3FBS. (**C**) Live-cell imaging micrographs of HeLa cells expressing NLRP10 (reused from Fig. 1B), NLRP10Δ584, or the KO mutant (white) treated with 100 µM SC-10 and stained with LipidSpot™ 610 (red). Scale bar 20 µm.Source data are available online for this figure.

 © The Author(s)

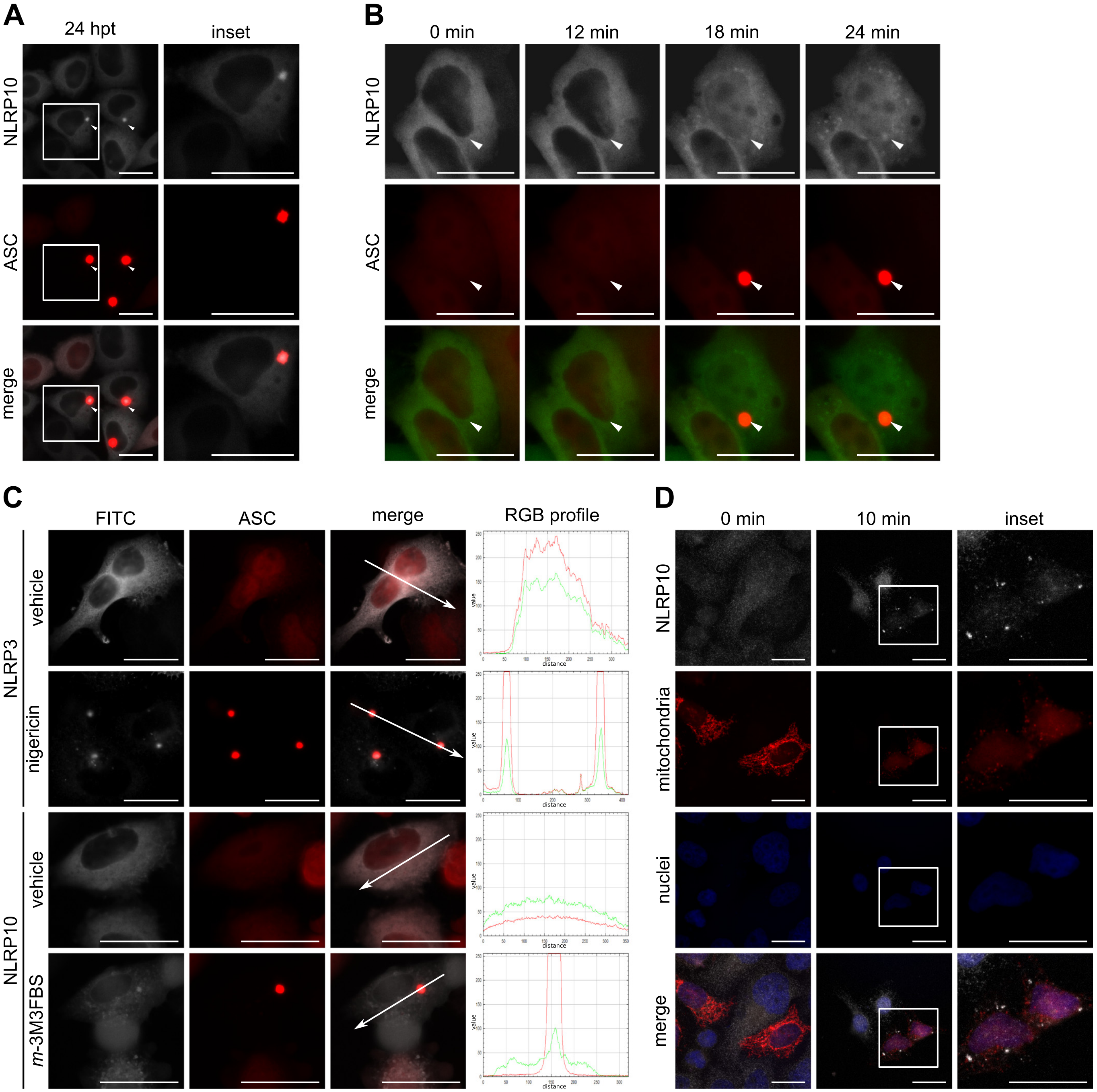

Figure EV6. NLRP10/ASC specks are different to NLRP3/ASC specks.

(**A**) Live-cell imaging micrographs of stable HeLa cells expressing eGFP-NLRP10 transiently transfected with ASC-RFP (24 h post-transfection). (**B**) Live-cell imaging micrographs of stable HeLa cells expressing ASC-RFP under an CMVd1 promoter transiently transfected with eGFP-NLRP10 (24 hpt) and treated with 85 µM *m*-3M3FBS. White arrows indicate specks. (**C**) Micrographs of stable HeLa cells expressing ASC-RFP under an CMVd1 promoter transiently transfected with myc-NLRP3 or eGFP-NLRP10 (24 hpt) and treated with 10 µM nigericin (120 min) or 85 µM *m*-3M3FBS (12 min), respectively. RGB profile of white lines from the third panel showing ASC signal (red) and NLRP3 or NLRP10 signal (green). (**D**) Micrographs of HeLa cells transiently transfected with mts-mCherry (red) and stained with an antibody (clone 8H2) directed against NLRP10 (white) treated with vehicle or 85 µM *m*-3M3FBS, nuclei were stained with HOECHST dye. Scale bar 20 µm.Source data are available online for this figure.

© The Author(s)
