# Supplementary material for: C-terminal lysine residues localise NLRP10 at lipid droplets and govern NLRP10 oligomer formation
Source: EMBO Rep. 2026 Jun 26;27(15):4332–56. doi: 10.1038/s44319-026-00839-9 (PMC13458767; doi:10.1038/s44319-026-00839-9)
Supplement: Supplementary file 2 [file 44319_2026_839_MOESM1_ESM.pdf]

Appendix for

C-terminal lysine residues localise NLRP10 at lipid droplets and govern NLRP10 oligomer formation

The PDF file includes:

Appendix Table S1 – page 2

Appendix Table S2 – page 3

Appendix Table S3 – page 4

Appendix Tables

Appendix Table S1: List of NLRP10 reference sequences of primates including total length and length of expected tail with expected pI according to human protein model and alignment mapping the last 10 amino acids of the *Homo sapiens* NLRP10 NACHT domain (NLVKGIQMNN).

Reference	Species	Length	Tail (aa)	pI	Comment
XP_003920428.3	*Saimiri boliviensis*	612	30	10.00	Shortest tail
NP_001378887.1	*Homo sapiens*	655	72	9.52	
XP_069325622.1	*Eulemur rufifrons*	628	79	5.91	Low tail pI
XP_008060833.2	*Carlito syrichta*	673	84	9.01	Starts with T
XP_053415859.1	*Nycticebus coucang*	671	91	9.08	
KAK2100778.1	*Saguinus oedipus*	678	96	9.55	
XP_008005997.2	*Chlorocebus sabaeus*	680	97	9.25	
XP_015290797.3	*Macaca fascicularis*	680	97	9.47	
XP_001098361.3	*Macaca mulatta*	680	97	9.24	
XP_050615200.1	*Macaca thibetana thibetana*	680	97	8.81	
XP_021781824.2	*Papio anubis*	680	97	9.63	
XP_023045669.1	*Piliocolobus tephrosceles*	680	97	8.52	
XP_033061140.1	*Trachypithecus francoisi*	680	97	9.30	
XP_025211582.1	*Theropithecus gelada*	680	97	8.93	
XP_002755056.3	*Callithrix jacchus*	679	98	9.83	
XP_012639395.1	*Microcebus murinus*	679	99	5.95	Low tail pI
XP_003781555.1	*Otolemur garnettii*	680	100	9.66	
XP_017355843.1	*Cebus imitator*	691	108	8.96	
KAL0629402.1	*Plecturocebus cupreus*	691	109	7.86	
XP_032096543.1	*Sapajus apella*	689	109	8.96	
XP_030685543.1	*Nomascus leucogenys*	693	110	8.49	
XP_012311817.2	*Aotus nancymaae*	754	117	9.39	Atypical PYD
XP_002822118.2	*Pongo abelii*	701	118	9.76	
XP_032021750.1	*Hylobates moloch*	702	119	9.84	
XP_055140623.1	*Symphalangus syndactylus*	702	119	9.93	
XP_055212467.2	*Gorilla gorilla gorilla*	705	122	9.81	Longest tail
XP_003818292.1	*Pan paniscus*	705	122	9.73	Longest tail
XP_003313013.5	*Pan troglodytes*	705	122	9.73	Longest tail

Appendix Table S2: List of Nlrp10 reference sequences of myomorpha including total length and length of expected tail with expected pI according to mouse protein model and alignment mapping the last 10 amino acids of the *Mus musculus* Nlrp10 NACHT domain (SKIKKLTQGI).

Reference	Species	Length	Tail (aa)	pI	Comment
XP_012805509.2	*Jaculus jaculus*	647	80	9.85	Shortest tail
XP_051048548.1	*Phodopus roborovskii*	661	100	10.0	
XP_028623966.1	*Grammomys surdaster*	664	103	9.88	
XP_050019272.1	*Alexandromys fortis*	668	107	9.93	
NP_001099761.1	*Rattus norvegicus*	669	108	9.43	
XP_052011198.1	*Apodemus sylvaticus*	670	109	9.46	
XP_032751552.1	*Rattus rattus*	669	109	9.55	
XP_076796644.1	*Arvicanthis niloticus*	672	110	9.62	
XP_031243649.1	*Mastomys coucha*	671	110	9.13	
XP_051005138.1	*Acomys russatus*	668	112	9.79	
XP_057635579.1	*Chionomys nivalis*	673	112	9.82	
XP_021024703.1	*Mus caroli*	673	112	9.13	
NP_780741.1	*Mus musculus*	673	112	8.82	Lowest pI
XP_021074826.1	*Mus pahari*	674	113	9.22	
XP_021517635.2	*Meriones unguiculatus*	672	114	9.52	
XP_055463457.1	*Psammomys obesus*	672	114	9.63	
XP_038199953.1	*Arvicola amphibius*	677	116	9.94	
XP_035297287.1	*Cricetulus griseus*	675	116	9.43	
XP_005075823.1	*Mesocricetus auratus*	675	116	9.43	
XP_005351132.1	*Microtus ochrogaster*	677	116	9.73	
XP_048316419.1	*Myodes glareolus*	677	116	9.77	
XP_036033111.1	*Onychomys torridus*	677	116	9.99	
XP_052619135.1	*Peromyscus californicus insignis*	677	116	9.65	
XP_059107555.1	*Peromyscus eremicus*	677	116	9.88	
XP_028750420.1	*Peromyscus leucopus*	673	116	9.76	
XP_006978619.1	*Peromyscus maniculatus bairdii*	671	116	9.63	

Appendix Table S3**:** List of oligonucleotide primers used in this study.

Name	Sequence (5'->3')	Usage
NLRP10_F	CAAGGGATCCATGGCCATGGCC	N-term.
NLRP10_R	TAGACTCGAGTTATATGTAAGTATTTTTTGGTG	C-term.
NLRP10_1-583_R	GTGGTCGACGATATCTTAATTGTTCATCTGAATACC	Δ584
NLRP10_1-482_R	GTGGTCGACGATATCTTACTCTTTCACCAGGTAAGACATGG	Δ483
NLRP10_ΔPYD_F	GCGCGGATCCTACAGAGAAGTATACCGAGAGCATGTG	ΔPYD
Nlrp10_BglII_F	GAAGATCTATGGCCTTGGCACGGGCCAA	N-term.
Nlrp10_XhoI_R	GATCCTCGAGCTACCCATTCATC	C-term.
Nlrp10dPYD_BglII_F	GAAGATCTATGGAGCTTGTAGACTACCTCA	ΔPYD
Nlrp10Δtail_XhoI_R	GATCCTCGAGTTAGATACCTTGTGTGAGCTTCT	Δ562
NLRP10_nsc_XhoI	TAGACTCGAGATTGTTCATCTGAATACC	chimeras
Nlrp10Δtail_nsc_XhoI_R	GATCCTCGAGGATACCTTGTGTGAGCTTCT	chimeras
XhoI_hTail_F	GATCCTCGAGGTATCATTCAAGATAAAACATTC	chimeras
hTail_ApaI_R	GTGGGCCCTTATATGTAAGTATTTTTTGGTG	chimeras
XhoI_mTail_R	GATCCTCGAGCAGATGAAAGATGTCATTCTC	chimeras
mTail_ApaI_R	CTGGGCCCCTACCCATTCATCTTATCTATCATC	chimeras
