# Supplementary material for: C-terminal lysine residues localise NLRP10 at lipid droplets and govern NLRP10 oligomer formation
Source: EMBO Rep. 2026 Jun 26;27(15):4332–56. doi: 10.1038/s44319-026-00839-9 (PMC13458767; doi:10.1038/s44319-026-00839-9)
Supplement: Supplementary file 3 [file 44319_2026_839_MOESM2_ESM.pdf]

C-terminal lysine residues localise NLRP10 at lipid droplets and govern NLRP10 oligomer formation

Timo-Daniel Voss, Christoph Winterberg, Adrian Beck, Clarissa Gottschild, Leonie Mueller, Selina Enayat, Matthias Geyer, and Thomas Kufer

Corresponding author(s): Timo-Daniel Voss (timodaniel.voss@uni-hohenheim.de)

Review Timeline:

Submission Date:	12th Dec 25
Editorial Decision:	19th Jan 26
Revision Received:	16th Apr 26
Editorial Decision:	11th May 26
Revision Received:	3rd Jun 26
Accepted:	5th Jun 26

Editor: Achim Breiling

Transaction Report:

(Note: With the exception of the correction of typographical or spelling errors that could be a source of ambiguity, letters and reports are not edited. Depending on transfer agreements, referee reports obtained elsewhere may or may not be included in this compilation. Referee reports are anonymous unless the Referee chooses to sign their reports.)

Dear Dr. Voss,

Thank you for the transfer of your manuscript to EMBO reports. I have now received reports from the three referees that were asked to evaluate your study, which can be found at the end of this email. As you will see, the referees have several comments, concerns, and suggestions, indicating that a major revision of the manuscript is necessary to allow publication of the study in EMBO reports. As the reports are below, and all points need to be addressed, I will not detail them here.

Given the constructive referee comments, I would thus like to invite you to revise your manuscript with the understanding that the concerns of the referees must be addressed in the revised manuscript and in a detailed point-by-point response. Acceptance of your manuscript will depend on a positive outcome of a second round of review. It is EMBO reports policy to allow a single round of revision only and acceptance of the manuscript will therefore depend on the completeness of your responses included in the next, final version of the manuscript.

Revised manuscripts should be submitted within three months of a request for revision. Please contact me to discuss the revision (also by video chat) if you have questions or comments regarding the revision, or should you need additional time.

When submitting your revised manuscript, please also carefully review the instructions that follow below.

PLEASE NOTE THAT upon resubmission revised manuscripts are subjected to an initial quality control prior to exposition to re-review. Upon failure in the initial quality control, the manuscripts are sent back to the authors, which may lead to delays. Frequent reasons for such a failure are the lack of the data availability section (please see below) and the presence of statistics based on n=2 (the authors are then asked to present scatter plots or provide more data points).

When submitting your revised manuscript, we will require:

1) a .docx formatted version of the final manuscript text (including legends for main figures, EV figures and tables), but without the figures included. Figure legends should be compiled at the end of the manuscript text.

2) individual production quality figure files as .eps, .tif, .jpg (one file per figure), of main figures and EV figures. Please upload these as separate, individual files upon re-submission.

The Expanded View format, which will be displayed in the main HTML of the paper in a collapsible format, has replaced the Supplementary information. You can submit up to 6 images as Expanded View. Please follow the nomenclature Figure EV1, Figure EV2 etc. The figure legend for these should be included in the main manuscript document file in a section called Expanded View Figure Legends after the main Figure Legends section. Additional Supplementary material should be supplied as a single pdf file labeled Appendix. The Appendix should have page numbers and needs to include a table of content on the first page (with page numbers) and legends for all content. Please follow the nomenclature Appendix Figure Sx, Appendix Table Sx etc. throughout the text, and also label the figures and tables according to this nomenclature.

For more details, please refer to our guide to authors:
https://link.springer.com/journal/44319/submission-guidelines#cms-Revised-submissions

Please consult our guide for figure preparation:
https://media.springernature.com/original/springer-cms/rest/v1/content/27825798/data/v1

3) a .docx formatted letter INCLUDING the reviewers' reports and your detailed point-by-point responses to their comments. As part of the EMBO Press transparent editorial process, the point-by-point response is part of the Review Process File (RPF), which will be published alongside your paper.

4) a complete author checklist, which you can download from our author guidelines (https://link.springer.com/journal/44319/submission-guidelines#cms-Revised-submissions). Please insert page numbers in the checklist to indicate where the requested information can be found in the manuscript. The completed author checklist will also be part of the RPF.

Please also follow our guidelines for the use of living organisms, and the respective reporting guidelines:
https://link.springer.com/partners/embo-press/editorial-policies#Animal%20and%20human%20studies

5) that primary datasets produced in this study (e.g. RNA-seq, ChIP-seq, structural and array data) are deposited in an appropriate public database. If no primary datasets have been deposited, please also state this in a dedicated section (e.g. 'No primary datasets have been generated and deposited'), see below.

See also: https://link.springer.com/partners/embo-press/editorial-policies#Data%20deposition

Please remember to provide a reviewer password if the datasets are not yet public.

The accession numbers and database should be listed in a formal "Data Availability" section that follows the model below. This is now mandatory (like the COI statement). Please note that the Data Availability Section is restricted to new primary data that are part of this study. This section is mandatory. As indicated above, if no primary datasets have been deposited, please state this in this section

Data availability

The datasets produced in this study are available in the following databases:

- RNA-Seq data: Gene Expression Omnibus GSE46843 (https://www.ncbi.nlm.nih.gov/geo/query/acc.cgi?acc=GSE46843)
- [data type]: [name of the resource] [accession number/identifier/doi] ([URL or identifiers.org/DATABASE:ACCESSION])

*** Note - All links should resolve to a page where the data can be accessed. ***

See also: https://link.springer.com/partners/embo-press/editorial-policies#Data%20availability%20statement

Moreover, I have these editorial requests:

6) We now request the publication of original source data with the aim of making primary data more accessible and transparent to the reader. You will receive a separate email with instructions for providing source data with your revised manuscript, including information how to upload and organize the files.

7) Our journal encourages inclusion of *data citations in the reference list* to directly cite datasets that were re-used and obtained from public databases. Data citations in the article text are distinct from normal bibliographical citations and should directly link to the database records from which the data can be accessed. In the main text, data citations are formatted as follows: "Data ref: Smith et al, 2001" or "Data ref: NCBI Sequence Read Archive PRJNA342805, 2017". In the Reference list, data citations must be labeled with "[DATASET]". A data reference must provide the database name, accession number/identifiers and a resolvable link to the landing page from which the data can be accessed at the end of the reference. Further instructions are available at: https://link.springer.com/journal/44319/submission-guidelines#cms-Reference-guidelines

8) Regarding data quantification and statistics, please make sure that the number "n" for how many independent experiments were performed, their nature (biological versus technical replicates), the bars and error bars (e.g. SEM, SD) and the test used to calculate p-values is indicated in the respective figure legends (also for EV and Appendix figures). Please also check that all the p-values are explained in the legend, and that these fit to those shown in the figure. Please provide statistical testing where applicable. Please avoid the phrase 'independent experiment' but clearly state if these were biological or technical replicates. Please also indicate (e.g. with n.s.) if testing was performed, but the differences are not significant. In case n=2, please show the data as separate datapoints without error bars and statistics. See also:
https://link.springer.com/journal/44319/submission-guidelines#cms-Figure-and-data-presentation

If n<5, please show single datapoints for diagrams.

9) Please add scale bars of similar style and thickness to all microscopic images, using clearly visible black or white bars (depending on the background). Please place these in the lower right corner of the images themselves. Please do not write on or near the bars in the image but define the size in the respective figure legend.

10) Please also note our reference format:
https://link.springer.com/journal/44319/submission-guidelines#cms-Reference-guidelines

11) We updated our journal's competing interests policy in January 2022 and request authors to consider both actual and perceived competing interests. Please name this section 'Disclosure and Competing Interests Statement' and put it after the Acknowledgements section.
See: https://link.springer.com/partners/embo-press/editorial-policies#Competing%20interest%20disclosures

12) We now use CRediT to specify the contributions of each author in the journal submission system. CRediT replaces the author contribution section. Please use the free text box to provide more detailed descriptions and do NOT provide your final manuscript text file with an author contributions section. See also our guide to authors (section 'Author contributions'):
https://link.springer.com/journal/44319/submission-guidelines#cms-Revised-submissions

13) All Materials and Methods need to be described in the main text using our 'Structured Methods' format, which is required for all research articles. According to this format, the Methods section should include a Reagents and Tools Table (listing key reagents, experimental models, software, and relevant equipment and including their sources and relevant identifiers), uploaded

as separate file, and a Methods section in which we encourage the authors to describe their methods using a step-by-step protocol format with bullet points, to facilitate the adoption of the methodologies across labs. More information on how to adhere to this format as well as downloadable templates (.doc) for the Reagents and Tools Table can be found in our author guidelines (section 'Structured Methods'):

https://link.springer.com/journal/44319/submission-guidelines#cms-Manuscript-organisation-and-formatting

Please add all primer information directly to the Reagents and Tools table.

14) Please order the manuscript sections like this, using only these names:
Title page - Abstract (max. 175 words) - Keywords (max. 5) - Introduction - Results - Discussion - Methods - Data availability section - Acknowledgements (please include here all the funding information) - Disclosure and Competing Interests Statement - References - Figure legends - Expanded View Figure legends

15) Please make sure that all the funding information is also entered into the online submission system and that it is complete and similar to the one in the acknowledgement section of the manuscript text file.

We would also welcome the submission of cover suggestions, or motifs to be used by our Graphics Illustrator in designing a cover.

As part of the EMBO publication's Transparent Editorial Process, EMBO reports publishes online a Review Process File (RPF) to accompany accepted manuscripts. This File will be published in conjunction with your paper and will include the referee reports, your point-by-point response and all pertinent correspondence relating to the manuscript.

You are able to opt out of this by letting the editorial office know (emboreports@embo.org). If you do opt out, the Review Process File link will point to the following statement: "No Review Process File is available with this article, as the authors have chosen not to make the review process public in this case."

I look forward to seeing a revised form of your manuscript when it is ready.

Yours sincerely,

Achim Breiling
Senior Editor
EMBO Reports

Referee #1:

Overview:
The authors show that human and murine NLRP10 are able to aggregate in response to m-3M3FBS and that this aggregation is dependent on the NLRP10 C-terminal tail. NLRP10 ATPase activity and PYD were shown not to be necessary for this behaviour while positively charged residues in the tail are important. Aggregation is shown to happen at lipid droplets and to colocalise with ASC specks. Lipid droplets are present prior to m-3M3FBS treatment and changes in lipid composition are suggested as a mechanism for recruitment.

The delineation of important NLRP10 domains, the importance of charged residues in the tail and the involvement of lipid droplets are all novel and of interest to the innate immunity field. The putative effect of mitochondrial damage on lipids may also be of wider interest. In addition, the authors dispute previous reports of localisation of NLRP10 to the mitochondria following m-3M3FBS treatment.
As discussed in the comments below, the paper would benefit from greater connectivity throughout, either providing a definitive model or more clearly defining the limits of the work. Some suggestions to individual experiments are also suggested.

General Comments:

• English language is generally good, with a few sentences that could use clarification. Some examples are highlighted in the specific comments.
• Line numbers in the merged version reset every page.
• Figure quality is generally good
• K179 is sometimes written as K197
• The word "fixated" is used instead of "fixed"
• The relationship between NLRP10 and ASC is unclear. Earlier in the paper (Line 22) it is suggested that "some NLRP10 aggregates recruited ASC" but later ASC recruitment is suggested to precede NLRP10 aggregation. The proposed connection

between NLRP10 and ASC should be clarified.
• It is unclear throughout and in the title whether NLRP10 is proposed to be oligomerising and/or clustering. Both words are used but to me these present significantly different situations. The proposed model and order of events should be clarified.
• The tail and lysine residues are described as "conserved" in places (Line 24: "Collectively, the highly conserved C-terminal tail of NLRP10 is necessary for protein...") but it is not always clear how relevant this conservation is. Conservation should be put in some kind of context - for example, showing that lysine residues are more highly conserved than other residues in the tail, or perhaps showing that the tail is more conserved than PYD, which is suggested to be less important compared to other NLRs. IDRs are notoriously difficult to align and residues do not necessarily need to show aligned conservation to be functionally important
• The ubiquitination aspect is predicted but not followed up or discussed - this could be removed to shorten and focus the manuscript

Specific Comments:
Abstract
• Line 9, spelling?: "Convers"

Introduction
• Line 4, additional "a": "(nucleotide-binding..."
• Line 27: "The structures of the..." The argument at the end of this paragraph is slightly unclear to me. Is it that orthologous proteins (including hs/mmNLRP10) can have different functions? There are different copy numbers of NLRP1, is the situation comparable to NLRP10?
• Line 36, missing word: "Further understanding of the role..."

Results
Oligomerisation of NLRP10 upon m-3M3FBS treatment is ASC- and ATP hydrolysis-2 independent
• Line 12/Figure 1: "We observed..."
o Could the authors supplement an image series that was not treated with m-3M3FBS to demonstrate that the loss of mCherry signal is not due to photobleaching?
o Alternatively, could the authors indicate in methods that the signal display is normalised to the first frame?
• Line 15/Figure 1: "Thereby..."
o Are these structures circular or more generally aggregated? Can roundness be quantified?
• Line 21/Figure 1B: "Upon m-3M3FBS treatment..."
o Is it possible to quantify the ASC specks in the same way as the NLRP10 aggregates in 1C?
o Alternatively, could the authors note that these FOVs are representative of the general cell population?
o Do the authors have an example of an NLRP10 aggregate forming followed by an ASC speck forming on top? This would be a more convincing demonstration of recruitment (Following the description of order of events in Discussion section/S6, I recommend removing or clarifying references to ASC recruitment)
• Line 25/Figure S1C/D: "As for..."
o Was S1C performed in the KO cells also?
o The relevance of the positive control in S1D is unclear to me
The possibility that NLRP10 releases IL-18 in a canonical manner is being tested: NLRP10 ASC Caspase-1 IL-18 processing
Poly(I:C) IL-18 release would not involve an inflammasome, it is unclear that these cells can undergo pyroptosis under these conditions
Cell type and time point for positive control are not listed
NLRP10 aggregation depends on its C-terminal tail region
• Line 36/Figure S2A: "Super-positioning..."
o Does this contribute to the discussion of the C-terminal region? Only the NACHT is shown to overlay here
• Line 9, spelling: "obtained for either the human or the..."
• Line 29: "Proteins were separated..."
o Could the authors supplement the SDS PAGE corresponding to the peaks? A pure sample should be used to assess ATPase activity but contaminants often copurify in the void
• Line 35: "As expected..."
o Can this significance be tested and quantified?
• I am not clear on the argument behind the recombinant protein work. Either main text or supplementary commentary might help:
o "if ATP binding, which is required for an active protein conformation..." Which peak (if any) correspond to the active conformation in this assay? WT and WA mutant have a similar SE profile and all three have the void peak.
o Void peaks - Are these PYD-driven filaments?
o Oligomers - NACHT-driven disks? Tail-driven oligomers?
o Monomerisation of truncated protein - Can the effects of removing HD2 vs removing tail be distinguished? Was a Δtail construct purified, alternatively a literature reference indicating that removal of HD2 does not affect oligomerisation of NLRs?
Evolutionarily conserved basic residues in the C-terminus of NLRP10 mediate oligomerisation
• Line 13: "NLRP10 seems to have underwent complex..."

o Could the authors clarify this sentence?
• Line 37: "Deletion of the human..."
o Could authors be more consistent in descriptive language here? The non-significant increase in Nlrp10 from Figure 3B is described as "an increase" while the non-significant decrease in Nlrp10 from Figure 3C is described as having no effect - I don't think this influences author conclusions but would improve clarity
• Line 34: "Mitigates" - Is this the correct word?
• Figure 3J:
o Was an equivalent of 3J done with Nlrp10?
o Did mutating non-basic residues have a similar effect?
The NLRP10 oligomers are located at lipid compartments
• Line 6: "We found that..."
o These have not been described as or shown to be ring-like up until this point. Is a supplementary figure missing?
Discussion
• Line 10: "This segment..."
o Could the authors clarify the connection between the NLRP10 tail and NLRP3 loop?
• Line 13, spelling : "Is not dependent..."
Methods
• Can the authors clarify the following lines:
o "9.3 g 0.5 M EDTA"
o "wt 4-655 and mutant"

Referee #2:

The work by Voss et al. elucidates the function of the C-terminus in the atypical NLR protein, NLRP10. A key finding is that the C-terminus mediates NLRP10 oligomerization and recruitment to lipid droplets upon activation. While this conclusion is compelling, the additional claim of species-specific regulation of protein stability (human vs. mouse) necessitates more mechanistic support.
The study is significant as it partially corroborates prior reports on NLRP10 inflammasome and clarifies how NLRP10 is activated by m-3m3FBS (PMID: 36941399, 36941400). The evidence for oligomerization and localization is convincing, having been demonstrated in multiple model cell lines, although some conclusions rely on overexpression systems. While mechanistic details remain sparse, in particular regarding the effects of m-3m3-FBS on NLRP10 or lipid droplets, this work lays a crucial foundation for future research into NLRP10. The distinctive features of NLRP10 compared to other NLRs are highlighted, making this work of clear interest to the community of pattern recognition receptor and inflammasome research.

Major points:
1. Figure 3 currently combines data on NLRP10 protein stability and oligomerization. It would be easier to interpret if Figure 3 focuses solely on NLRP10 stability, with the oligomerization data moved to other figures.
2. The use of only one activator (m-3M3FBS) limits the generalizability of the conclusions. The study would be strengthened by including one or more published NLRP10 activators (e.g., thapsigargin, SMBA1; PMIDs: 36941399, 36941400). Furthermore, experiments using known inhibitors such as cyclosporin A would be valuable to confirm the specificity of the activation pathway.
3. The study includes a comparison of a number of different NLRP10 constructs (deletions, mutations) as well as human and mouse protein. Given that expression levels could affect oligomerization of the protein, the authors need to include immunoblots or FACS analysis confirming equal expression of all proteins analyzed.

Minor points (all page and line numbers are for the merged PDF):
1. In general, typo errors are found throughout the manuscript. A thorough proofreading is recommended. For example, Page 18, Fig. 2E: NLRP10 should be italicized. Page 11, line 1: The first "m" should be consistently italicized for m-3m3FBS.
2. Page 2, line 5: The claim that "ASC nucleation and recruitment were different to other NLRPs" is not supported by the authors' data, as they did not directly examine other NLRPs in their models.
3. Fig A: Nlrp10 aggregation is not that well visible, please include a quantification of aggregation. Include also a well established marker for mitochondrial damage.
4. Page 2, lines 15-16 (Fig. 1A): the claim that circular structures are visible is not well supported. Please include insets showing this.
5. Page 4, line 32 and 33. The statement that "NLRP10 does not form classical inflammasomes" appears to contradict the data in Fig. 1B, which shows m-3M3FBS-induced ASC speck formation. This statement should be clarified.
In addition, the authors should define if each ASC speck co-localizes with NLRP10, and if NLRP10 activation is the cause for ASC formation.
6. Figure 1C: Does K179A induce ASC speck formation, and how does this compare to WT
7. Fig. 2F-G: How does the deletion of HD2 in other NLRs affect ATP hydrolysis activity? Is it similarly lost as for Nlrp10?
8. Fig. 4D: does 3M3-FBS induce droplet formation? and what percentage of NLRP10 speckles co-localize with lipid droplets
9. Page 6, line 2 - 3: The claim that Peak 2 contains monomers needs to be supported. The explanation for why the monomers lose ATPase activity is missing and needs to be elaborated.

10. Page 7, line 4 and 5: Given that tail deletion in mouse Nlrp10 did not affect degradation, are the PYD and/or NACHT domains responsible for this? This point can be discussed.
11. Page 9, line 29 - 32: The conclusion that NLRP10 is not the seed for ASC filaments is drawn from overexpression data (Supplementary Fig. S6B). Firstly, ASC specks still colocalize with NLRP10 oligomers upon activation. Secondly, prior studies (PMID: 36941399, 36941400) reported NLRP10-ASC interaction. These points warrant discussion.
12. Page 11, line 14. The citation appears to be incorrect.
13. Immunofluorescence is missing in Methods.
14. Page 15. Immunoblot Analysis. Antibody against NLRP10 is missing.
15. Page 18, Fig. 2F. Providing estimated molecular weights for each peak would be informative to determine the oligomeric state (i.e., the number of monomers per oligomer). SEC-MALS analysis can do this.
16. Page 18, Fig. 2G: The loss of ATPase activity in the NLRP10ΔHD2tail monomer requires explanation. Is the tail required for ATPase activity? Does Peak 2 represent an auto-inhibited conformation? If so, this suggests that the tail is not required for NLRP10 auto-inhibition. This should be clarified.
17. Page 19. Fig. 3A and 3E: NLRP10 and GAPDH should be labelled for the immunoblots.
18. Page 19. Fig. 3: hNLRP10Δtail and hNLRP10Δtail + mouse tail are expressed much higher than other constructs. Their slower turnover rate might be due to the saturation of ubiquitin-proteasome system. For a valid comparison, the authors can try transfecting different DNA amounts to get comparable expression levels at the 0 h time point.
19. Page 19. Fig. 3. Is the "all mutated" NLRP10 more stable? Testing this would assess whether the predicted K605 is the target for ubiquitination. Alternatively, proteasome inhibitors (e.g., MG132) can be used.
20. Supplementary Fig. S1C: Please specify which NLRP10 antibody was used for immunofluorescence. The authors referred to PMID: 22672233, but there are 3 of them in this paper: 4B4, 8H2, or 8B3?
21. Supplementary Fig. S1C: The NLRP10 signal in the nucleus is unexpected and questions the specificity of the antibody.
22. Supplementary Fig. S1C and S1D: The lack of IL-18 secretion could alternatively be explained by low endogenous NLRP10 expression in HaCaT cells. Similar results have been reported in other cell models (PMID: 36941400). The authors should provide immunoblots to confirm high level of endogenous NLRP10 levels in HaCaT and verify its knockout, as was done for HeLa in Supplementary Fig. S2D. This is critical for the claim that NLRP10 does not form classical inflammasomes.
In addition evidence needs to be provided that IL-18 is expressed in HaCat
23. Supplementary Fig. S1: Does poly(I:C) enhance NLRP10 expression in HaCaT cells as it does in HeLa? Can it increase IL-18 secretion combined with m-3m3FBS in HaCaT, just as reported in PMID: 36941399? These experiments would further clarify NLRP10's capacity to form functional inflammasomes.
24. Supplementary Fig. S5: These experiments require quantification to exclude that there is no co-localization
25. Supplementary Fig. S6C: Please specify which NLRP10 antibody was used. The nuclear signal for NLRP10 requires explanations. Given that HeLa lacks endogenous NLRP10 (page 5, line 20), the antibody's specificity in this assay should be verified.

Referee #3:

In their manuscript, the authors investigate the NLRP10 sensor protein that can form inflammasomes despite lacking an LRR region that is present in other inflammasome-associated NLRs. They show using cell biological and in vitro assays that NLRP10 forms oligomers and appears punctate in cells after treatment with m3M3FBS (which should be expanded in the abstract). Oligomerisation was independent of the residues required for ATPase activity and the N-terminal PYD, but required the C-terminal tail. This region contains several lysine residues, which the authors suggest makes it intrinsically disordered and enables it to be recruited to lipid droplets. They also perform experiments that suggest that the presence of the C-terminus decreases the stability of the NLRP10 protein.

The experiments are carefully performed and well-described, however, much of the cell biology is with overexpression systems in HEK and HeLa cells, which do not naturally express NLRP10 and do not support its full activation. There are also some discrepancies with previous work on NLRP10 activation in macrophages (such as which regions of NLRP10 are required for inflammasome formation), which need to be clarified and/or discussed. Overall, the authors could have investigated more endogenous settings by using their WT and NLRP10KO HaCaT cell system, which is relegated to supplementary figs. While the biochemical data are interesting, their relevance to NLRP10 signalling remains unclear.

Major points:
1. Much of the work is in HeLa or HEK cells, can the authors demonstrate that the C-terminal region, lysines and oligomerisation at lipid droplets is involved in NLRP10 activation in a physiologically relevant setting? For instance, it would be good to see whether the results hold in HaCaT NLRP10KO cells expressing the NLRP10 mutants the authors have made and correlating assembly with inflammasome activation. Is IL18 or IL-1 expressed in HaCaT cells? Is the lack of IL18 production due to the lack of expression?
2. m3M3FBS is a non-specific activator of mitochondrial damage and inflammasome activation. NLRP10 is proposed to be activated by thapsigargin as well, have the authors considered this activator to see how broadly applicable their findings are?
3. The expression assays with deletion of C-terminus need additional time points to comment on stability or half-life.
4. Their data suggest NLRP10 oligomerisation occurs at lipid droplets, but whether this is relevant to inflammasome assembly is

not clear.
5. What is the effect of pIC treatment, which in previous studies in the authors' own data in SFig 1 suggests is an inducer of NLRP10 expression, on behaviour of NLRP10 mutants?
Minor point:
1. pg5 line 17, the aa range listed for both mutants is the same, please correct this
2. pg5 line 24, remove 'only' after PYD
3. pg 6 line 13, change underwent to undergone
4. pg 6 line 19 - what do the authors mean by 'early diverged from this cluster'? More rigorous phylogenetic analysis is required to support such statements.

UNIVERSITÄT HOHENHEIM

Universität Hohenheim (180b) | 70599 Stuttgart

Faculty of Natural Sciences
Institute of Nutritional Medicine
Department of Immunology

Prof. Dr. rer.nat. Thomas A. Kufer
Chair of Immunology

T +49 711 459 24850
F +49 711 459 24855
E thomas.kufer@uni-hohenheim.de
https://immu.uni-hohenheim.de/en

EMBO Reports

Achim Breiling
Senior Editor

15.04.2026

Dear Dr. Breiling,

Please find enclosed the revised version of our manuscript "C-terminal lysine residues localise NLRP10 at lipid droplets and govern NLRP10 oligomer formation" that we would like to re-submit to *EMBO Reports*.

We thank you for handling of our manuscript and the reviewers for their constructive criticism and their insightful comments that helped us to further improve our work. We thoroughly addressed all points raised by the reviewers. A detailed point-by-point response to the reviewers' queries is provided below. We hope that the revised manuscripts meet the requirements for publication in EMBO Reports.

Following the revisers suggestions, we included in the revised version novel experiments with another NLRP10 activator. We also added novel data on differences between NLRP3 and NLRP10 activation and toned down our statements on this point. Concerning localisation of NLRP10 at lipid droplets we now also provide automated quantification of our image data, and we elaborated on the experiments on protein stability. These new data fully supported our initial observations and conclusions.

All authors agreed on submission of the manuscript and declare to have no conflict of interest. Thank you again for consideration of our manuscript.

Sincerely,

UNIVERSITÄT HOHENHEIM
Institut für Ernährungsmedizin
Fg. Immunologie
Fruwirthstr. 12
70599 Stuttgart
https://immu.uni-hohenheim.de/

BADEN-WÜRTTEMBERGISCHE BANK
IBAN DE20 6005 0101 0002 5601 08
BIC-Code SOLADEST600
UST-ID DE 147 794 207

ANFAHRT
Stadtbahn
U3, Plieningen (Universität Hohenheim)
Bus
65, 70, 73, 74, 75, 76, 79

Zertifikat seit 2004 audit familiengerechte hochschule

Point-by-point response

Referee #1:

General Comments:
• English language is generally good, with a few sentences that could use clarification. Some examples are highlighted in the specific comments.
• Line numbers in the merged version reset every page.
• Figure quality is generally good
• K179 is sometimes written as K197
• The word "fixated" is used instead of "fixed"
Response: We thank the reviewer for her/his time for critical reading our manuscript and for the general positive evaluation of our work. In the revised version of our manuscript we corrected the above-mentioned typing errors.

• The relationship between NLRP10 and ASC is unclear. Earlier in the paper (Line 22) it is suggested that "some NLRP10 aggregates recruited ASC" but later ASC recruitment is suggested to precede NLRP10 aggregation. The proposed connection between NLRP10 and ASC should be clarified.
Response: We are sorry for this inconsistency in our wording. We have rephrased the text in line 22 (now page 4 lines 24-26) accordingly to better describe that we actually observed NLRP10 and ASC co-localisation. Upon over-expression we observe recruitment of NLRP10 to ASC specks, which spontaneously form in HeLa cells. Both, full-length NLRP10 and NLRP10Δ584 are recruited by these ACS specks, but not NLRP10ΔPYD. This recruitment is independent of treatment of the cells and does not resemble ASC speck formation induced by NLRP3. For NLRP3, the observed speck quickly resulted in cell death, and nearly all cellular NLRP3 was recruited to ASC (Fig. EV6D). By contrast, for NLRP10 we always observed reminiscent cellular NLRP10 not co-localized to ASC. This is now visualized and quantitatively analysed in the new figure EV6 panels A and D.

• It is unclear throughout and in the title whether NLRP10 is proposed to be oligomerising and/or clustering. Both words are used but to me these present significantly different situations. The proposed model and order of events should be clarified.
Response: We thank the reviewer for pointing this out. We agree that our wording might not have been precise enough to adequately describe NLRP10 localisation. In the revised manuscript we now refer to the localisation consistently as oligomer formation, as we are confident that NLRP10 forms oligomers. We have changed our wording throughout the whole manuscript, e.g. on page 11 line 15 ("are the actual signal for NLRP10 oligomerisation.") and on page 12 line 13 ("that this domain facilitates NLRP10 oligomerisation at lipid rich compartments in the cells").

• The tail and lysine residues are described as "conserved" in places (Line 24: "Collectively, the highly conserved C-terminal tail of NLRP10 is necessary for protein...") but it is not always clear how relevant this conservation is. Conservation should be put in some kind of context - for example, showing that lysine residues are more highly conserved than other residues in the tail, or

perhaps showing that the tail is more conserved than PYD, which is suggested to be less important compared to other NLRs. IDRs are notoriously difficult to align and residues do not necessarily need to show aligned conservation to be functionally important.

Response: We thank the reviewer for his/her helpful comment. The conservation of basic residues plays an explicit role in the tail as they are responsible for the high pI, and lysine is the most abundant amino acid in the NRLP10 tail (please see source data of Fig 3H), which is not the case for other NLRPs. Indeed, the lysine residues are even more conserved compared to other amino acids in the tail (please see Source data Fig EV3), indicating that the high pI is a general feature of NLRP10. The remainder of the protein including the PYD also underwent relatively few evolutionary changes of the amino acids, leading e.g. to an identity of >95% in the Hominidae. Only the tail underwent such drastic changes (e.g., deletion of 2.7 kb in the genomic region coding for the tail). We have edited this paragraph in the manuscript to better point out that the lysine residues are the most frequent amino acids ("the amino acid with the highest abundance in the tail") and we also added the paragraph discussing the 2.7 kb deletion.

• The ubiquitination aspect is predicted but not followed up or discussed - this could be removed to shorten and focus the manuscript.

Response: We agree with the reviewer that we did not follow up on the ubiquitination in more detail. In the revised manuscript we include novel experimental data analysing stability of NLRP10 K0 (see also request by reviewer #3), and show that this protein is as stable as the Δ584 deletion mutant, highlighting that the lysine residues are important for the stability of NLRP10. We rephrased this paragraph to emphasise that ubiquitination is one possible mechanism for degradation based on bioinformatic prediction. See page 7 lines 2-5.

Specific Comments:

Abstract

• Line 9, spelling?: "Convers"

Introduction

• Line 4, additional "a": "(nucleotide-binding..."

Response: These mistakes were corrected in the revised manuscript.

• Line 27: "The structures of the..." The argument at the end of this paragraph is slightly unclear to me. Is it that orthologous proteins (including hs/mmNLRP10) can have different functions? There are different copy numbers of NLRP1, is the situation comparable to NLRP10?

Response: NLRP10 does not have different transcription variants or splice variants in mice or men, at least to our knowledge. We highlight that many protein orthologs are actually functionally divergent, and this might also be the case for NLRP10. We edited the sentence for clarity (page 3 line 30).

• Line 36, missing word: "Further understanding of the role..."

Response: We corrected the grammar.

Results

Oligomerisation of NLRP10 upon m-3M3FBS treatment is ASC- and ATP hydrolysis-2 independent

• Line 12/Figure 1: "We observed..."

o Could the authors supplement an image series that was not treated with m-3M3FBS to demonstrate that the loss of mCherry signal is not due to photobleaching?
o Alternatively, could the authors indicate in methods that the signal display is normalised to the first frame?
• Line 15/Figure 1: "Thereby..."
o Are these structures circular or more generally aggregated? Can roundness be quantified?
Response: We appreciate the comments which helped us to improve this part of the study. We now added an additional panel including insets of *m*-3M3FBS and vehicle (DMSO) treated cells in the figure EV1 (former Supple Fig 1), showing the circular structures and that the loss of signal is not due to photo-bleaching. Additionally, in the expanded and revised Figure 4, panels D-E, we now included automated machine-learned quantification of sphericity (a measure of how closely an object is to a perfect sphere or circle) for NLRP10 oligomers (green).

• Line 21/Figure 1B: "Upon m-3M3FBS treatment..."
o Is it possible to quantify the ASC specks in the same way as the NLRP10 aggregates in 1C?
o Alternatively, could the authors note that these FOVs are representative of the general cell population?
Response: In the live-cell experiments we have not quantified ASC specks, as they were not dependent on expression of NLRP10 (also eGFP cells show ASC specks). The represented images are representative for multiple cells and for several experiments. We added to the figure legends that this represents the general cell population.

o Do the authors have an example of an NLRP10 aggregate forming followed by an ASC speck forming on top? This would be a more convincing demonstration of recruitment (Following the description of order of events in Discussion section/S6, I recommend removing or clarifying references to ASC recruitment)
Response: We did not observe NLRP10 oligomers that recruited ASC. We are sorry for this confusion due to our apparently unclear descriptions. On the contrary, we observed ASC specks that recruited NLRP10 and NLRP10Δ584 without treatment, but not NLRP10ΔPYD. When cells were treated with *m*-3M3FBS, NLRP10 and NLRP10ΔPYD but not NLRP10Δ584 oligomerises, highlighting that oligomerisation and ASC binding are independent. To clarify this, we have expanded and revised figure EV6 to show that ASC specks precede NLRP10 oligomer formation while residual NLRP10 oligomers were dispersed throughout the whole cell and did not recruit ASC.

• Line 25/Figure S1C/D: "As for..."
o Was S1C performed in the KO cells also?
Response: We have not used knockout cells in this experimental setting. Previously we have shown specificity of our antibody (Lautz et al., 2012. *Cellular microbiology)*.

o The relevance of the positive control in S1D is unclear to me
♣ The possibility that NLRP10 releases IL-18 in a canonical manner is being tested: NLRP10 ◊ ASC ◊ Caspase-1 ◊ IL-18 processing
♣ Poly(I:C) IL-18 release would not involve an inflammasome, it is unclear that these cells can

undergo pyroptosis under these conditions

♣ Cell type and time point for positive control are not listed

Response: We thank the reviewer for these comments and we are sorry for the unclear description of the figure. In the revised figure (now figure EV1) we included measurement of IL-18 release in wt and NLRP10 knockout HaCaT cells. We used poly(I:C) to induce the expression of NLRP10 protein. Immunoblot of NLRP10 expression is now included in the same figure (EV1 D). Upon *m*-3M3FBS and poly(I:C) treatment we can measure IL-18 release into the supernatant, so the cells are able to process IL-18, one hallmark of an inflammasome. We did not observe a difference between HaCaT and NLRP10-deficient HaCaT cells, strongly suggesting that this is not NLRP10-dependent as suggested by the reviewer. Concerning an experiment using over-expression of caspase-1 and ASC, we want to point out that HaCaT cells express caspase-1, ASC, GSDMD and IL-1β and can undergo pyroptosis (He et al., 2015. *Cell research;* Zhang et al., 2025. *International journal of radiation biology)*, additionally we show that our cells do secrete IL-18. See also comment to reviewers #2 and #3.

NLRP10 aggregation depends on its C-terminal tail region

• Line 36/Figure S2A: "Super-positioning..."

o Does this contribute to the discussion of the C-terminal region? Only the NACHT is shown to overlay here.

Response: The reviewer is right that the tails do not overlay in our model. This is likely due to the inability of the AlphaFold model to adequately predict the tail conformation and the facts that this is an intrinsically disordered region (IDR). We do not expect, for neither the mouse nor the human NLRP10, that the tail looks like the represented tail. Still we think that the figure is useful for the reader to visualize the IDR. We clarified this in the text (page 5 lines 12-14).

• Line 9, spelling: "obtained for either the human or the..."

Response: We corrected the double-negation error.

• Line 29: "Proteins were separated..."

o Could the authors supplement the SDS PAGE corresponding to the peaks? A pure sample should be used to assess ATPase activity but contaminants often copurify in the void.

Response: We thank the reviewer for pointing out that the SDS PAGE was missing in the original manuscript submission and added this in figure EV2 panel G of this revision. As can be seen in all three protein purifications of wild type NLRP10, the K179A mutant, and the ΔHD2–tail truncation, both peaks 1 and 2 were always purified to homogeneity, indicating no signs of copurified contaminants or protein degradations. We added this in the manuscript (page 5 lines 36-37 and page 6 lines 9-10)

The reduced ATP turnover rate of the Walker A NLRP10 K179A mutant results from the lower affinity of the ATP substrate for this protein variant. A quantification of the slop of this measurement indicates an approximately three-fold reduced ATP hydrolysis activity for the K179A protein compared to wild type. Of note, this fold in reduction perfectly matches with the previous description of the Walker A residue mutation K232A in NLRP3, which has been determined to 0.34 compared to wild type (Brinkschulte et al., 2022. *Communications biology)*.

• Line 35: "As expected..."

o Can this significance be tested and quantified?

Response: We appreciate the reviewer's comment, for us the graphs were obvious, so we did not consider adding the statistics into the text. We definitely agree that this strengthens the data and added statistical significance levels into the text (page 7). However, we did not add statistics into the figure as this would reduce the visibility of the curves to our view.

• I am not clear on the argument behind the recombinant protein work. Either main text or supplementary commentary might help:

o "if ATP binding, which is required for an active protein conformation..." Which peak (if any) correspond to the active conformation in this assay? WT and WA mutant have a similar SE profile and all three have the void peak.

Response: It is right that all analysed recombinant proteins exhibit high molecular weight aggregates that elute early in size exclusion chromatography, giving rise to peak 1. It is also true that the formation of peak 1 is not induced simply by the addition of ATP, nor that these peak fractions contain an enrichment of protein bound ATP. We rather consider the NLRP10 protein in peak 1 as a conformation that is not any more autoinhibited. Similar to NLRP3 and as the AlphaFold3 structure predicts, we assume that the N-terminal FISNA subdomain within in NACHT is covering the nucleotide-binding site in NLRP10, rendering the nucleotide exchange from ADP to ATP impossible. This coverage of the nucleotide-binding site is lost in the protein that assembles into peak 1. In that sense, peak 1 resembles a protein conformation that lost autoinhibition, readily accessible for the ATP nucleotide.

o Void peaks - Are these PYD-driven filaments?

Response: This is an interesting question and we are happy to provide more information on the integrity of the protein in the void peaks. When analysing peak 1 in negative stain electron microscopy images, no indications for filament formation were observed. The homomeric PYD–PYD interaction is thus not the driving force for the protein assembly in peak 1. In addition, we like to note that the structure of the NLRP10 PYD has been determined by NMR spectroscopy (Su et al., 2013. *PloS one)*, indicating a monomeric state even at high concentrations. Furthermore, we could not observe any signs of filamentous assemblies when purifying the PYD alone.

o Oligomers - NACHT-driven disks? Tail-driven oligomers?

Response: We find that the oligomerization observed for WT and K179A NLRP10 is driven by the NACHT domain; however, we could not identify any disk formation, e.g. similar to NLRP3–NEK7 or NAIP/NLRC4, in negative stain electron microscopy for peak 2 (nor peak 1). The oligomers require an intact HD2 and the IDR of the tail sequence, but the homomeric assembly is not clear on a structural level.

o Monomerisation of truncated protein - Can the effects of removing HD2 vs removing tail be distinguished? Was a Δtail construct purified, alternatively a literature reference indicating that removal of HD2 does not affect oligomerisation of NLRs?

Response: This is an interesting question regarding comparative insights into the role of HD2 and the IDR in protein oligomerization. Together with the linker region between the PYD and NACHT

domain, the HD2 and transition LRR (trLRR) region are the most variable region in the 22 human NLRs (Hochheiser und Geyer, 2023;). In fact, the IDR in NLRP10 can be considered a variation of the trLRR, which harbours for example an acidic loop in NLRP3 or a basic loop in NLRP6. While the acidic loop is instrumental in the face-to-face interaction of NLRP3 in the ADP-bound state and thus in oligomerization to the cage structure, we do not have any more structural information about the function of this section in other NLRs. To our knowledge, no literature of the deletion of HD2 in any other NLR exists, that would give insights into the role in complex formation.
Crystal structure of NLRP3 (131-679) leads to monomer formation (Dekker et al., 2021. *Journal of molecular biology)*.

Evolutionarily conserved basic residues in the C-terminus of NLRP10 mediate oligomerisation
• Line 13: "NLRP10 seems to have underwent complex..."
o Could the authors clarify this sentence?
Response: Following the reviewer's suggestion, we have rephrased the whole paragraph and included additional information to explain the evolution event (page 6 line 16 – page 7 line 1).

• Line 37: "Deletion of the human..."
o Could authors be more consistent in descriptive language here? The non-significant increase in Nlrp10 from Figure 3B is described as "an increase" while the non-significant decrease in Nlrp10 from Figure 3C is described as having no effect - I don't think this influences author conclusions but would improve clarity
Response: We apologise that this was not well described. We revised this part and included the values and p values into the main text to allow the reader to independently interpret the data (see page 7).

• Line 34: "Mitigates" - Is this the correct word?
Response: This was a mistake and should read "facilitate". We corrected this accordingly.

• Figure 3J:
o Was an equivalent of 3J done with Nlrp10?
o Did mutating non-basic residues have a similar effect?
Response: We thank the reviewer for raising these points. We have not generated stable cell lines expressing mouse Nlrp10 K0, as we do not think that this will add novel information over the provided data.
We agree that it would be informative to mutate other residues to understand if structural aspects or simply the pI influences the function of the tail. However, we hope the reviewer can understand that such elaborate additional analyses are beyond the scope of this first report on the function of the C-terminal domain of NLRP10.

The NLRP10 oligomers are located at lipid compartments
• Line 6: "We found that..."
o These have not been described as or shown to be ring-like up until this point. Is a supplementary figure missing?

Response: We thank the reviewer for spotting this inconstancy. Fig 1B shows that these are circular structures, but we did not make the link in the initial version of the manuscript. In the revised version, we included a new supplementary figure (EV1 B), in which these circles are clearly visible and in the text we connect the sentence to mention Fig. EV1 B. [“Thereby NLRP10 formed cytosolically dispersed circular structures (Suppl. Fig. S1B) rather than single specks”]. Later in the manuscript we follow up on this point and show that NLRP10 oligomers are as spherical as lipid droplets and provide measurement of sphericity (see response above).

Discussion

• Line 10: "This segment..."

o Could the authors clarify the connection between the NLRP10 tail and NLRP3 loop?

Response: We thank the reviewer for this suggestion, as it covers an interesting aspect of the domain architecture of NLRs. The approximate ~250 aa sequence between the winged-helix domain (WHD) and the canonical leucine-rich repeat domain (cnLRR) is the least conserved region within NLRs. The presence of the helical domain 2 (HD2) is a defining feature of STAND ATPases, classifying them as subfamily of AAA+ ATPases. The accompanying transition LRR region (trLRR) is typically not well predicted by sequence homology, but becomes apparent in AI-supported structure predictions such as AlphaFold2. Interestingly, almost all NLRs contain a loop section of variable length and amino acid characteristics within the trLRR before being discharged into the canonical LRR. This has been first resolved for NLRP3 with the 42-residue long acidic loop (683-724, pI 5.3), and is predicted as 38-aa long basic loop in NLRP6 (678-715, pI 11.3) or 21-aa long basic loop in NLRP1 (771-791, pI 11.7). Of note, the corresponding region of this loop section has been deleted in the crystal structure of NLRC4, giving rise to the better crystallization properties of such protein construct. In this sense, the 72-aa long intrinsically disordered region (IDR) of NLRP10 (584-655, pI 9.5) can be regarded as a “variation of a theme” in the domain architecture of NLRs, where the loop section is extended to start right from the end of HD2. A short description of this region, which was initially also called “NACHT-associated domain” or NAD is given in: Hochheiser, I.V. and Geyer, M. (2023). Structural aspects of inflammasome-forming NOD-like receptors. In: Fundamentals of Inflammasome Biology (Editor: Pablo Pelegrín), Chapter 14, pg. 215–233 (Hochheiser und Geyer, 2023;).

• Line 13, spelling : "Is not dependent..."

Methods

• Can the authors clarify the following lines:

o "9.3 g 0.5 M EDTA"

o "wt 4-655 and mutant"

Response: We have corrected these errors appropriately.

Referee #2:

Major points:

1. Figure 3 currently combines data on NLRP10 protein stability and oligomerization. It would be

easier to interpret if Figure 3 focuses solely on NLRP10 stability, with the oligomerization data moved to other figures.
Response: Following the suggestions form the reviewers, we have included new data on the stability of NLRP10 K0 and rearranged and revised figure 3 accordingly. We hope that this now provides a cleared outline and a better separation of stability and oligomerisation data.

2. The use of only one activator (m-3M3FBS) limits the generalizability of the conclusions. The study would be strengthened by including one or more published NLRP10 activators (e.g., thapsigargin, SMBA1; PMIDs: 36941399, 36941400). Furthermore, experiments using known inhibitors such as cyclosporin A would be valuable to confirm the specificity of the activation pathway.
Response: We thank the reviewer for this important input. We absolutely agree that testing other substances that can induce NLRP10 oligomerisation would increase the impact of the study. The list of described NLRP10 activators is currently short, so we performed novel experiments with one additional activator and added these data in the revised version of the manuscript (Fig. 1B). We used SC-10, a compound that was described by Prochnicki in his doctoral thesis (Próchnicki, 2021. https://bonndoc.ulb.uni-bonn.de/xmlui/handle/20.500.11811/9261). SC-10 induced NLRP10 oligomerisation at lipid droplets. Deletion of either the tail (Δ584) or the lysine residues in the IDR (K0) did block the SC-10-dependent oligomerisation. This new data fully supports our conclusions and is shown in the revised and expanded figure 1 panel B and figure EV5 panel C. However, we respectfully disagree with the reviewer that the use of cyclosporin A would be helpful. We do not see how the mPT inhibitor cyclosporin A would confirm the specificity of induction of NLRP10 oligomerisation. Moreover, Prochnicki et al. showed that cyclosporin A did not block *m*-3M3FBS induced mitochondrial rupture and subsequent NLRP10 activation. In his doctoral thesis Prochnicki shows that cyclosporin A also did not inhibit inflammasome activation upon SC-10 (Próchnicki, 2021. https://bonndoc.ulb.uni-bonn.de/xmlui/handle/20.500.11811/9261).

3. The study includes a comparison of a number of different NLRP10 constructs (deletions, mutations) as well as human and mouse protein. Given that expression levels could affect oligomerization of the protein, the authors need to include immunoblots or FACS analysis confirming equal expression of all proteins analyzed.
Response: The reviewer very well observed that the amount of detectable protein in the different cell lines varied. We want to clarify that we used the same maternal cell line for the generation of all of our stable cells and the DNA encoding the proteins of interest is integrated at the same genetic location. The system is very robust and thus we expected equal expression. However, the Δ584 and K0 variants are both more stable (see Fig. 4) and this might lead to the different protein levels. Obviously, this could be a confounder of oligomerisation. However, we would like to argue that more protein would rather enhance aggregation of the protein, which is opposite to our findings.

Minor points (all page and line numbers are for the merged PDF):
1. In general, typo errors are found throughout the manuscript. A thorough proofreading is recommended. For example, Page 18, Fig. 2E: NLRP10 should be italicized. Page 11, line 1: The first "m" should be consistently italicized for m-3m3FBS.
Response: We carefully proof-read the manuscript and corrected corresponding errors accordingly.

2. Page 2, line 5: The claim that "ASC nucleation and recruitment were different to other NLRPs" is not supported by the authors' data, as they did not directly examine other NLRPs in their models.

Response: We thank the reviewer for pointing this out. To support our data, we now also used NLRP3 over-expression in the stable ASC-RFP (CMVd1 promoter). Upon Nigericin treatment, nucleation was observed and only one single speck was formed per cell (Fig. EV6D). Together with the different localisation of NLRP10, these observations suggest differences between NLRP10 and NLRP3. The reviewer is right in that we did not test other NLRPs. Accordingly, we changed these statements (Abstract and page 10), the respective data is now included in the manuscript.

3. Fig A: Nlrp10 aggregation is not that well visible, please include a quantification of aggregation. Include also a well-established marker for mitochondrial damage.

Response: We thank the reviewer for his/her point. We changed the contrast in the figure to better visualise the structures. For LCI experiments we have used the mitochondrial localised mCherry construct (Fig. 1A) to visualise the mitochondrial disruption as previously described (Próchnicki et al., 2023. *Nature immunology)*. Quantification of the aggregation of mouse Nlrp10 is provided in Figure 2, panel E, which was included in the initial submission.

4. Page 2, lines 15-16 (Fig. 1A): the claim that circular structures are visible is not well supported. Please include insets showing this.

Response: Following the reviewer's suggestion, we included additional insets found in the revised figure EV1, panel B. Moreover, we calculated the roundness of these structures by automatic image analysis (see Fig. 4D). See also comment to reviewer #1.

5. Page 4, line 32 and 33. The statement that "NLRP10 does not form classical inflammasomes" appears to contradict the data in Fig. 1B, which shows m-3M3FBS-induced ASC speck formation. This statement should be clarified. In addition, the authors should define if each ASC speck co-localizes with NLRP10, and if NLRP10 activation is the cause for ASC formation.

Response: We thank the reviewer for this suggestion and agree that the statement might have been misleading. In our stable HeLa cells expressing ASC-RFP at lower levels (CMVd1 promoter), we observe ASC specks recruiting NLRP10 and NLRP10Δ584, but not NLRP10ΔPYD (see also the response to reviewer #1). We did not observe NLRP10 oligomers that recruited ASC but rather the opposite. This is shown in figure EV6 panel B. The recruitment of NLRP10 to ASC specks in our hands and in this system appears to be different compared to NLRP3, where virtually all NLRP3 is co-localised with the ASC speck, for NLRP10 most signal remains cytosolic. We added novel data on this and provide also a quantification of the NLRP10 signal, shown in the revised figure EV6 panel D. Upon treatment with *m*-3M3FBS, we did not observe further ASC specks. We also did observe ASC specks that did not co-localize with NLRP10 (see figure EV6 A). While our data show that NLRP10 and NLRP3 recruit ASC differently, our data does not exclude formation of an NLRP10 inflammasome. To address the reviewers concern, we carefully edited the respective text sections and only refer to NLRP3 as control.

6. Figure 1C: Does K179A induce ASC speck formation, and how does this compare to WT

Response: As discussed above, in our system we do not see clear ASC speck induction by NLRP10. To address the question of the reviewer, we expressed NLRP10 K179A in our stable ASC cell line. We observed some ASC specks that recruited K179A, but most ASC specks did not recruit K179A. Due to space constraints we unfortunately cannot show this as additional data, which is provided here for the reviewer:

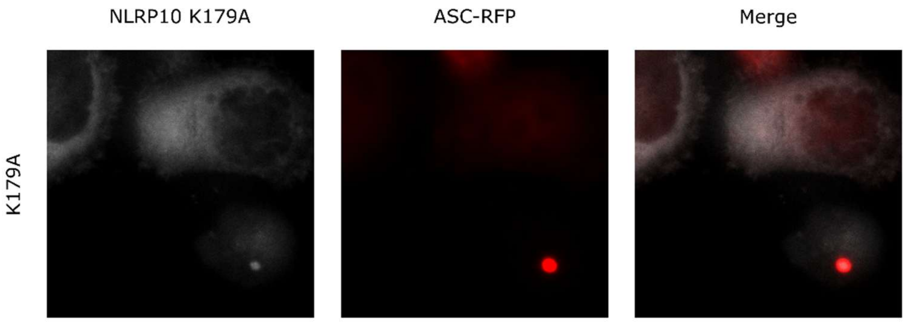

7. Fig. 2F-G: How does the deletion of HD2 in other NLRs affect ATP hydrolysis activity? Is it similarly lost as for Nlrp10?
Response: This is an interesting question. As mentioned in the comment to reviewer #1, not much information is available on the role of HD2 in other NLRs that could be compared to NLRP10. However, a crystal structure has been determined for NLRP3 (131-679), which showed to be monomeric as recombinant protein in SEC as well as in the crystallographic lattice. The construct was also characterised for its ATP hydrolysis activity and showed significantly reduced ATP turnover rates compared to full length NLRP3 (unpublished results, M.G.). Besides that, we are not aware of any other characterisation of the role of HD2 in NLR protein assembly and hydrolysis activity. We hope that the reviewer can understand that experimentally testing this for several other NLRs would be beyond the scope of this report.

8. Fig. 4D: does 3M3-FBS induce droplet formation? and what percentage of NLRP10 speckles co-localize with lipid droplets?
Response: We thank the reviewer for bringing up this important point. In the revised version of the manuscript, we conducted novel experiments and automatic recognition and quantification analyses (Arivis pro) of NLRP10 and LDs in cells to address this point. We did not observe a statistical difference of the lipid droplet count per cell upon *m*-3M3FBS in HeLa cells, which is shown in the revised Figure 4, panels E-F. Indeed, already at 0 min, lipid droplets were well visible. We further quantified the NLRP10 oligomers and their co-localisation with lipid droplets. This revealed that ~2/3 of all NLRP10 oligomers co-localised with lipid droplets. This new data is shown in the revised and expanded Figure 4, panel F.

9. Page 6, line 2 - 3: The claim that Peak 2 contains monomers needs to be supported. The explanation for why the monomers lose ATPase activity is missing and needs to be elaborated.
Response: We thank the reviewer for pointing this out. We could nicely determine the molecular weights for the concentration dependent monomer-to-dimer transition of MBP-NLRP10ΔHD2–tail,

which is now shown in supplementary figure EV2, panel H, of the revised manuscript. A common feature for AAA+ ATPases is that monomers do not possess an ATPase activity; we added a sentence in the respective section and the respective citation (Wendler et al., 2012. *Biochimica et biophysica acta)*.

10. Page 7, line 4 and 5: Given that tail deletion in mouse Nlrp10 did not affect degradation, are the PYD and/or NACHT domains responsible for this? This point can be discussed.
Response: We agree with the reviewer that we did not test the effect of PYD and NACHT deletion on the stability of mouse Nlrp10. This would surely be interesting for further research. Given the differences in the PYD between mouse and human NLRP10 (Su et al., 2013. *PloS one)*, one could speculate these might be one driver for the differences in the protein stability. A consideration of this aspect was added to the revised Discussion section (page 11 lines 34-37).

11. Page 9, line 29 - 32: The conclusion that NLRP10 is not the seed for ASC filaments is drawn from overexpression data (Supplementary Fig. S6B). Firstly, ASC specks still colocalize with NLRP10 oligomers upon activation. Secondly, prior studies (PMID: 36941399, 36941400) reported NLRP10-ASC interaction. These points warrant discussion.
Response: Concerning ASC speck induction, we would like to refer the reviewer to our answers provide above. In our cellular system, we did not clearly observe NLRP10 oligomers that recruit ASC. We want to point out that this does not contradict the studies by Próchnicki et al., 2023. *Nature immunology;* and Zheng et al., 2023. *Nature immunology* as they have used other cells with different models of over-expression. Comparing Figure 4d from (Próchnicki et al., 2023. *Nature immunology)*, shows that they also observe multiple NLRP10 “specks” and multiple ASC specks per cell, which is indeed very similar to our findings. We think that both features of NLRP10 might depend on the cell model. As mentioned above we have edited the text to make this clear.

12. Page 11, line 14. The citation appears to be incorrect.
Response: We thank the reviewer for the very careful check of our manuscript. Indeed, one citation was missing here. We corrected this in the revised version.

13. Immunofluorescence is missing in Methods.
Response: We added this in the revised version.

14. Page 15. Immunoblot Analysis. Antibody against NLRP10 is missing.
Response: We apologise for these mistakes and thank the reviewer for noticing. In the revised version each information was added to the methods part (page 15 and 17 sections immunofluorescence and immunoblot).

15. Page 18, Fig. 2F. Providing estimated molecular weights for each peak would be informative to determine the oligomeric state (i.e., the number of monomers per oligomer). SEC-MALS analysis can do this.
Response: We thank the reviewer for this suggestion and performed SEC MALS measurements on the various elution profiles. We could nicely determine the molecular weights for the concentration dependent monomer-to-dimer transition of MBP-NLRP10 (4-482), which is now shown in

supplementary figure EV2, panel H, of the revised manuscript. Unfortunately, for full length NLRP10 WT and K179A protein the elution profiles on a Superose 6 column were not distinct enough in order to determine the molecular masses with reasonable precision. That said, we would estimate that peak 2 of WT and K179A correspond to a hexamer and possibly dodecamer assembly of NLRP10.

16. Page 18, Fig. 2G: The loss of ATPase activity in the NLRP10ΔHD2tail monomer requires explanation. Is the tail required for ATPase activity? Does Peak 2 represent an auto-inhibited conformation? If so, this suggests that the tail is not required for NLRP10 auto-inhibition. This should be clarified.
Response: We thank the reviewer for this insightful question. As pointed out above, we belief that peak 2 represents an autoinhibited conformation, resulting in a reduced ATP hydrolysis activity. The monomeric fraction of the NLRP10 ΔHD2tail mutant (peak 2) exhibits indeed the lowest hydrolysis activity. This could indicate that the transition to an active conformation is more easily achieved from the oligomeric peak 2 fractions in the WT and K179A mutant proteins, compared to the monomeric NLRP3 ΔHD2tail fraction. In other words, the autoinhibition is more tightly preserved in the monomeric conformation compared to the oligomeric state. Similarly, as pointed out above, this observation is reminiscent to the peak 1 and peak 2 fractions in NLRP3, compared to the monomeric NACHT domain that was shown to have the least hydrolysis activity.

17. Page 19. Fig. 3A and 3E: NLRP10 and GAPDH should be labelled for the immunoblots.
Response: We include these labels in the revised figure 3 panel A.

18. Page 19. Fig. 3: hNLRP10Δtail and hNLRP10Δtail + mouse tail are expressed much higher than other constructs. Their slower turnover rate might be due to the saturation of ubiquitin-proteasome system. For a valid comparison, the authors can try transfecting different DNA amounts to get comparable expression levels at the 0 h time point.
Response: We agree with the reviewer that the different expression level in principle could be a concern. Using lower DNA amount in our hands, however, resulted in less cells being eGFP positive and not reduced expression of NLRP10 in transfected cells. We conducted novel experiments and generated a stable cell line expressing the NLRP10 K0 variant. With these cells, we used different length of induction by Dox to analyse if this impacts the decay and observed consistent decay with the same amplitude. This corroborates our initial data and our conclusion. The new data is presented in Figure 3, panels I-K, using 20 h Dox induction for both cell lines.

19. Page 19. Fig. 3. Is the "all mutated" NLRP10 more stable? Testing this would assess whether the predicted K605 is the target for ubiquitination. Alternatively, proteasome inhibitors (e.g., MG132) can be used.
Response: We thank the reviewer for this interesting question. To this end, we established stable HeLa cells expressing NLRP10 K0. This novel cell line allowed us to follow the decay of the protein more precisely. As anticipated, we observed a faster decay for NLRP10 wt compared to NLRP10 K0. This new data is now included in the revised Figure 3, panels I-K. Together, this suggest that NLRP10 K0 indeed is more stable and supports that K605 might be a target for ubiquitylation. This information can be found in the revised manuscript (page 7 line 34 – page 8 line 7).

20. Supplementary Fig. S1C: Please specify which NLRP10 antibody was used for immunofluorescence. The authors referred to PMID: 22672233, but there are 3 of them in this paper: 4B4, 8H2, or 8B3?

Response: We are sorry for this confusion, we used clone 8H2 and now added this information in the revised method section and in figure legend.

21. Supplementary Fig. S1C: The NLRP10 signal in the nucleus is unexpected and questions the specificity of the antibody.

Response: The signal that is observed in the nucleus represents background of the immunofluorescence staining, oligomers of NLRP10 are only found in the cytosol of the cells. For additional test of specificity of our antibody, which was also used in other recent NLRP10 publications, we would like to refer the reviewer to our initial characterisation of this tool in (Lautz et al., 2012. *Cellular microbiology)*.

22. Supplementary Fig. S1C and S1D: The lack of IL-18 secretion could alternatively be explained by low endogenous NLRP10 expression in HaCaT cells. Similar results have been reported in other cell models (PMID: 36941400). The authors should provide immunoblots to confirm high level of endogenous NLRP10 levels in HaCaT and verify its knockout, as was done for HeLa in Supplementary Fig. S2D. This is critical for the claim that NLRP10 does not form classical inflammasomes. In addition, evidence needs to be provided that IL-18 is expressed in HaCat

Response: We thank the reviewer for this suggestion and now included an immunoblot from the HaCaT cells showing a) basal expression of NLRP10, b) no expression of NLRP10 in the HaCaT knockout cells, and c) induction of NLRP10 protein levels by poly(I:C) treatment. This new data is shown in the revised figure EV1 panel D.

Poly(I:C) induced the release of IL-18 from our HaCaT cells into the supernatant, clearly showing that these cells produce IL-18. This control is now better described and shown in the revised figure EV1 panel E. See also our comment to reviewer #1.

23. Supplementary Fig. S1: Does poly(I:C) enhance NLRP10 expression in HaCaT cells as it does in HeLa? Can it increase IL-18 secretion combined with m-3m3FBS in HaCaT, just as reported in PMID: 36941399? These experiments would further clarify NLRP10's capacity to form functional inflammasomes.

Response: As discussed above, we now included immunoblots for NLRP10 expression in the revised figure EV1, panel D, showing that poly(I:C) induces NLRP10 expression. Upon poly(I:C) treatment we observed IL-18 secretion from the HaCaT cells, which was independent of NLRP10. This new data is shown in the revised figure EV1, panel E. Of note, *m*-3M3FBS alone, did not lead to IL-18 release in HaCaT cells, only poly(I:C) + *m*-3M3FBS, but independently of NLRP10. This can be seen in Fig. EV2E.

24. Supplementary Fig. S5: These experiments require quantification to exclude that there is no co-localization.

Response: Following the reviewer's suggestion, we have quantified these images. In accordance with our interpretation in the initial version, we only found partially co-localisation, with exception of the ER where no co-localisation with NLRP10 was observed. In the revised main text, we added that co-localisation was below 50 %. The quantitation data is shown here for the reviewer.

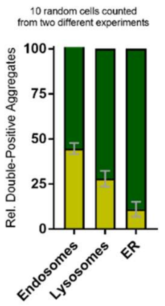

25. Supplementary Fig. S6C: Please specify which NLRP10 antibody was used. The nuclear signal for NLRP10 requires explanations. Given that HeLa lacks endogenous NLRP10 (page 5, line 20), the antibody's specificity in this assay should be verified.
Response: We thank the reviewer for spotting this. We now specified the antibody we used in the revised version of the figure legend (8H2). For the second part, we are sorry that the sentence might have been misleading. HeLa cells do express NLRP10 albeit at low levels (see our initial paper on NLRP10 (Lautz et al., 2012. *Cellular microbiology)*). The reviewer refers to a sentence where actually HeLa NLRP10 ko cells are meant. We agree that this sentence was misleading and changed to "HeLa cells in which we knocked-out endogenous NLRP10". The nuclear signal represents background staining.

Referee #3:

Major points:
1. Much of the work is in HeLa or HEK cells, can the authors demonstrate that the C-terminal region, lysines and oligomerisation at lipid droplets is involved in NLRP10 activation in a physiologically relevant setting? For instance, it would be good to see whether the results hold in HaCaT NLRP10KO cells expressing the NLRP10 mutants the authors have made and correlating assembly with inflammasome activation.
Response: We agree with the reviewer that most of the experiments have been performed in HeLa cells and that this is a limitation of our study. However, no HEK cells have been used in the study. In previous works we also others showed that HeLa cells are a good model to study inflammasomes (Chen und Chen, 2018. *Nature)*. Following the reviewer's suggestion, we performed novel experiments in NLRP10-knockout HaCaT cells expressing different NLRP10 variants. We observed oligomerisation of eGFP-NLRP10 and the eGFP-HD2-tail at lipid droplets in response to *m*-3M3FBS but not for the eGFP-NLRP10Δ584 or eGFP-HD2tailK0. This new data is shown in the revised figure EV5 in panel B.

Is IL18 or IL-1β expressed in HaCaT cells?
Response: We have not tested IL-1 expression; however, we show that treatment of HaCaT cells with poly(I:C) induces secretion of IL-18. This is shown in the revised figure EV1 in panel E. See also comments to reviewers #1 and #2.

2. m3M3FBS is a non-specific activator of mitochondrial damage and inflammasome activation. NLRP10 is proposed to be activated by thapsigargin as well, have the authors considered this activator to see how broadly applicable their findings are?

Response: We thank the reviewer for this important input. We now added experiments with another NLRP10 activator that corroborate our initial findings in the revised manuscript. We would like to refer to our response to reviewer #2, query 2:

"We absolutely agree that testing other substances that can induce NLRP10 oligomerization would increase the impact of the study. The list of described NLR10 activators is currently short so we performed novel experiments with one additional activator and added these data in the revised version of the manuscript (Fig. 1B). We used SC-10, a compound that was described by Prochnicki in his doctoral thesis (Próchnicki, 2021. https://bonndoc.ulb.uni-bonn.de/xmlui/handle/20.500.11811/9261). SC-10 induced NLRP10 oligomerisation at lipid droplets. Deletion of either the tail (Δ584) or the lysine residues (K0) did block the SC-10-dependent oligomerisation. This new data is shown in the revised and expanded Fig. 1B and EV5 C."

In addition, we see a similar effect with a novel NLRP10 activator that we obtained from a collaborator (as this compound and its activity has not been published we unfortunately cannot share this data)."

We hope that this convinces the reviewer that our finding is broadly applicable.

3. The expression assays with deletion of C-terminus need additional time points to comment on stability or half-life.

Response: We agree with the reviewer that comments on half-life of the protein in principle would benefit using more time-points. We performed assays with expanded time-line exemplified by the experiment to compare the NLRP10 and NLRP10 K0, of which the minimised figure is now included in the revised Figure 3, panel I-K. As the 30 h time point did not show differences to the 24h time point that we show in the figures, we do not think that this would increase the impact of the study significantly. At later time-points, (e.g. 50 h) the viability of the cells is drastically reduced, and we cannot exclude an artefact at such long incubation with CHX.

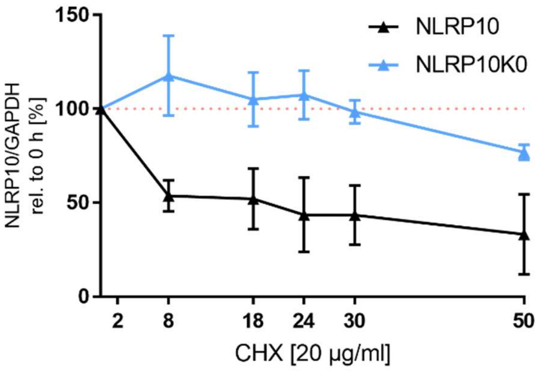

4. Their data suggest NLRP10 oligomerisation occurs at lipid droplets, but whether this is relevant to inflammasome assembly is not clear.

Response: We agree with the reviewer that our data does not allow to answer this question. Neither with *m*-3M3FBS nor with SC-10 we did observe a classical inflammasome, thus we do not know whether NLRP10 oligomers represent pre-forms of an inflammasome. Together with our findings that ASC recruited NLRP10 when over-expressed (and not vice versa), we think that these might represent two independent mechanisms, still NLRP10 oligomerisation might be a prerequisite for inflammasome formation.

5. What is the effect of pIC treatment, which in previous studies in the authors' own data in SFig 1 suggests is an inducer of NLRP10 expression, on behaviour of NLRP10 mutants?

Response: This is an interesting question. We see an increase of NLRP10 protein in HeLa and HaCaT cells upon poly(I:C) treatment. This is shown in the revised figure EV1 in panel D. We have extensively studied, whether poly(I:C) could also be an activator of the NLRP10 inflammasome; however, in our hands we have not seen oligomerisation of NLRP10 upon poly(I:C) exposure.

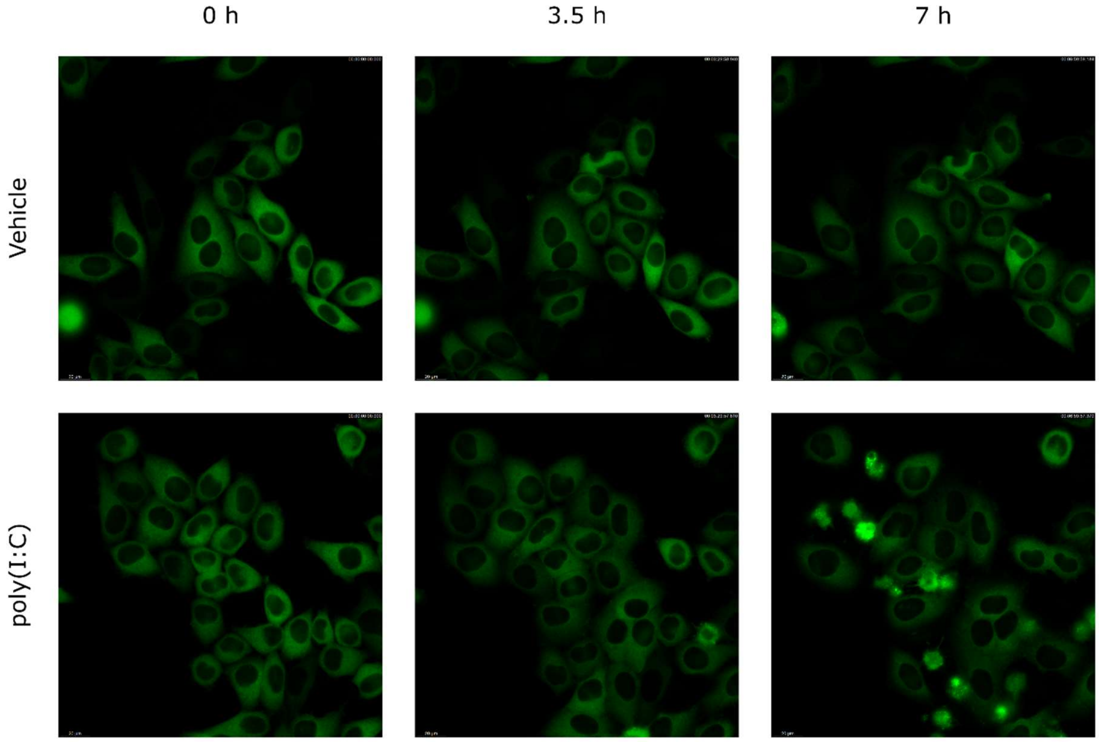

Minor points:

1. pg5 line 17, the aa range listed for both mutants is the same, please correct this
2. pg5 line 24, remove 'only' after PYD
3. pg 6 line 13, change underwent to undergone

Response: We thank the reviewer for critical reading of our manuscript and have corrected these errors accordingly.

4. pg 6 line 19 - what do the authors mean by 'early diverged from this cluster'? More rigorous phylogenetic analysis is required to support such statements.

Response: We thank the reviewer for this comment. We have expanded the sentence as it was not clear what we meant, we now include the divergence that must have occurred when the *Homo sapiens* separated from the *Pan paniscus/Pan troglodytes* branch (page 6, lines 27-29).

References

Brinkschulte, Rebecca; Fußhöller, David M.; Hoss, Florian; Rodríguez-Alcázar, Juan F.; Lauterbach, Mario A.; Kolbe, Carl-Christian et al. (2022): ATP-binding and hydrolysis of human NLRP3. In: *Communications biology* 5 (1), S. 1176. DOI: 10.1038/s42003-022-04120-2.

Chen, Jueqi; Chen, Zhijian J. (2018): PtdIns4P on dispersed trans-Golgi network mediates NLRP3 inflammasome activation. In: *Nature* 564 (7734), S. 71–76. DOI: 10.1038/s41586-018-0761-3.

Dekker, Carien; Mattes, Henri; Wright, Michael; Boettcher, Andreas; Hinniger, Alexandra; Hughes, Nicola et al. (2021): Crystal Structure of NLRP3 NACHT Domain With an Inhibitor Defines Mechanism of Inflammasome Inhibition. In: *Journal of molecular biology* 433 (24), S. 167309. DOI: 10.1016/j.jmb.2021.167309.

He, Wan-ting; Wan, Haoqiang; Hu, Lichen; Chen, Pengda; Wang, Xin; Huang, Zhe et al. (2015): Gasdermin D is an executor of pyroptosis and required for interleukin-1β secretion. In: *Cell research* 25 (12), S. 1285–1298. DOI: 10.1038/cr.2015.139.

Hochheiser, Inga V.; Geyer, Matthias (2023): Chapter 14 - Structural aspects of inflammasomes forming NOD-like receptors. In: Pablo Pelegrin (Hg.): Inflammasome Biology: Academic Press, S. 215–233. Online verfügbar unter https://www.sciencedirect.com/science/article/pii/B9780323918022000220.

Lautz, Katja; Damm, Anna; Menning, Maureen; Wenger, Julia; Adam, Alexander C.; Zigrino, Paola et al. (2012): NLRP10 enhances Shigella-induced pro-inflammatory responses. In: *Cellular microbiology* 14 (10), S. 1568–1583. DOI: 10.1111/j.1462-5822.2012.01822.x.

Próchnicki, Tomasz (2021): Regulation of inflammasome activation: roles for phospholipase C and mitochondria. Dissertation. Universitäts- und Landesbibliothek Bonn, Bonn. Online verfügbar unter https://bonndoc.ulb.uni-bonn.de/xmlui/handle/20.500.11811/9261, zuletzt geprüft am 31.03.2026.

Próchnicki, Tomasz; Vasconcelos, Matilde B.; Robinson, Kim S.; Mangan, Matthew S. J.; Graaf, Dennis de; Shkarina, Kateryna et al. (2023): Mitochondrial damage activates the NLRP10 inflammasome. In: *Nature immunology* 24 (4), S. 595–603. DOI: 10.1038/s41590-023-01451-y.

Su, Ming-Yuan; Kuo, Chiao-I; Chang, Chi-Fon; Chang, Chung-I (2013): Three-dimensional structure of human NLRP10/PYNOD pyrin domain reveals a homotypic interaction site distinct from its mouse homologue. In: *PloS one* 8 (7), e67843. DOI: 10.1371/journal.pone.0067843.

Wendler, Petra; Ciniawsky, Susanne; Kock, Malte; Kube, Sebastian (2012): Structure and function of the AAA+ nucleotide binding pocket. In: *Biochimica et biophysica acta* 1823 (1), S. 2–14. DOI: 10.1016/j.bbamcr.2011.06.014.

Zhang, Zhiqiang; Wang, Haoxiang; Huang, Yumeng; Zhu, Liming; Wang, Xuanyu; Du, Yan et al. (2025): Heavy ion radiation induces pyroptosis in skin keratinocytes through a mitogen-activated protein kinase signaling pathway. In: *International journal of radiation biology* 101 (11), S. 1142–1150. DOI: 10.1080/09553002.2025.2561813.

Zheng, Danping; Mohapatra, Gayatree; Kern, Lara; He, Yiming; Shmueli, Merav D.; Valdés-Mas, Rafael et al. (2023): Epithelial Nlrp10 inflammasome mediates protection against intestinal autoinflammation. In: *Nature immunology* 24 (4), S. 585–594. DOI: 10.1038/s41590-023-01450-z.

1st Revision - Editorial Decision **11th May 2026**

Dear Dr. Voss,

Thank you for the submission of your revised manuscript to our editorial offices. I have now received the reports from the three referees that were asked to re-evaluate the study, you will find below. As you will see, the referees now support the publication of the study in EMBO reports. However, all three referees have remaining minor concerns and/or suggestions to improve the manuscript, I ask you to address in a final revised version. Please also provide a final p-b-p-response to these points and the editorial requests below.

Editorial requests:

- Please order the manuscript sections like this, using only these names:
Title page - Abstract - Keywords - Introduction - Results & Discussion - Methods - Data availability section - Acknowledgements (please include here all the funding information) - Disclosure and Competing Interests Statement - References - Figure legends - Expanded View Figure legends

Thus, please provide a dedicated section named 'Data availability' and state there that no primary datasets have been generated and deposited for this study.

- Please remove the running title from the manuscript title page.

- Please add scale bars of similar style and thickness to all microscopic images (main, EV and Appendix figures), using clearly visible black or white bars (depending on the background). Please place these in the lower right corner of the images themselves. Please do not write on or near the bars in the image but define the size in the respective figure legend. Presently, most scale bars are rather thin, and some have text nearby (that is hard to read - e.g. for panel 4A). Please improve.

- Please check again that the number "n" for how many independent experiments were performed, their nature (biological versus technical replicates), the bars and error bars (e.g. SEM, SD) and the test used to calculate p-values is indicated in the respective figure legends (main and EV figures). Please also check that all the p-values are explained in the legend, and that these fit to those shown in the figure. Please provide statistical testing where applicable. Please avoid the phrase 'independent experiment' but clearly state if these were biological or technical replicates. Please also indicate (e.g. with n.s.) if testing was performed, but the differences are not significant. In case n=2, please show the data as separate datapoints without error bars and statistics. See also:

https://link.springer.com/journal/44319/submission-guidelines#cms-Figure-and-data-presentation

If n<5, please show single datapoints for diagrams. Moreover:
- Please note that the legends for figures 2, 3 are not provided in the sequential manner. This needs to be rectified.
- Please note that the exact p values are not provided in the legends of figures 3B,F,J.
- Please note that information related to n is missing in the legends of EV-1E; EV-4B.
- Please note that the error bars are not defined in the legends of figures EV-1E; EV-4B.

- Please use our reference format:
https://link.springer.com/journal/44319/submission-guidelines#cms-Reference-guidelines

Et al needs to be used after 10 author names; DOIs should only be used for preprints and datasets that have not been published yet.

- Please remove the author names and affiliations from the Appendix Title page. It is sufficient to state "Appendix for ..." followed by the title and then the table of contents.

- All Materials and Methods need to be described in the main text using our 'Structured Methods' format, which is required for all research articles. According to this format, the Methods section should include a Reagents and Tools Table (listing key reagents, experimental models, software, and relevant equipment and including their sources and relevant identifiers), uploaded as separate file, and a Methods section in which we encourage the authors to describe their methods using a step-by-step protocol format with bullet points, to facilitate the adoption of the methodologies across labs. More information on how to adhere to this format as well as downloadable templates (.doc) for the Reagents and Tools Table can be found in our author guidelines (section 'Structured Methods'):

https://link.springer.com/journal/44319/submission-guidelines#cms-Manuscript-organisation-and-formatting

Please add primer or antibody information directly to the Reagents and Tools table.

- Please confirm that for all Western blot panels shown the loading control was run on the same gel as the other proteins detected. Please note that we discourage comparisons between samples on different gels/blots, even if the samples derive from one experiment, as confounding factors reduce comparability. If unavoidable, the figure legend must state that the samples derive from the same experiment and that gels/blots were processed in parallel. If a 'representative' loading control is shown for multiple gels/blots, the intra-gel controls should be shown in the source data files, and the figure legends should describe the data displayed accurately. See our author guidelines:

https://link.springer.com/journal/44319/submission-guidelines#cms-Figure-and-data-presentation (section 'Electrophoretic gels and blots').

- Please make sure that all the funding information is also entered into the online submission system and that it is complete and similar to the one in the acknowledgement section of the manuscript text file. Presently, the grants EFRE EU fund (grant no. 2172959) and grants from the Funding Programme Open Access Publishing of University of Hohenheim are missing in the submission system. Please check.

- Thanks for providing the source data. The source data folder for figure EV5 cannot be unzipped. Please check and provide an unzippable folder.

- It seems some images shown in Figs. 1A or 1B are also shown in Figs. EV4C or EV5C. Please check. If this reuse is intentional, please clearly indicate this in the respective figure legends.

- For Fig. EV1A GFP (left panel) the image seems empty (no background signal). This might be the case, as GFP is not expressed (please check). Nevertheless, from the source data it is not clear what part of the source data blot is shown in the figure. Please mark the parts of the images shown in the source data that have been used for putting together Fig. EV1A for all four sub-panels.

In addition, I would need from you uploaded separately:
- a schematic summary figure as separate file that provides a sketch of the major findings (not a data image) in jpeg or tiff format (with the exact width of 550 pixels and the exact height of 300 pixels) that can be used as a visual synopsis on our website.

I look forward to seeing the further revised version of your manuscript when it is ready. Please let me know if you have questions regarding the revision.

Best,

Achim Breiling
Senior Editor
EMBO Reports

Referee #1:

I would like to thank the authors for their work in addressing concerns and adapting the manuscript. I think the manuscript is close to the point of being ready for publication.

My final points are minor disagreements which could be adapted by changes to the text. If the authors maintain that the manuscript should not be further changed or that I have misunderstood comments, then I defer final points to the editor. I will gently suggest that there is a slight language barrier and would not want this to affect publication. Language issues in the text should not be beyond pre-publication proofreading.

1) In the NLRP10 visualisations I am not seeing circular or ring-like structures for the most part. (e.g. 1A/C, 2B). The inset example in 4C appears the most ring-like but I do not think it is representative. The argument as I understand it is that NLRP10 oligomers are recruited to lipid droplets and I don't think it is important or expected that they decorate them in a circular or ring-like fashion. I would suggest that the authors describe the shape differently, possibly by using the calculated sphericity in the main text.

2) The lack of activity in HaCaT cells is not overstated in the text and as such it is suitable for publication. However, I do not view poly(I:C) as a typical positive control for pyroptosis. More likely to me is that TLR3->Necroptosis or MAVS->Apoptosis->Secondary necrosis eventually results in some IL-18 entering the supernatant. I did not feel the provided references backed up this point (He et al. did not use HaCaT as far as I could tell). NLRP1 activation for example would be a more standard positive control for pyroptosis. IL-18 release is measured at 20 minutes while HaCaT oligomerisation is shown at 60 minutes. Oligomerisation is shown in the other cell models at earlier time points - acknowledgement in the text that this is also true for

HaCaT cells should be included.

3) There are numerous points in the text where a non-significant effect is still described as, for example, an "insignificant increase". The authors note that they include the significance values so that readers can judge for themselves. In my opinion only significant changes should be referred to in the text. This may well be a comment for the Editor to judge on. One example is Page 6 Line 6 (0.2071) and another is Page 7 Line 10 (0.4748).

Good luck with any final changes.

Referee #2:

The authors have addressed my comments and criticism with additional experiments.
There are only some small issues that I would ask them to correct before publication:

-The authors consistently refer to the clustering of NLRP10 at Lipid droplets as 'oligomerization'. In my opinion, the study does not provide sufficient evidence to make this claim in cells. It is true that the data obtained with the purified protein suggest that the protein forms oligomers, but as the authors have not formally checked this in cells using native gel or cosslinking assays, it is still possible that NLRP10 is just recruited to LDs without oligomerizing.
I would thus suggest to tone down to claim and use words like 're-localization' 'translocation' or 'clustering'.

-There are some claims that seem not to be supported by data. In the rebuttal the authors write:
"Both, full-length NLRP10 and NLRP10del584 are recruited by these ACS specks, but not NLRP10delPYD." But I couldn't find the data.

"In the live cell experiments we have not quantified ASC specks, as they were not dependent on expression of NLRP10 (also eGFP cells show ASC specks)." same for this claim.

-page 10, lines 27-36: I would slightly tone down the claim regarding EV6B. To me this experiment cannot completely exclude that ASC speck formation was initiated by low levels of active NLRP10 (which might have been below the detection level for microscopy), and that additional NLRP10 was recruited only afterwards when the ASC speck formed. But I agree that are some clear differences in the biology of NLRP10 and NLRP3.

Referee #3:

The authors have sufficiently addressed my concerns. I would recommend using a colourblind-friend palette for microscopy images (change red to magenta or green to cyan).

UNIVERSITÄT HOHENHEIM

Universität Hohenheim (180b) | 70593 Stuttgart

Faculty of Natural Sciences
Institute of Nutritional Medicine
Department of Immunology

Prof. Dr. rer.nat. Thomas A. Kufer
Chair of Immunology

T +49 711 459 24850
F +49 711 459 24855
E thomas.kufer@uni-hohenheim.de
https://immu.uni-hohenheim.de/en

EMBO Reports

Achim Breiling
Senior Editor

03.06.2026

Dear Dr. Breiling,

Thank you for handling of our manuscript "C-terminal lysine residues localise NLRP10 at lipid droplets and govern NLRP10 oligomer formation" and the positive feed-back. We also want to thank the reviewers for their constructive criticism that helped us to further improve the manuscript.

Attached please find a point-by-point response to your requests on final formatting requirements and to the last reviewer queries. All points were carefully addressed and included in the final version of the manuscript.

All authors agreed on submission of the manuscript and declare to have no conflict of interest.

Thank you once again for consideration of our manuscript.

Sincerely,

UNIVERSITÄT HOHENHEIM
Institut für Ernährungsmedizin
Fg. Immunologie
Fruwirthstr. 12
70599 Stuttgart
https://immu.uni-hohenheim.de/

BADEN-WÜRTTEMBERGISCHE BANK
IBAN DE20 6005 0101 0002 5601 08
BIC-Code SOLADEST600
UST-ID DE 147 794 207

ANFAHRT
Stadtbahn
U3, Plieningen (Universität Hohenheim)
Bus
65, 70, 73, 74, 75, 76, 79

Zertifikat seit 2004 audit familiengerechte hochschule

Point-by-point response

Editorial queries:

- Please order the manuscript sections like this, using only these names:
Title page - Abstract - Keywords - Introduction - Results & Discussion - Methods - Data availability section - Acknowledgements (please include here all the funding information) - Disclosure and Competing Interests Statement - References - Figure legends - Expanded View Figure legends
Thus, please provide a dedicated section named 'Data availability' and state there that no primary datasets have been generated and deposited for this study.
- Please remove the running title from the manuscript title page.
We have removed running title and changed all according to the request.

- Please add scale bars of similar style and thickness to all microscopic images (main, EV and Appendix figures), using clearly visible black or white bars (depending on the background). Please place these in the lower right corner of the images themselves. Please do not write on or near the bars in the image but define the size in the respective figure legend. Presently, most scale bars are rather thin, and some have text nearby (that is hard to read - e.g. for panel 4A). Please improve.
We have adapted the figures accordingly; scale bars are now thicker and Fig 4A scale bare is different. Concerning Fig 4A we cannot remove the scale bar because the scale bar is embedded in the figure and the images were processes at the core facility and exported with the scale bar. Thus, we had to crop the scale bar from the figure, and therefore we moved the original file to the source data, which we updated.

- Please check again that the number "n" for how many independent experiments were performed, their nature (biological versus technical replicates), the bars and error bars (e.g. SEM, SD) and the test used to calculate p-values is indicated in the respective figure legends (main and EV figures). Please also check that all the p-values are explained in the legend, and that these fit to those shown in the figure. Please provide statistical testing where applicable. Please avoid the phrase 'independent experiment' but clearly state if these were biological or technical replicates. Please also indicate (e.g. with n.s.) if testing was performed, but the differences are not significant. In case n=2, please show the data as separate datapoints without error bars and statistics. See also:

https://link.springer.com/journal/44319/submission-guidelines#cms-Figure-and-data-presentation

If n<5, please show single datapoints for diagrams. Moreover:
We have checked the legends and do not refer to as “independent” experiments. In the Method section we state that “N” always refers to biological replicates, no technical replicates are represented in graphs, technical replicates were pooled before.
- Please note that the legends for figures 2, 3 are not provided in the sequential manner. This needs to be rectified.

- Please note that the exact p values are not provided in the legends of figures 3B,F,J.
- Please note that information related to n is missing in the legends of EV-1E; EV-4B.
- Please note that the error bars are not defined in the legends of figures EV-1E; EV-4B.

All this information has been added to the final version.

- Please use our reference format:
https://link.springer.com/journal/44319/submission-guidelines#cms-Reference-guidelines

Et al needs to be used after 10 author names; DOIs should only be used for preprints and datasets that have not been published yet.
We have updated the reference style to EMBO Reports format.

- Please remove the author names and affiliations from the Appendix Title page. It is sufficient to state "Appendix for ..." followed by the title and then the table of contents.
We have updated the Appendix title page.

- All Materials and Methods need to be described in the main text using our 'Structured Methods' format, which is required for all research articles. According to this format, the Methods section should include a Reagents and Tools Table (listing key reagents, experimental models, software, and relevant equipment and including their sources and relevant identifiers), uploaded as separate file, and a Methods section in which we encourage the authors to describe their methods using a step-by-step protocol format with bullet points, to facilitate the adoption of the methodologies across labs. More information on how to adhere to this format as well as downloadable templates (.doc) for the Reagents and Tools Table can be found in our author guidelines (section 'Structured Methods'):

https://link.springer.com/journal/44319/submission-guidelines#cms-Manuscript-organisation-and-formatting

Please add primer or antibody information directly to the Reagents and Tools table.
We have now included the table.

- Please confirm that for all Western blot panels shown the loading control was run on the same gel as the other proteins detected. Please note that we discourage comparisons between samples on different gels/blots, even if the samples derive from one experiment, as confounding factors reduce comparability. If unavoidable, the figure legend must state that the samples derive from the same experiment and that gels/blots were processed in parallel. If a 'representative' loading control is shown for multiple gels/blots, the intra-gel controls should be shown in the source data files, and the figure legends should describe the data displayed accurately. See our author guidelines:

https://link.springer.com/journal/44319/submission-guidelines#cms-Figure-and-data-presentation (section 'Electrophoretic gels and blots').
Loading control was always detected on the same membrane, this has been added in the text to clarify this.

- Please make sure that all the funding information is also entered into the online submission system and that it is complete and similar to the one in the acknowledgement section of the manuscript text file. Presently, the grants EFRE EU fund (grant no. 2172959) and grants from the Funding Programme Open Access Publishing of University of Hohenheim are missing in the submission system. Please check.
We added the missing information in the online submission system.

- Thanks for providing the source data. The source data folder for figure EV5 cannot be unzipped. Please check and provide an unzippable folder.
Unfortunately, the file was compromised, we apologize for any inconvenience. We now provide a new file, which should open. Additionally, we updated Source data files for Fig 4 and EV1.

- It seems some images shown in Figs. 1A or 1B are also shown in Figs. EV4C or EV5C. Please check. If this reuse is intentional, please clearly indicate this in the respective figure legends.
Indeed, the reuse is intentional. We now clearly state this in the expanded view figure legend.

- For Fig. EV1A GFP (left panel) the image seems empty (no background signal). This might be the case, as GFP is not expressed (please check). Nevertheless, from the source data it is not clear what part of the source data blot is shown in the figure. Please mark the parts of the images shown in the source data that have been used for putting together Fig. EV1A for all four sub-panels.
We apologize that the source data was not clear. We reorganized the presentation. In this file we also explain the apparent lack of signal in the left panel. Changing contrast or brightness allows to visualize background pixels to prove that this was a developed membrane. The new source data file has been uploaded in the system.

In addition, I would need from you uploaded separately:
- a schematic summary figure as separate file that provides a sketch of the major findings (not a data image) in jpeg or tiff format (with the exact width of 550 pixels and the exact height of 300 pixels) that can be used as a visual synopsis on our website.
We have prepared such a file and uploaded it in the correct format.

During revision we realised that the method section for the generation of the CRISPR knockout cells was missing. This part, with the plasmid information, has been added to the revised manuscript.

Referee #1:

I would like to thank the authors for their work in addressing concerns and adapting the manuscript. I think the manuscript is close to the point of being ready for publication.

My final points are minor disagreements which could be adapted by changes to the text. If the authors maintain that the manuscript should not be further changed or that I have misunderstood comments, then I defer final points to the editor. I will gently suggest that there is a slight language barrier and would not want this to affect publication. Language issues in the text should not be beyond pre-publication proofreading.

We want to thank the reviewer for her/his commitment and the overall positive evaluation of the work.

1) In the NLRP10 visualisations I am not seeing circular or ring-like structures for the most part. (e.g. 1A/C, 2B). The inset example in 4C appears the most ring-like but I do not think it is representative. The argument as I understand it is that NLRP10 oligomers are recruited to lipid droplets and I don't think it is important or expected that they decorate them in a circular or ring-like fashion. I would suggest that the authors describe the shape differently, possibly by using the calculated sphericity in the main text.
We thank the reviewer for this suggestion. In most experiments, we observed ring-like structures. However, we fully agree that this term is somewhat unprecise. Following the reviewers' suggestions, that the sphericity might be the best descriptor for these structures we now refer to these as "spherical" in the revised version (page 4 lines 23 and 24; page 8 line 32).

2) The lack of activity in HaCaT cells is not overstated in the text and as such it is suitable for publication. However, I do not view poly(I:C) as a typical positive control for pyroptosis. More likely to me is that TLR3->Necroptosis or MAVS->Apoptosis->Secondary necrosis eventually results in some IL-18 entering the supernatant. I did not feel the provided references backed up this point (He et al. did not use HaCaT as far as I could tell). NLRP1 activation for example would be a more standard positive control for pyroptosis. IL-18 release is measured at 20 minutes while HaCaT oligomerisation is shown at 60 minutes. Oligomerisation is shown in the other cell models at earlier time points - acknowledgement in the text that this is also true for HaCaT cells should be included.
We principally agree with the reviewer. Unfortunately, we included a wrong citation, indeed it should be (Wang *et al,* 2024). Additionally (Prieux *et al,* 2022) showed that HaCaT cells are capable of releasing IL-18. We fully agree that poly(I:C) is not a good positive control for inflammasome activation. However, in our hands it robustly leads to release of IL-18, which still is a suited control for the functionality of our ELISA and to make the fact that HaCaT cells can produce IL-18. We did not further elaborate on this, because we did not observe IL-18 release upon oligomerisation of NLRP10. Concerning the time-points shown, we agree that it might appear that

we measure IL-18 release earlier than oligomer formation. Below, the reviewer might appreciate that we already observe oligomerisation at earlier time-points (exemplified by the shown 30 min frame). For the collection of the supernatant for measurement of IL-18 release we observed that prolonged *m*-3M3FBS treatement lead to an increase of IL-18 into the supernatant, which we also observed in the knockout cells. We assume that this might represent unprocessed IL-18, as the cells' cytosol is quickly released into the supernatant.

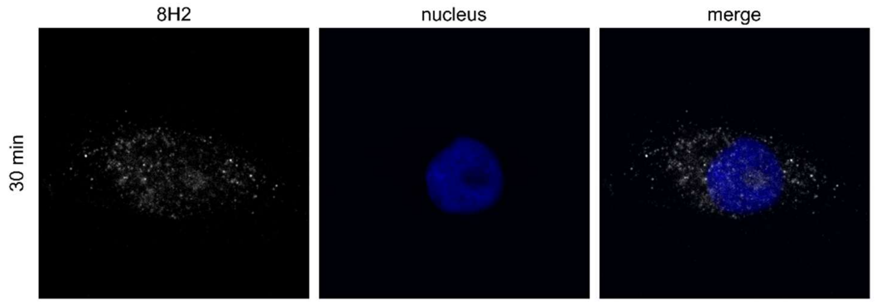

3) There are numerous points in the text where a non-significant effect is still described as, for example, an "insignificant increase". The authors note that they include the significance values so that readers can judge for themselves. In my opinion only significant changes should be referred to in the text. This may well be a comment for the Editor to judge on. One example is Page 6 Line 6 (0.2071) and another is Page 7 Line 10 (0.4748).
We understand the concerns of the reviewer. We think that appropriate description of the data also includes to mention trends to best describe the data. However, we understand that can put too much emphasis on insignificant data. To account for this, we edited this part to reduce the strength of the sentence (page 7 line 10 (now: page 7 line 18) "while deletion of the mouse tail did not significantly (1.8 ± 0.7 %; p = 0.4748) affect protein stability").

Referee #2:

The authors have addressed my comments and criticism with additional experiments.
There are only some small issues that I would ask them to correct before publication:
We want to thank the reviewer for her/his commitment and the overall positive evaluation of the work.

-The authors consistently refer to the clustering of NLRP10 at Lipid droplets as 'oligomerization'. In my opinion, the study does not provide sufficient evidence to make this claim in cells. It is true that the data obtained with the purified protein suggest that the protein forms oligomers, but as the authors have not formally checked this in cells using native gel or cosslinking assays, it is still possible that NLRP10 is just recruited to LDs without oligomerizing.

I would thus suggest to tone down to claim and use words like 're-localization' 'translocation' or 'clustering'.
We thank the reviewer for his/her suggestion, however we politely disagree with the reviewers' view. The nature of NLRPs is interaction with each other, hydrolysis of ATP can only be facilitated as multimers, for the NLRP10Δ584 ATP hydrolysis is almost completely blunted and no oligomerisation or clustering in response to *m*-3M3FBS can be observed. SEC-MALS measurements (Fig. EV2H) shows that the tail deletion block multimer formation. Together with the lack of "re-localisation" of the NLRP10 K0 mutant, the data strongly suggests oligomer formation at lipid droplets, even though we technically do not show these oligomers. The other reviewers agreed with the use of oligomerisation. We hope that this reviewer can follow our argumentation.

-There are some claims that seem not to be supported by data. In the rebuttal the authors write: "Both, full-length NLRP10 and NLRP10del584 are recruited by these ACS specks, but not NLRP10delPYD." But I couldn't find the data.
The reviewer is right and we apologise that we forgot to include this data. Below the reviewer can see the NLRP10Δ584/ASC speck.

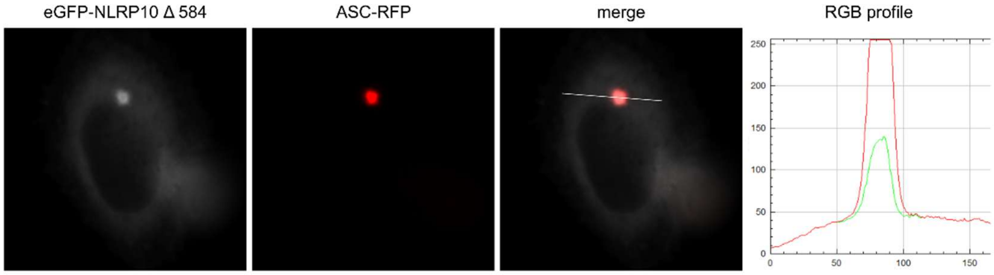

"In the live cell experiments we have not quantified ASC specks, as they were not dependent on expression of NLRP10 (also eGFP cells show ASC specks)." same for this claim.
We are sorry to make the claim without supporting figures. Due to space constraints we provide the figure here for the reviewer.

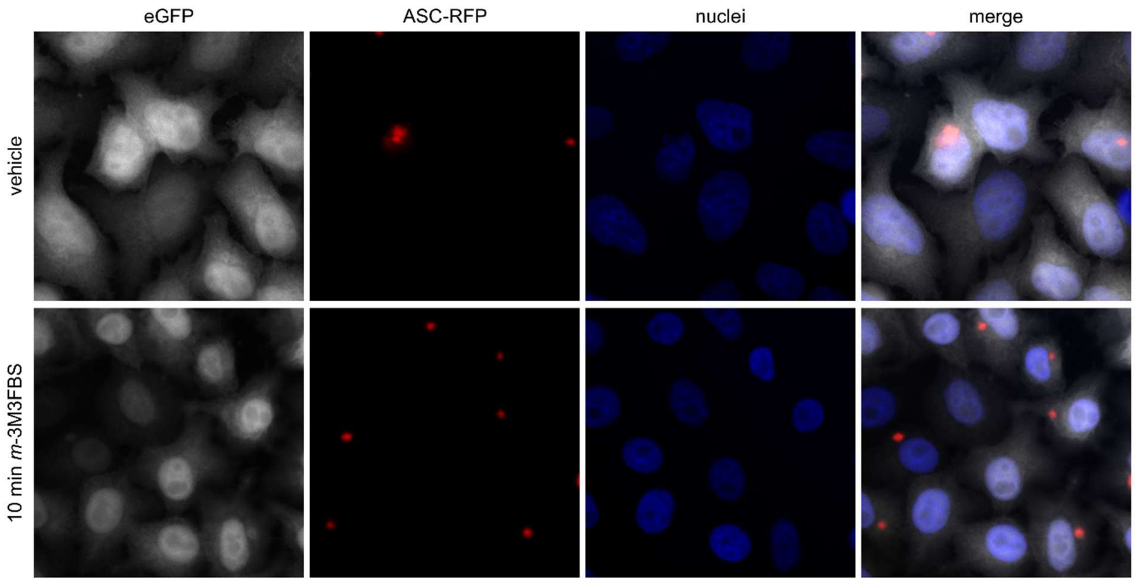

-page 10, lines 27-36: I would slightly tone down the claim regarding EV6B. To me this experiment cannot completely exclude that ASC speck formation was initiated by low levels of active NLRP10 (which might have been below the detection level for microscopy), and that additional NLRP10 was recruited only afterwards when the ASC speck formed. But I agree that are some clear differences in the biology of NLRP10 and NLRP3.

We thank the reviewer for raising this point. We agree that additional experimental lines would be need to fully support this claim, albeit we are confident that speck formation and oligomerisation are not dependent on each other in the used system. To account for shortcomings of the system and for eventual undetectable oligomerisation of NLRP10, we adapted the sentence accordingly: "ASC aggregated prior visible NLRP10 recruitment, suggesting that NLRP10 is not the seed for ASC filament formation in this cell system" (page 10 lines 34-35).

Referee #3:

The authors have sufficiently addressed my concerns. I would recommend using a colourblind-friend palette for microscopy images (change red to magenta or green to cyan).

First, we want to thank the reviewer for her/his commitment and the positive evaluation of the work.

We also thank the reviewer for pointing out that we have not used appropriate colours in two supplementary figures. This was now changed in the final revision (e.g.: Fig EV1 C, eGFP-NLRP10 signal changed from green to white).

References

Prieux R, Ferrara F, Cervellati F, Guiotto A, Benedusi M, Valacchi G (2022) Inflammasome involvement in CS-induced damage in HaCaT keratinocytes. *In Vitro Cell Dev Biol Anim* 58: 335–348

Wang T, Yazdi AS, Panayotova-Dimitrova D (2024) Comparison of Different Keratinocyte Cell Line Models for Analysis of NLRP1 Inflammasome Activation. *Biomolecules* 14

Dr. Timo-Daniel Voss
University of Hohenheim
Department of Immunology
Institute of Nutritional Medicine
Fruwirth Str. 12
Stuttgart 70599
Germany

Dear Dr. Voss,

Thank you for the submission of your final revised manuscript to our editorial offices. I now went through this and your final point-by-point-response and consider the remaining points of the referees and the editorial requests as adequately addressed.

I am thus very pleased to accept your manuscript for publication in the next available issue of EMBO reports. Thank you for your contribution to our journal.

Your manuscript will be processed for publication by EMBO Press. It will be copy edited and you will receive page proofs prior to publication. Please note that you will be contacted by Springer Nature Author Services to complete licensing and payment information.

You may qualify for financial assistance for your publication charges - either via a Springer Nature fully open access agreement or an EMBO initiative. Check your eligibility: https://link.springer.com/journal/44319/how-to-publish-with-us

Should you be planning a Press Release on your article, please notify the production team via this form in order to coordinate publication and release dates: https://forms.cloud.microsoft/e/eH4A8JVP6g

If you have any questions, please do not hesitate to contact the Editorial Office. Thank you for your contribution to EMBO Reports.

Yours sincerely,

Achim Breiling
Senior Editor
EMBO Reports

>>> Please note that it is EMBO Reports policy for the transcript of the editorial process (containing referee reports and your response letter) to be published as an online supplement to each paper. If you do NOT want this, you will need to inform the Editorial Office via email immediately. More information is available here: https://link.springer.com/partners/embo-press/editorial-policies#Peer%20review

EMBO Press Author Checklist

Corresponding Author Name: Timo-Daniel Voss
Journal Submitted to: EMBO Reports
Manuscript Number: EMBOR-2025-63425-T

USEFUL LINKS FOR COMPLETING THIS FORM
The EMBO Journal - Author Guidelines
EMBO Reports - Author Guidelines
Molecular Systems Biology - Author Guidelines
EMBO Molecular Medicine - Author Guidelines

Reporting Checklist for Life Science Articles (updated January
This checklist is adapted from Materials Design Analysis Reporting (MDAR) Checklist for Authors. MDAR establishes a minimum set of requirements in transparent reporting in the life sciences (see Statement of Task: 10.31222/osf.io/9sm4x). Please follow the journal's guidelines in preparing your
Please note that a copy of this checklist will be published alongside your article.

Abridged guidelines for figures
1. Data
The data shown in figures should satisfy the following conditions:
- the data were obtained and processed according to the field's best practice and are presented to reflect the results of the experiments in an accurate and unbiased manner.
- ideally, figure panels should include only measurements that are directly comparable to each other and obtained with the same assay.
- plots include clearly labeled error bars for independent experiments and sample sizes. Unless justified, error bars should not be shown for technical
- if n<5, the individual data points from each experiment should be plotted. Any statistical test employed should be justified.
- Source Data should be included to report the data underlying figures according to the guidelines set out in the authorship guidelines on Data

2. Captions
Each figure caption should contain the following information, for each panel where they are relevant:
- a specification of the experimental system investigated (eg cell line, species name).
- the assay(s) and method(s) used to carry out the reported observations and measurements.
- an explicit mention of the biological and chemical entity(ies) that are being measured.
- an explicit mention of the biological and chemical entity(ies) that are altered/varied/perturbed in a controlled manner.
- the exact sample size (n) for each experimental group/condition, given as a number, not a range;
- a description of the sample collection allowing the reader to understand whether the samples represent technical or biological replicates (including how many animals, litters, cultures, etc.).
- a statement of how many times the experiment shown was independently replicated in the laboratory.
- definitions of statistical methods and measures:
  - common tests, such as t-test (please specify whether paired vs. unpaired), simple $\chi 2$ tests, Wilcoxon and Mann-Whitney tests, can be unambiguously identified by name only, but more complex techniques should be described in the methods section;
  - are tests one-sided or two-sided?
  - are there adjustments for multiple comparisons?
  - exact statistical test results, e.g., P values = x but not P values < x;
  - definition of 'center values' as median or average;
  - definition of error bars as s.d. or s.e.m.

Please complete ALL of the questions below.
Select "Not Applicable" only when the requested information is not relevant for your study.

Materials

Newly Created Materials	Information included in the manuscript?	In which section is the information available? (Reagents and Tools Table, Materials and Methods, Figures, Data Availability Section)
New materials and reagents need to be available; do any restrictions apply?	Not Applicable	

Antibodies	Information included in the manuscript?	In which section is the information available? (Reagents and Tools Table, Materials and Methods, Figures, Data Availability Section)
For **antibodies** provide the following information:<br>- Commercial antibodies: RRID (if possible) or supplier name, catalogue number and or/clone number<br>- Non-commercial: RRID or citation	Yes	Materials and Methods

DNA and RNA sequences	Information included in the manuscript?	In which section is the information available? (Reagents and Tools Table, Materials and Methods, Figures, Data Availability Section)
**Short novel DNA or RNA including primers, probes**: provide the sequences.	Yes	Appendix

Cell materials	Information included in the manuscript?	In which section is the information available? (Reagents and Tools Table, Materials and Methods, Figures, Data Availability Section)
**Cell lines:** Provide species information, strain. Provide accession number in repository **OR** supplier name, catalog number, clone number, and/**OR** RRID.	Yes	Materials and Methods Page 14
**Primary cultures:** Provide species, strain, sex of origin, genetic modification status.	Not Applicable	
Report if the cell lines were recently **authenticated** (e.g., by STR profiling) and tested for mycoplasma contamination.	Yes	Page 14

Experimental animals	Information included in the manuscript?	In which section is the information available? (Reagents and Tools Table, Materials and Methods, Figures, Data Availability Section)
**Laboratory animals or Model organisms:** Provide species, strain, sex, age, genetic modification status. Provide accession number in repository **OR** supplier name, catalog number, clone number, **OR** RRID.	Not Applicable	
**Animal observed in or captured from the field:** Provide species, sex, and age where possible.	Not Applicable	
Please detail **housing and husbandry conditions**.	Not Applicable	

Plants and microbes	Information included in the manuscript?	In which section is the information available? (Reagents and Tools Table, Materials and Methods, Figures, Data Availability Section)
**Plants:** provide species and strain, ecotype and cultivar where relevant, unique accession number if available, and source (including location for collected wild specimens).	Not Applicable	
**Microbes:** provide species and strain, unique accession number if available, and source.	Not Applicable	

Human research participants	Information included in the manuscript?	In which section is the information available? (Reagents and Tools Table, Materials and Methods, Figures, Data Availability Section)
If collected and within the bounds of privacy constraints report on age, sex and gender or ethnicity for all study participants.	Not Applicable	

Core facilities	Information included in the manuscript?	In which section is the information available? (Reagents and Tools Table, Materials and Methods, Figures, Data Availability Section)
If your work benefited from core facilities, was their service mentioned in the acknowledgments section?	Yes	Materials and Methods, Acknowledgments

Design

Study protocol	Information included in the manuscript?	In which section is the information available? (Reagents and Tools Table, Materials and Methods, Figures, Data Availability Section)
If study protocol has been **pre-registered, provide DOI in the manuscript**. For clinical trials, provide the trial registration number **OR** cite DOI.	Not Applicable	
Report the **clinical trial registration number** (at ClinicalTrials.gov or equivalent), where applicable.	Not Applicable	

Laboratory protocol	Information included in the manuscript?	In which section is the information available? (Reagents and Tools Table, Materials and Methods, Figures, Data Availability Section)
Provide DOI OR other citation details if **external detailed step-by-step protocols** are available.	Not Applicable	

Experimental study design and statistics	Information included in the manuscript?	In which section is the information available? (Reagents and Tools Table, Materials and Methods, Figures, Data Availability Section)
Include a statement about **sample size** estimate even if no statistical methods were used.	Yes	Figure Legends
Were any steps taken to minimize the effects of subjective bias when allocating animals/samples to treatment (e.g. **randomization procedure**)? If yes, have they been described?	Not Applicable	
Include a statement about **blinding** even if no blinding was done.	Not Applicable	
Describe **inclusion/exclusion criteria** if samples or animals were excluded from the analysis. Were the criteria pre-established?<br><br>If sample or data points were omitted from analysis, report if this was due to attrition or intentional exclusion and provide justification.	Not Applicable	
For every figure, are **statistical tests** justified as appropriate? Do the data meet the assumptions of the tests (e.g., normal distribution)? Describe any methods used to assess it. Is there an estimate of variation within each group of data? Is the variance similar between the groups that are being statistically compared?	Yes	Figure Legends and Materials and Methods

Sample definition and in-laboratory replication	Information included in the manuscript?	In which section is the information available? (Reagents and Tools Table, Materials and Methods, Figures, Data Availability Section)
In the figure legends: state number of times the experiment was **replicated** in laboratory.	Yes	Figure Legends
In the figure legends: define whether data describe **technical or biological replicates**.	Yes	Figure Legends

Ethics

Ethics	Information included in the manuscript?	In which section is the information available? (Reagents and Tools Table, Materials and Methods, Figures, Data Availability Section)
Studies involving **human participants**: State details of **authority granting ethics approval** (IRB or equivalent committee(s), provide reference number for approval.	Not Applicable	
Studies involving **human participants**: Include a statement confirming that **informed consent** was obtained from all subjects and that the experiments conformed to the principles set out in the WMA Declaration of Helsinki and the Department of Health and Human Services Belmont Report.	Not Applicable	
Studies involving **human participants:** For publication of **patient photos**, include a statement confirming that consent to publish was obtained.	Not Applicable	
Studies involving experimental **animals**: State details of **authority granting ethics approval** (IRB or equivalent committee(s), provide reference number for approval. Include a statement of compliance with ethical regulations.	Not Applicable	
Studies involving **specimen and field samples:** State if relevant **permits** obtained, provide details of authority approving study; if none were required, explain why.	Not Applicable	

Dual Use Research of Concern (DURC)	Information included in the manuscript?	In which section is the information available? (Reagents and Tools Table, Materials and Methods, Figures, Data Availability Section)
Could your study fall under dual use research restrictions? Please check biosecurity documents and list of **select agents and toxins** (CDC): https://www.selectagents.gov/sat/list.htm	Not Applicable	
If you used a select agent, is the security level of the lab appropriate and reported in the manuscript?	Not Applicable	
If a study is subject to dual use research of concern regulations, is the name of the **authority granting approval and reference number** for the regulatory approval provided in the manuscript?	Not Applicable	

Reporting

The MDAR framework recommends adoption of discipline-specific guidelines, established and endorsed through community initiatives. Journals have their own policy about requiring specific guidelines and recommendations to complement MDAR.

Adherence to community standards	Information included in the manuscript?	In which section is the information available? (Reagents and Tools Table, Materials and Methods, Figures, Data Availability Section)
State if relevant guidelines or checklists (e.g., **ICMJE, MIBBI, ARRIVE, PRISMA**) have been followed or provided.	Not Applicable	
For **tumor marker prognostic studies,** we recommend that you follow the **REMARK** reporting guidelines (see link list at top right). See author guidelines, under 'Reporting Guidelines'. Please confirm you have followed these guidelines.	Not Applicable	
For **phase II and III randomized controlled trials**, please refer to the **CONSORT** flow diagram (see link list at top right) and submit the CONSORT checklist (see link list at top right) with your submission. See author guidelines, under 'Reporting Guidelines'. Please confirm you have submitted this list.	Not Applicable	

Data Availability

Data availability	Information included in the manuscript?	In which section is the information available? (Reagents and Tools Table, Materials and Methods, Figures, Data Availability Section)
Have **primary datasets** been deposited according to the journal's guidelines (see 'Data Deposition' section) and the respective accession numbers provided in the Data Availability Section?	Not Applicable	
Were **human clinical and genomic datasets** deposited in a public access-controlled repository in accordance to ethical obligations to the patients and to the applicable consent agreement?	Not Applicable	
Are **computational models** that are central and integral to a study available without restrictions in a machine-readable form? Were the relevant accession numbers or links provided?	Not Applicable	
If publicly available data were reused, provide the respective **data citations in the reference list**.	Not Applicable	
